# The two-component system ColRS discriminates metal signals to regulate virulence and resistance in *Pseudomonas aeruginosa*

**DOI:** 10.1371/journal.ppat.1014538

**Published:** 2026-08-28

**Authors:** Ninglin Zhao, Ziqi Zhu, Xiyu Wu, Jianping Ying, Dan Fan, Cheng Nong, Yingjie Song, Tonggen Liu, Chenxi Duan, Yaping Zheng, Shuang Zou, Xingyu Mou, Rui Bao

**Affiliations:** 1 Division of Infectious Diseases in State Key Laboratory of Biotherapy, Center of Infectious Diseases, West China Hospital, Sichuan University, Chengdu, China; 2 Advanced Mass Spectrometry Center, Research Core Facility, Frontiers Science Center for Disease-related Molecular Network, West China Hospital, Sichuan University, Chengdu, China; 3 College of Life Science, Sichuan Normal University, Chengdu, China; University of Utah, UNITED STATES OF AMERICA

## Abstract

Two-component systems (TCSs) in *Pseudomonas aeruginosa* are essential for sensing and responding to diverse environmental cues, including those associated with ion homeostasis and virulence within the host. However, how specific TCSs discriminate between chemically similar metal signals to fine-tune adaptation remains unclear. Here, we identify the TCS ColRS, conserved across the *Pseudomonas* genus, as a critical sensor that distinguishes zinc (Zn^2+^) and copper (Cu^2+^) to drive divergent survival strategies. First, we establish that the response regulator ColR directly binds the promoters of lipid A modification genes (*arnB*, *eptA*), the metal responsive regulator *czcR*, and the alginate and motility regulator Z (*amrZ*), genetically linking metal sensing to resistance and virulence circuitry. Phenotypically, ColRS translates these inputs into context-dependent states: enhancing polymyxin tolerance and suppressing motility under copper stress, while sustaining type III secretion system (T3SS) virulence and motility under zinc stress. Mechanistically, this discrimination relies on the sensor kinase ColS, which utilizes a non-canonical E96-H105 interface as a specificity filter to recognize Cu^2+^ (Kd ≈ 1.11 μM) and distinguish it from Zn^2+^ (Kd ≈ 19.8 μM). Global proteomics confirms this bifurcation, revealing that Zn^2+^ triggers focused metabolic tuning, whereas Cu^2+^ induces broad envelope and motility rewiring. Collectively, our findings reveal ColRS as a pivotal molecular hub that decodes the duality of host metal immunity to optimize pathoadaptation.

## Author summary

Bacterial pathogens encounter fluctuating metal environments during infection, yet how they distinguish chemically similar metal signals and translate them into appropriate adaptive responses remains poorly understood. We show that in *Pseudomonas aeruginosa*, the two-component system ColRS functions as a metal-discriminating sensory module that differentiates between zinc and copper and directs these signals into distinct regulatory programs. At the molecular level, the sensor kinase ColS uses specific coordination residues to differentially engage Zn^2+^ and Cu^2+^. This selectivity is propagated through the response regulator ColR, which directly controls genes involved in lipid A modification, metal homeostasis, motility, and biofilm-associated regulation. System-wide analyses reveal that zinc and copper trigger markedly different proteomic reprogramming patterns through ColRS, resulting in divergent yet convergent phenotypic outcomes that enhance virulence and polymyxin resistance. These findings establish a general principle by which a single bacterial sensory system can decode metal identity to reshape regulatory priorities, providing new insight into how pathogens integrate host-derived signals to coordinate infection strategies and antibiotic tolerance.

## Introduction

The host-pathogen interface presents a fundamental dichotomy of transition metal stress: to restrict microbial growth, hosts deploy nutritional immunity by sequestering essential cofactors such as zinc (Zn^2+^), while simultaneously leveraging metal intoxication exemplified by the bombardment of copper (Cu^2+^) within phagosomes to poison invaders [1–4]. For *Pseudomonas aeruginosa*, an opportunistic pathogen adept at colonizing diverse niches, survival mandates a regulatory logic that goes beyond simple metal sensing [5]; it requires the precise discrimination of chemically similar cues to navigate physiologically opposing demands: scavenging for nutrients (under Zn^2+^ limitation) versus fortifying against toxicity (under Cu^2+^ excess) [6,7]. Failure to distinguish a “starvation signal” (Zn^2+^ limitation) from a “toxicity signal” (Cu^2+^ excess) would lead to maladaptive responses [1, 2], yet how *P. aeruginosa* integrates these inputs to coordinate high-level pathogenic trade-offs remains a critical open question. While dedicated metal responsive pathways in *P. aeruginosa* such as the Zn-responsive CzcRS and Cu-responsive CopRS/CueRS systems are well studied [8–13], they function primarily as specialized detoxification modules that tune local homeostasis and efflux. However, metal stress during infection necessitates system-wide physiological rewiring that extends beyond homeostasis. It remains unclear how pathogens interpret the host’s dynamic metal landscape as a broader ecological signal to orchestrate complex lifestyle decisions, such as balancing energy-expensive virulence factors against envelope fortification and antibiotic resistance. The complexity of this regulation implies that additional, integrative signaling architectures must exist to bridge specific metal cues with global adaptive networks.

The ColRS two-component system represents a compelling candidate for an integrative sensory module linking environmental cues to adaptive responses in *Pseudomonas* [14–16]. Phylogenetic analyses reveal that ColRS is part of the core genome and exhibits near-universal conservation across the *Pseudomonas* genus (>61% amino acid identity), suggesting an evolutionarily stable function distinct from the variable accessory genome [17]. Studies in environmental *Pseudomonads* such as *Pseudomonas putida* have demonstrated that ColRS contributes to tolerance of multiple transition metals, including zinc, iron, manganese, and cadmium, whereby metal excess activates ColRS signaling and alters transcription of ColR-regulated genes [18]. Proteomic analyses further revealed that zinc stress in *P. putida* triggers broad proteome remodeling that is partially ColR-dependent [19]. Beyond metal tolerance, ColR directly regulates genes involved in membrane integrity and stress resistance, and its disruption increases susceptibility to phenol and other envelope stresses [14,16]. Functional homologs in plant-pathogenic bacteria underscore additional roles for ColRS in host association. In *Pseudomonas fluorescens*, ColRS is required for rhizosphere colonization, and in *Xanthomonas citri* it influences virulence, biofilm formation, stress resistance, and expression of key pathogenicity genes [15,17]. In *P. aeruginosa*, prior work has primarily linked ColRS to membrane stress responses and virulence [17,20], leaving its specific role in navigating host-relevant Zn^2+^ and Cu^2+^ fluctuations undefined. Motivated by this disconnect, we investigated whether ColRS functions as the missing link that couples metal sensing to pathoadaptive remodeling.

In this study, we elucidate the ColRS signaling axis as a pivotal decision module. First, we define a direct ColR regulon by integrating biochemical validation with real-time fluorescent quantitative reverse transcription PCR (RT-qPCR), uncovering a genetic link between the system and clinically relevant envelope programs specifically lipid A remodeling (*arnB*, *eptA*) and the metal responsive regulator (*czcR*). This direct regulation provides a mechanistic basis for the observed ColRS-dependent phenotypes. Biophysically, we demonstrate that the sensor kinase ColS discriminates between Zn^2+^ and Cu^2+^ via a non-canonical E96-H105 interface. Crucially, this site acts as a selectivity filter that is strictly required for Cu^2+^ perception while permitting Zn^2+^ engagement. Finally, by combining multi-omics with infection models, we show that ColRS translates this discrimination into divergent survival strategies: sustaining virulence (T3SS) under Zn^2+^ conditions while prioritizing envelope defense and polymyxin resistance under Cu^2+^ stress. Collectively, these findings establish ColRS as a master regulator that decodes the duality of host metal immunity to optimize bacterial fitness.

## Results

### The conserved ColRS two-component system governs basal pathogenicity and biofilm formation in *P. aeruginosa*

**Fig 1:**
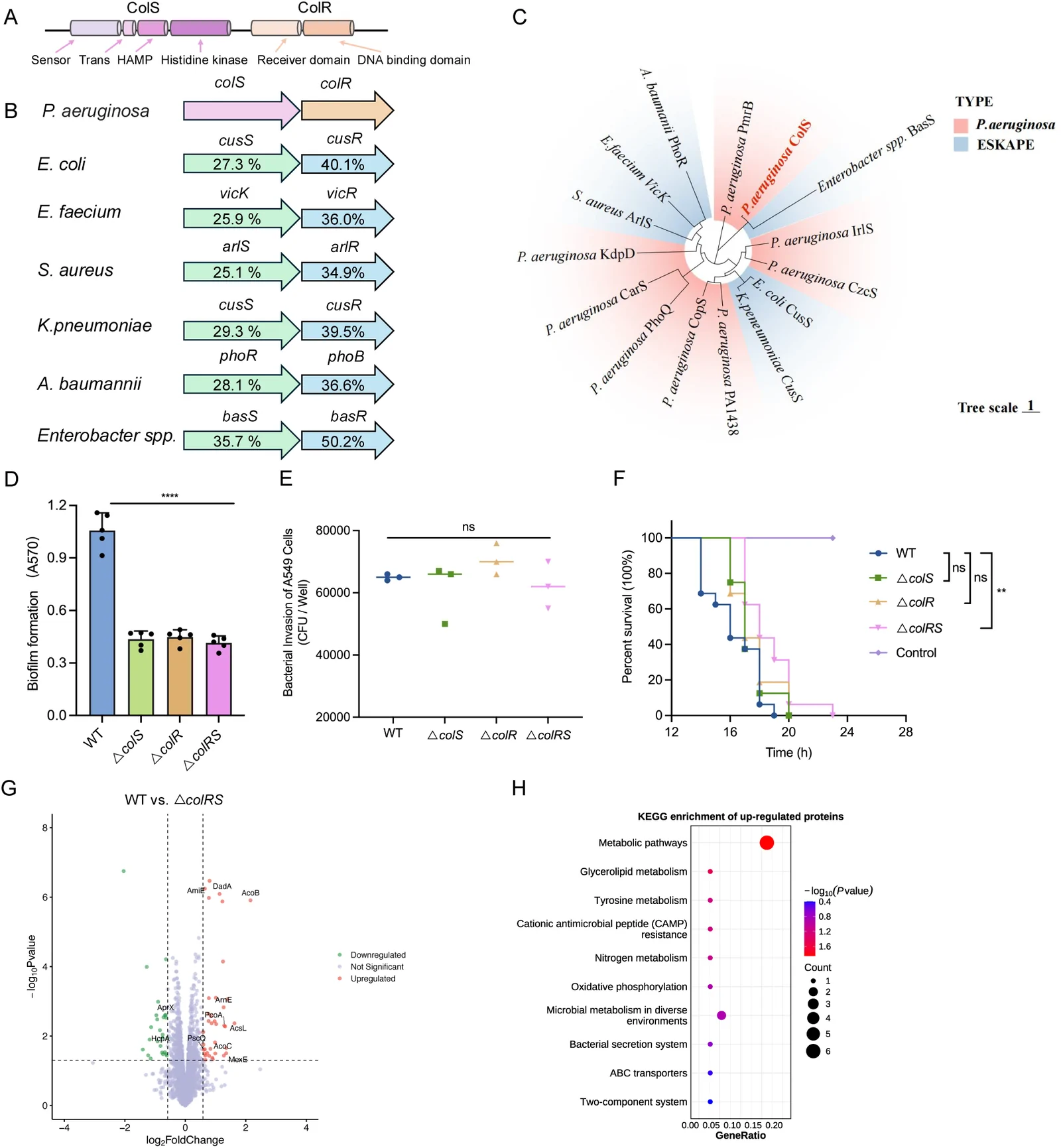
The conserved ColRS two-component system governs basal pathogenicity and biofilm formation in *P. aeruginosa.* (A) Schematic of the ColRS two-component system proteins. ColS consists of sensor, transmembrane (Trans), HAMP, and histidine kinase domains. ColR comprises a receiver domain and a DNA-binding domain. (B) Schematic representation of the ColRS two-component system in *P. aeruginosa* and homologs in other ESKAPE pathogens. (C) Phylogenetic tree of ColS including 15 histidine kinases from *P. aeruginosa* and ESKAPE pathogens. Phylogenetic relationships were inferred using the Neighbor-Joining method, with bootstrap values shown at the nodes. Evolutionary distances were calculated using the Poisson correction method. The analysis was performed using MEGA12 [23]. (D) Biofilm formation in WT and mutant strains in LB medium. (E) Invasion of A549 cells by WT and mutant strains. The cells were infected with *P. aeruginosa* at a multiplicity of infection (MOI) of 10 for 1 h. Bacteria were cultured in M9 medium without Zn^2+^ or Cu^2+^ supplementation prior to infection. (F) Virulence of WT and mutant strains in the *G. mellonella* infection model. Bacteria were cultured in M9 medium without Zn^2+^ or Cu^2+^ supplementation prior to infection. The survival rates of larvae were monitored for 24 h after the infection. The experiments were performed three times. (G) Volcano plot displaying the proteomic profiles of WT and Δ*colRS* strains. The significantly up and down-regulated proteins are labeled with red and green, respectively. (H) The up-regulated proteins were categorized by KEGG enrichment functional category. The statistical comparisons were performed using one-way ANOVA or Mantel-Cox (log-rank) test. *P < 0.05, **P < 0.01, ***P < 0.001, ****P < 0.0001, ns, not significant.

To assess the functional significance of ColRS in *P. aeruginosa*, we first examined its evolutionary trajectory and structural conservation. ColS belongs to classical histidine kinases protein characterized by a periplasmic sensor domain for signal recognition flanked by two transmembrane helices and an intracellular histidine kinases domain. ColR consists of an N-terminal regulatory receiver domain and a C-terminal OmpR/PhoB-type domain for DNA binding [21] (Fig 1A). Within the *Pseudomonas* genus, ColS and ColR orthologs display strong sequence conservation across species, as shown by Clustal Omega alignment (S1 Fig), suggesting functional conservation of the ColRS system. Phylogenetic analyses demonstrate that ColRS is not unique to *Pseudomonas* but shares significant homology with two-component systems (TCSs) in other critical ESKAPE pathogens (*Enterococcus faecium*, *Staphylococcus aureus*, *Klebsiella pneumoniae*, *Acinetobacter baumannii*, *Pseudomonas aeruginosa Enterobacter spp*.) [22,23] (ColS, ~ 25–35% identity; ColR, ~ 35–50% identity; Figs 1B-1C and S2A), suggesting an ancient, evolutionarily constrained role in bacterial adaptation. Analyses of 731 clinical *P. aeruginosa* genomes further show that the ColRS locus is nearly invariant, with >98.1% predicted amino-acid identity observed across diverse sequence types (S2B Fig) [24]. The high degree of sequence conservation suggests that, unlike variable accessory traits, ColRS likely performs a core function essential for *P. aeruginosa* fitness across heterogeneous host environments.

To define the physiological scope of ColRS, we generated in-frame deletion mutants (Δ*colS*, Δ*colR* and Δ*colRS*) under standard laboratory conditions. We reasoned that if ColRS is a stress-response system, it might be dispensable for basic vegetative growth. Consistently, all mutants exhibited wild-type (WT) growth kinetics in minimal (M9) and Luria-Bertani (LB) medium and produced comparable levels of the phenazine toxin pyocyanin and the siderophore pyoverdine (S2C–S2F Fig). Furthermore, antibiotic susceptibility profiling revealed that under unstimulated conditions, the minimum inhibitory concentrations (MICs) for a panel of clinically relevant antibiotics remained largely unchanged in the mutants (S1 and S2 Tables). However, obvious defect was observed in surface colonization: the Δ*colS,* Δ*colR* and Δ*colRS* mutant displayed a significant ~60% reduction in biofilm biomass compared to the WT (Fig 1D). While swarming and swimming motilities showed only minor, non-significant fluctuations (S2G, S2H Fig), the specific biofilm defect suggests ColRS is critical for the sessile lifestyle transition. In host-interaction models, while invasion of A549 epithelial cells was unaffected (Fig 1E), the *Galleria mellonella* infection model showed a subtle trend toward prolonged survival in mutant-infected larvae (median survival: WT 16 h vs. Δ*colRS* 18 h; Fig 1F). Collectively, these data indicate that while ColRS is dispensable for core metabolism and acute toxicity in naive environments, it is selectively required for establishing surface colonization, hinting that its full regulatory potential is likely triggered by specific host-associated cues.

To dissect the molecular architecture underlying the ColRS-dependent biofilm defect and identify its basal regulatory footprint, we performed quantitative proteomics comparing WT and Δ*colRS* strains during exponential growth (n = 4). Principal component analysis (PCA) confirmed distinct proteomic profiles, and using stringent cutoffs (Fold Change > 1.5, *P*. value < 0.05), we identified 63 differentially expressed proteins (DEPs) (37 increased and 26 decreased in Δ*colRS*; Fig 1G and S4 Table). Functional network analysis revealed that ColRS orchestrates a specific subset of the envelope stress response rather than a global metabolic shift. KEGG enrichment indicated that ColRS-dependent changes were concentrated in three key functional clusters (Fig 1H): (i) envelope defense and lipid A modification: we observed up‑regulation of ArnE and LipA, components of the pathway responsible for aminoarabinose modification of Lipid A [25]. The basal upregulation of these intrinsic resistance determinants in the absence of antibiotic pressure suggests ColRS normally constrains outer membrane defense programs, preventing their inappropriate activation under non-stress conditions [20]. (ii) metal homeostasis: the copper resistance protein PcoA [26] and the acetyl-coa synthetase AcoB, AcoC and AcsL [27] were significantly upregulated in the Δ*colRS* strain. The spontaneous enrichment of metal-handling proteins in the basal regulon provided the clue that ColRS might possess an intrinsic metal-sensing capability. (iii) biofilm and virulence factors: consistent with the observed biofilm deficiency (Fig 1D) and attenuated virulence phenotype (Fig 1F) in the double mutant, levels of HcpA, a core structural and effector component of the type VI secretion system [28], and AprX, a secreted serine protease implicated in host tissue invasion and nutrient acquisition [29] were downregulated in the Δ*colRS* strain. In addition, ChoS (a cholesterol oxidase potentially involved in utilization of host-derived carbon sources) and AtsA (an arylsulfatase associated with sulfate ester metabolism and bacterial adaptation to host environments) were also decreased. Collectively, this proteomic landscape indicates that ColRS acts as a central basal regulator that simultaneously restrains envelope defense and metal response pathways while sustaining the expression of key colonization and virulence factors.

### ColR directly regulates a key regulon linking resistance and virulence

**Fig 2:**
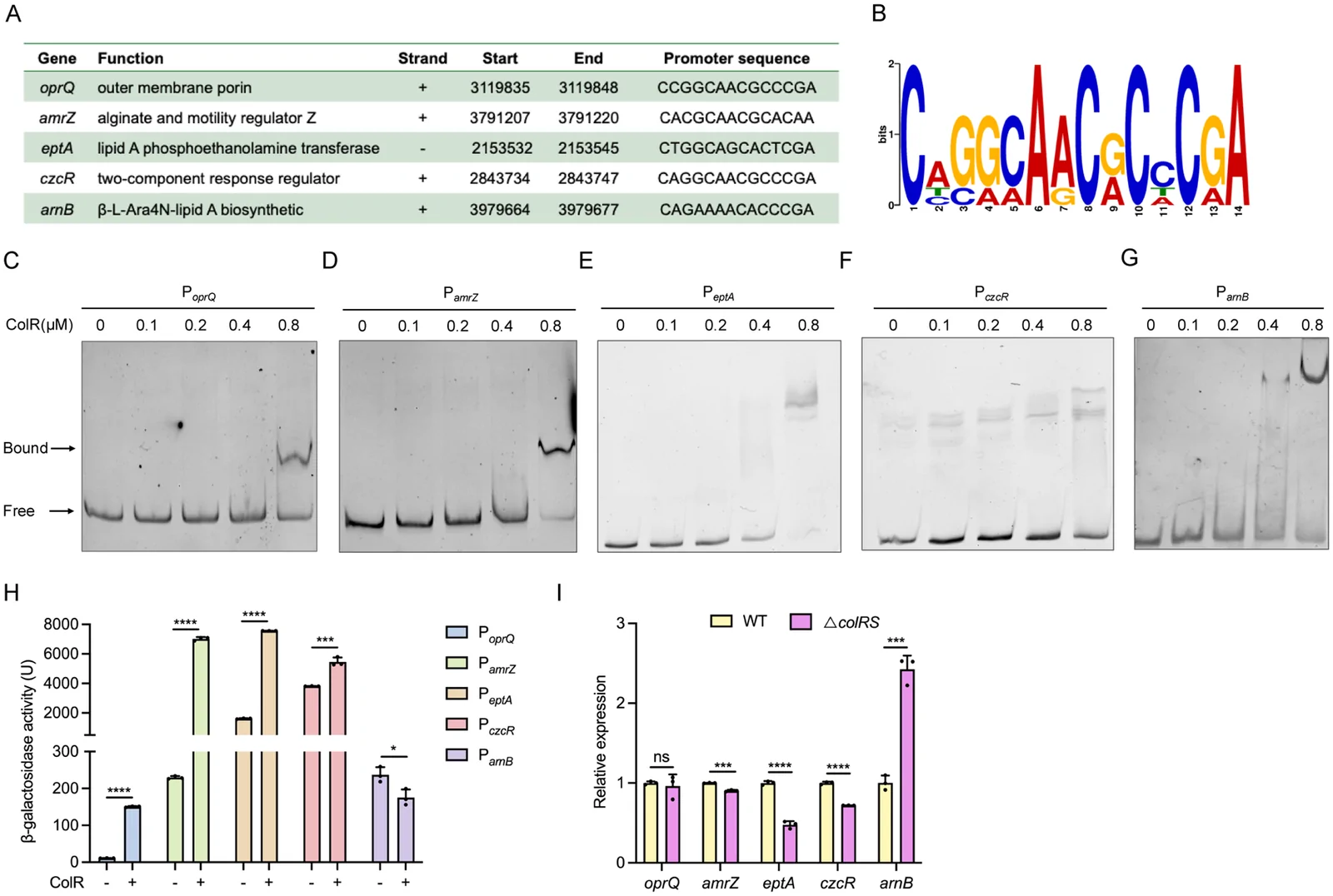
ColR directly regulates a key regulon linking resistance and virulence. (A) Functional categorization of genes directly regulated by ColR in *P. aeruginosa*. The ColR-binding motifs identified in the tested promoters are indicated. (B) Conservation analysis of ColR-binding promoter motifs. (C-G) Electrophoretic mobility shift assays (EMSA) demonstrating the binding of ColR to five target promoters. The final DNA concentration was 0.1 μM, and ColR protein was titrated from 0 to 0.8 μM. (H) Transcriptional activities of the five ColR-bound promoters were measured using a β-galactosidase reporter system. Experiments were performed three times, and data are presented as mean ± SD (error bars). (I) Relative expression levels of the five target genes in WT and Δ*colRS* strains were determined by RT-qPCR. Experiments were performed at least three times, and representative results from one independent experiment are shown. The statistical comparisons were performed using Student's *t* test. *P < 0.05, **P < 0.01, ***P < 0.001, ****P < 0.0001, ns, not significant.

Building on the basal biofilm defect and selective proteomic reprogramming observed in the Δ*colRS* mutant, we next sought to determine whether these phenotypes stem from direct transcriptional regulation by the response regulator ColR. To define the direct targets of ColR, we conducted a genome-wide scan in *P. aeruginosa* PA14, guided by previously reported ColR-binding motifs and relevant literature [17–20,30]. From this analysis, we curated a set of candidate promoter regions representing three key functional categories for experimental validation: cell envelope modification and antibiotic resistance (*arnB, pagL, eptA, pap2, dgkA, oprQ*) [31–33]; metal homeostasis and stress response (*czcR, czcC, kdpE, kdpD, kdpF*) [8,34]; and virulence and adaptive regulation (*amrZ, algD, warA*) [17,35–37]. To test whether ColR directly associates with these loci, we performed electrophoretic mobility shift assays (EMSA) using purified ColR protein and promoter fragments corresponding to the candidate genes. Among the promoters tested, five (~35%) exhibited clear, concentration-dependent mobility shifts, indicative of specific ColR-DNA interactions. A putative ColR-binding motif identified from promoter analysis revealed a high degree of conservation across target promoters (Fig 2A-2G). These direct targets included *eptA* and *arnB*, which encode enzymes that modify lipid A to reduce the outer membrane’s negative charge and contribute to polymyxin resistance [20] (Fig 2E, 2G); *amrZ*, a transcriptional regulator of alginate biosynthesis and motility [36] (Fig 2D); *czcR*, the response regulator of a Zn^2+^/Co^2+^/Cd^2+^ efflux system [8,38] (Fig 2F); and *oprQ*, an outer membrane porin involved in nutrient uptake and permeability [39] (Fig 2C). In contrast, no detectable binding was observed for the remaining nine promoters tested (S3 Fig). Notably, these genes are functionally linked to envelope architecture, metal efflux regulation, and biofilm or virulence-related control, consistent with the phenotypic and proteomic signatures of ColRS deficiency.

To evaluate whether ColR binding translates into functional transcriptional regulation, we quantified promoter activity using β-galactosidase reporters in a heterologous *Escherichia coli* system under basal growth conditions. In this context, ColR predominantly acted as a transcriptional activator: compared to the empty vector control, ColR increased the activity of *amrZ*, *oprQ*, *eptA*, and *czcR* promoters by approximately 3- to 15-fold (Fig 2H). In contrast, ColR repressed the *arnB* promoter, resulting in ~40% reduction in reporter activity, demonstrating that ColR can exert bidirectional transcriptional control (Fig 2H). To confirm these regulatory relationships in the native host, we performed RT-qPCR on *P. aeruginosa* WT and Δ*colRS* strains. Consistent with the reporter assays, transcript levels of *amrZ*, *eptA*, and *czcR* were significantly reduced in the Δ*colRS* mutant, whereas *arnB* expression was derepressed relative to WT (Fig 2I). Although ColR bound the *oprQ* promoter DNA and *oprQ* was activated by ColR in reporter assays, its steady-state transcript abundance did not differ significantly in the Δ*colRS* mutant under basal conditions, suggesting that full transcriptional regulation of this promoter may depend on additional *P. aeruginosa*-specific cofactors or environmental signals.

### Host-associated metals differentially tune ColRS expression and drive metal-specific phenotypic plasticity

**Fig 3:**
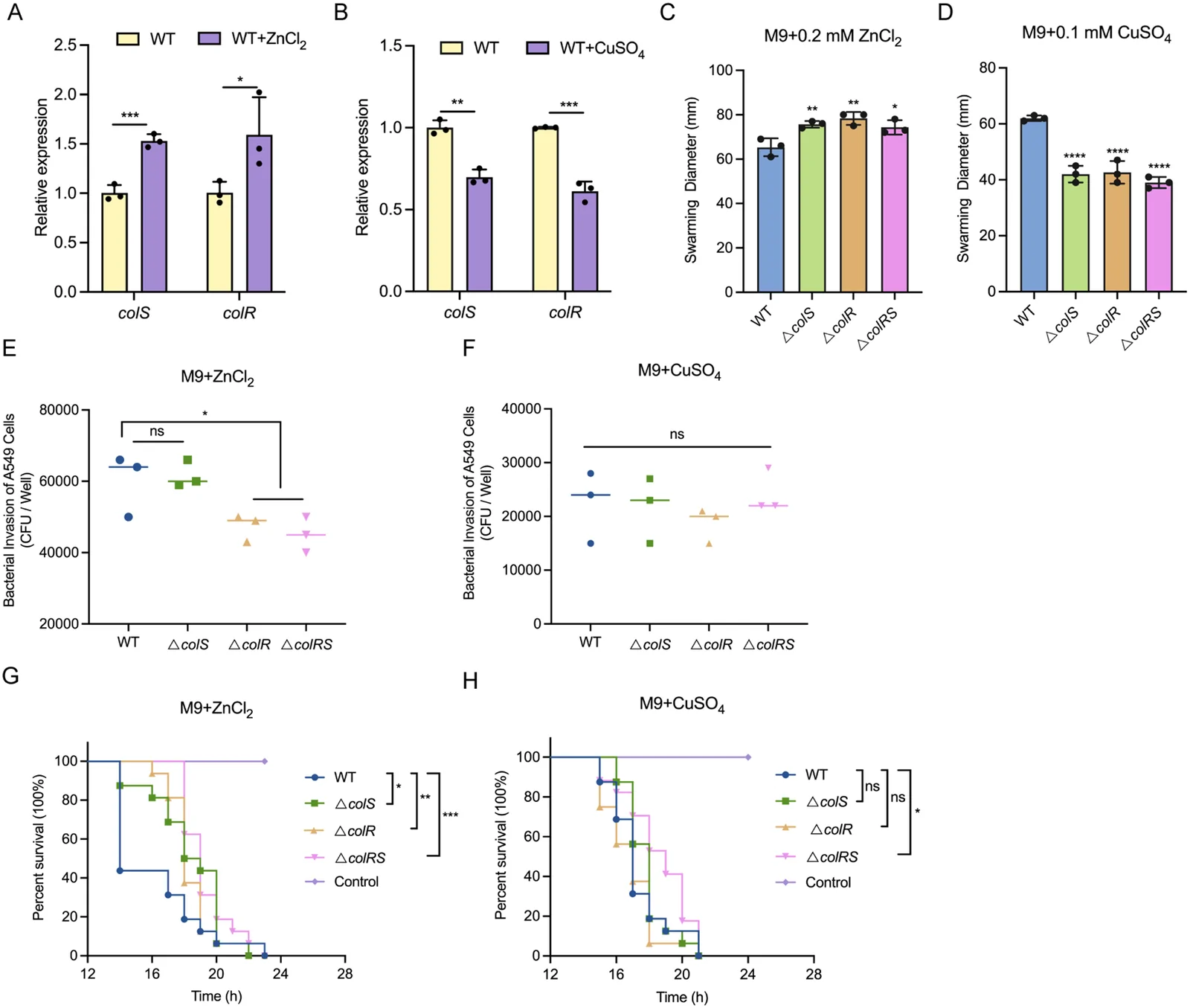
Host-associated metals differentially tune ColRS expression and drive metal-specific phenotypic plasticity. (A-B) Relative expression levels of *colS* and *colR* measured by RT-qPCR following treatment with 0.2 mM zinc (A), 0.2 mM copper (B) in M9 medium. (C-D) Swarming motility of WT and Δ*colS*, Δ*colR*, Δ*colRS* mutant strains following treatment with 0.2 mM zinc (C), 0.1 mM copper (D). Data are from three independent experiments and presented as the average ± SD (error bar). (E-F) Bacterial invasion assay of WT and mutant strains following treatment with 0.2 mM zinc (E) or 0.2 mM copper (F) in A549 cells. Cells were infected with *P. aeruginosa* at a multiplicity of infection (MOI) of 10 for 1 h. (G-H) Virulence of WT and mutant strains following treatment with 0.2 mM zinc (G) or 0.2 mM copper (H) in a *G. mellonella* infection model. Larval survival was monitored for 24 h after infection. Experiments were performed at least three times, and representative results from one independent experiment are shown. Statistical comparisons were performed using one-way ANOVA, Student's *t* test or Mantel-Cox (log-rank) test. *P < 0.05, **P < 0.01, ***P < 0.001, ****P < 0.0001, ns, not significant.

The identification of *czcR* as a direct ColR target, together with previous reports linking ColRS homologs to metal tolerance in *P. putida*, suggests that the ColRS system participates in metal-responsive regulation [18,19]. In the context of host immunity, transition metals such as Zn^2+^ and Cu^2+^ assume distinct physiological roles: Zn^2+^ is essential but can be sequestered to impose nutritional limitation, whereas Cu^2+^ is actively mobilized at infection sites to exert toxicity [6]. Based on these contrasting roles, we reasoned that ColRS may differentiate between these host-relevant metal cues and adopt distinct regulatory states in response. To evaluate this possibility, we first examined the growth of WT and ColRS mutants across a range of Zn^2+^ and Cu^2+^ concentrations. Under the conditions used in our assays, growth of the WT, Δ*colS*, Δ*colR*, and Δ*colRS* strains was markedly inhibited as the concentrations of Zn^2+^ or Cu^2+^ in M9 medium increased (up to 500 μM). However, no differences in growth were observed among the strains (S4 Fig). Considering that this concentration range might be insufficient to reveal differences in metal tolerance, we further determined the minimum inhibitory concentrations (MICs) of Zn^2+^ and Cu^2+^ in M9 medium. The MIC values were identical among WT, Δ*colS*, Δ*colR*, or Δ*colRS* strains, with Zn^2+^ and Cu^2+^ MICs of 4 mM and 1 mM, respectively (S3 Table). These results indicating that deletion of *colS*, *colR*, or the *colRS* system does not affect resistance to these metals under our experimental conditions. We next investigated whether Zn^2+^ and Cu^2+^ differentially influence ColRS expression. Further RT-qPCR revealed that exposure to 0.2 mM Zn^2+^ upregulated transcript levels of both *colS* and *colR*, whereas 0.2 mM Cu^2+^ exposure repressed their expression (Fig 3A, 3B).

Given that ColR directly controls genes involved in lipid A modification, including *arnB* and *eptA*, which are known to influence outer membrane charge and polymyxin susceptibility [20], we next assessed whether these metal signals modulate ColRS-dependent antibiotic resistance. In M9 medium supplemented with 0.2 mM CuSO_4_, Δ*colS*, Δ*colR*, and Δ*colRS* mutants exhibited a 2-fold increase in susceptibility to polymyxin B and a 4-fold increase in susceptibility to colistin compared to WT (Table 1). ColRS mutants also showed a similar 2- to 4-fold increase in polymyxin B and colistin sensitivity in the presence of 0.2 mM ZnCl_2_ (Table 2). In contrast, MIC for amikacin, tobramycin, meropenem, and ciprofloxacin remained largely unchanged under the same metal conditions (Tables 1 and 2), indicating that the observed effect is not a generalized loss of antibiotic resistance but rather a specific enhancement of sensitivity to envelope-active polymyxins. Although Zn^2+^ and Cu^2+^ exert opposite effects on *colS* and *colR* gene expression, both metals produced similar trends in ColRS-dependent antibiotic tolerance. This apparent paradox suggests that Zn^2+^ and Cu^2+^ may activate distinct ColRS signaling cascades that converge on downstream pathways affecting outer membrane defense and antibiotic susceptibility. Under copper conditioning (0.1 mM CuSO_4_), ColRS deficiency suppressed swarming behavior, whereas under Zn^2+^ conditioning (0.2 mM ZnCl_2_), ColRS deficiency promoted swarming (Figs 3C, 3D and S5). Although, we did not observe corresponding metal-dependent trends in swimming motility under the same conditions (S5 Fig). These opposing motility outputs indicate that ColRS governs phenotypic responses that are contingent on specific metal cues, rather than executing a uniform, stereotyped response to general stress.

**Table 1:** MICs of different antibiotics against WT and mutant strains in the presence of CuSO_4_.

MIC (g/mL)						
M9 medium	Amikacin	Tobramycin	Polymyxin B	Colistin	Meropenem	Ciprofloxacin
PA14	4	1	2	8	>16	1
△*colS*	4	1	1	2	>16	1
△*colR*	4	1	1	2	>16	1
△*colRS*	4	1	1	2	>16	1

**Table 2:** MICs of different antibiotics against WT and mutant strains in the presence of ZnCl_2_.

MIC (g/mL)						
M9 medium	Amikacin	Tobramycin	Polymyxin B	Colistin	Meropenem	Ciprofloxacin
PA14	8	1	8	>16	0.5	1
△*colS*	8	1	4	16	0.5	1
△*colR*	8	1	4	16	0.5	1
△*colRS*	8	1	2	8	0.5	1

Metal conditioning also shaped ColRS-dependent virulence phenotypes in a manner sensitive to the identity of the metal ion. In the A549 epithelial intracellular survival assay, exposure to Zn^2+^ revealed a clear requirement for ColR: Δ*colR* and Δ*colRS* mutants exhibited approximately 25% lower intracellular survival compared with the WT strain (Fig 3E). Under Cu^2+^ treatment, mutant strains trended toward reduced intracellular survival but did not reach statistical significance (Fig 3F), suggesting that ColRS contributions to intracellular persistence are preferentially engaged under zinc-conditioned conditions. Consistent with this bias, outcomes in the *Galleria mellonella* infection model were also metal-dependent. In the presence of Zn^2+^, median survival times for WT, Δ*colS*, Δ*colR* and Δ*colRS* strains were 14 h, 18.5 h, 18 h and 19 h, respectively (Fig 3G), indicating that Zn^2+^ conditioning amplifies the impact of ColRS on systemic virulence regulation. Under Cu^2+^ exposure, median survival times for WT, Δ*colS*, Δ*colR*, and Δ*colRS* strains were 17 h, 18 h, 17 h and 19 h, respectively, with the Δ*colRS* mutant exhibiting the most pronounced survival advantage (Fig 3H). These findings imply that while ColRS contributes to resisting host clearance under both metal conditions, the mechanistic emphasis of this contribution differs depending on the encountered metal ion. Collectively, these results indicate that ColRS drives metal-specific phenotypic plasticity, coordinating envelope defense, motility, and virulence to tailor bacterial behavior to host-associated metal ion encountered.

### ColS discriminates zinc and copper via a non-canonical E96-H105 motif

**Fig 4:**
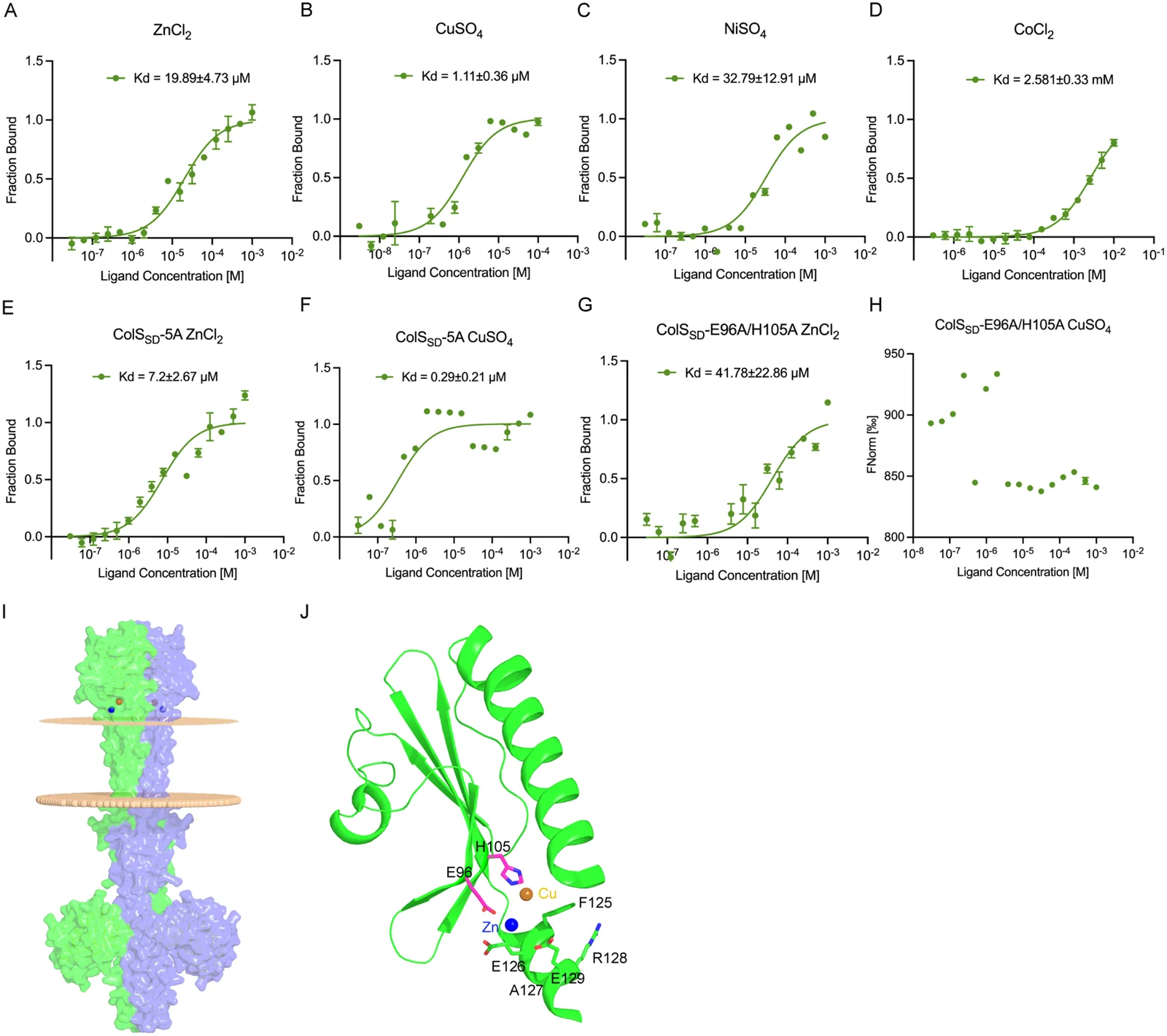
ColS discriminates zinc and copper via a non-canonical E96-H105 motif. (A-D) Binding affinity of the ColS_SD_ (sensor domain) for zinc (A), copper (B), nickel (C), or cobalt (D) ions, as determined by Microscale Thermophoresis (MST). (E-F) Binding affinity of the ColS_SD_ mutant (ColS_SD_-5A) for zinc (E) and copper (F) ions, as measured by Microscale Thermophoresis (MST).(G-H) Binding affinity of the ColS_SD_ double mutant (ColS_SD_-E96A/H105A) for zinc (G) and copper (H) ions, as measured by Microscale Thermophoresis (MST).The experiment was performed at least three times. (I-J) Surface and cartoon representation of the AlphaFold3 structure of the ColS. Key residues involved in metal ion coordination FEARE motif, and E96-H105, are depicted as sticks.

To investigate the molecular mechanisms by which ColRS drives distinct, metal-conditioned phenotypes, we examined how the sensor kinase ColS recognizes and differentiates between chemically similar divalent metal ions (Fig 4). We purified the periplasmic sensor domain of ColS (ColS_SD_) and used microscale thermophoresis (MST) to quantify its interactions with a panel of biologically relevant divalent cations. ColS_SD_ bound Zn^2+^ with a dissociation constant of approximately ~19.89 µM and exhibited markedly higher apparent affinity for Cu^2+^ (Kd ~ 1.11 µM), suggesting that ColS may preferentially engage copper over zinc in the host environments (Fig 4A, 4B). Among other tested divalent metals, Ni^2+^ showed a measurable interaction with ColS_SD_ (Kd ~ 32.79 µM; Fig 4C), whereas interaction with Co^2+^ was comparatively weak (Kd ~ 2.58 mM; Fig 4D). Furthermore, no detectable binding was observed for Ca^2+^, Mg^2+^, Fe^2+^, or Mn^2+^ across the conditions evaluated (S6A–S6D Fig). These results indicate that ColS selectively engages a subset of physiologically relevant divalent cations with substantially different affinities, consistent with its role as a metal-responsive sensor exhibiting ion selectivity rather than as a broad divalent-ion receptor.

To explore potential structural determinants of the ability of ColS to recognize Zn^2+^ versus Cu^2+^, we first examined the contribution of a conserved predicted zinc binding FEARE motif annotated in ColS homologs in *Pseudomonas* [17,18] (S1A Fig). To assess the contribution of the FEARE motif to metal recognition, we generated ColS_SD_-5A by alanine-scanning substituted residues 125–129 (FEARE) (F125A/E126A/A127A/R128A/E129A). MST experimental results show that ColS_SD_-5A variant did not exhibit reduced detectable metal binding, instead, it showed increased apparent affinities for both Zn^2+^ (Kd ~ 7.2 µM) and Cu^2+^ (Kd ~ 0.29 µM) relative to the WT sensor domain (Fig 4E, 4F). These findings indicate that the canonical FEARE motif itself is not the primary determinant of Zn^2+^/Cu^2+^ recognition by ColS, it may serve a modulatory role that influences metal engagement.

To identify additional residues that might contribute to the metal recognition capacity of ColS, we employed AlphaFold3 [40] guided structural modeling of the ColS in the presence of Zn^2+^ or Cu^2+^ (model confidence metrics are provided in S6E, S6F Fig). The resulting models suggested that residues E96 and H105 are positioned within a non-canonical coordination environment that is distinct from the conserved FEARE motif (Fig 4G-4J). Although this predicted region does not constitute a resolved structural site, its geometry and chemical characteristics make it a plausible contributor to interactions with divalent cations. To probe the functional relevance of these residues, we constructed a double mutant (ColS_SD_-E96A/H105A) and reassessed metal binding using MST. Notably, the E96A/H105A substitution completely abolished detectable Cu^2+^ binding, while retaining partial interaction with Zn^2+^, albeit with a roughly two-fold lower apparent affinity (Kd ~ 41.78 µM) compared with the WT sensor domain (Fig 4G, 4H). These data indicate that residues E96 and H105 are required for robust Cu^2+^ engagement and also influence Zn^2+^ interaction, though to a lesser extent. Importantly, the sensor domain construct and all mutant variants displayed comparable purity and solution behavior, with similar size-exclusion chromatography elution profiles and SDS-PAGE patterns, indicating no gross defect in folding or stability (S6G, S6H Fig). Taken together, these results demonstrate that ColS discriminates Zn^2+^ and Cu^2+^ through a non-canonical set of amino acid determinants that differs from the classical FEARE motif.

### Proteomic profiling reveals a hierarchical ColRS regulatory network under distinct metal stress

**Fig 5:**
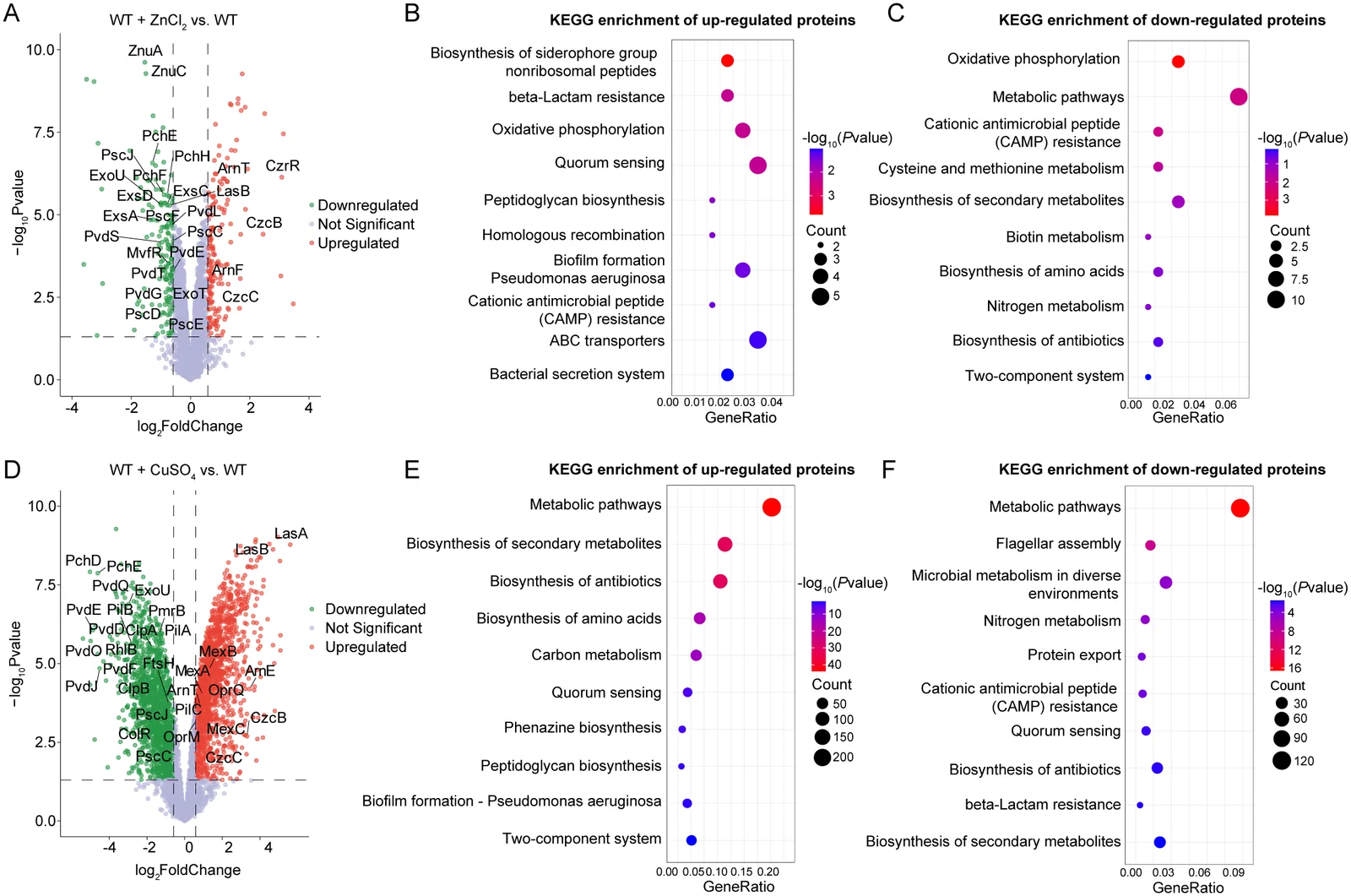
Proteomic profiling reveals zinc- and copper-responsive networks in *P. aeruginosa.* (A) Volcano plot displaying the proteomic profiles of WT and WT supplemented with 0.2 mM Zn^2+^ cultured in M9 medium. The significantly up and down-regulated proteins are labeled with red and green, respectively. (B-C) The related up-regulated (B) and down-regulated (C) proteins were categorized by KEGG enrichment functional category. (D) Volcano plot displaying the proteomic profiles of WT and WT supplemented with 0.2 mM Cu^2+^ cultured in M9 medium. The significantly up and down-regulated proteins are labeled with red and green, respectively. (E-F) The related up-regulated (E) and down-regulated (F) proteins were categorized by KEGG enrichment functional category. Proteomic analyses were performed using four biological replicates. Student's *t* test was used to determine the significance of differentially expressed proteins (DEPs). Proteins with a significance level of *P* < 0.05 and a fold change of ≥ 1.5 or ≤ 0.67 were defined as DEPs.

To dissect how *P. aeruginosa* adapts to host-associated metal stress and how Zn^2+^ and Cu^2+^ differentially condition ColRS-dependent pathways at a systems level, we performed global proteomic profiling of WT exposed to these metals. All strains were cultured in M9 medium supplemented with 0.2 mM Zn^2+^ or Cu^2+^. These conditions were selected based on preliminary growth assays, which showed that 0.2 mM Zn^2+^ did not affect bacterial growth, whereas 0.2 mM Cu^2+^ only slightly inhibited growth. Under 0.2 mM Zn^2+^ stimulation, the proteome exhibited a focused reprogramming, with 334 DEPs (Fig 5A and S4 Table). Among these, 167 proteins were significantly upregulated (Fig 5B), and functional enrichment highlighted involvement in energy metabolism, including oxidative phosphorylation components such as CoxA, CoxB, and NuoJ [41], as well as specific defense systems including cationic antimicrobial peptide resistance factors ArnF and ArnT [31, 42]. Concurrently, 167 proteins were significantly downregulated (Fig 5C), enriched in processes such as siderophore biosynthesis (e.g., PchB, PchE, PchF) [43], quorum sensing (e.g., MvfR, LasB) [44,45], and type III secretion system components (e.g., PscJ, PscC) [46]. Notably, although 0.2 mM Cu^2+^ caused a mild growth inhibition, it triggered a more extensive proteomic remodeling compared to Zn^2+^. A total of 2,743 DEPs were identified under Cu^2+^ conditions (Fig 5D), with 1,374 proteins upregulated (Fig 5E), including factors associated with membrane modification, defense mechanisms, and secondary metabolism such as ArnT, ArnE[31], and the virulence-related protein LasA and LasB[45]. Another 1,369 proteins were downregulated (Fig 5F), with enrichment across diverse core metabolic pathways (e.g., PchD, PchE, PvdJ, RhlB) [43,47]. These contrasting proteomic landscapes mirror the corresponding phenotypes observed under Zn^2+^ and Cu^2+^ exposure, including metal-conditioned changes in polymyxin tolerance. To identify shared and metal-specific components of these responses, we examined proteins similarly affected by Zn^2+^ and Cu^2+^, revealing a core set of conserved modules (S7A, S7B Fig) that include proteins involved in iron uptake, oxidative phosphorylation, quorum sensing, and antibiotic tolerance. Taken together, Zn^2+^ elicits a proteome shift centered on energy metabolism and specific defenses, whereas Cu^2+^ drives broad, multi-layered remodeling affecting both core metabolic and defense networks.

**Fig 6:**
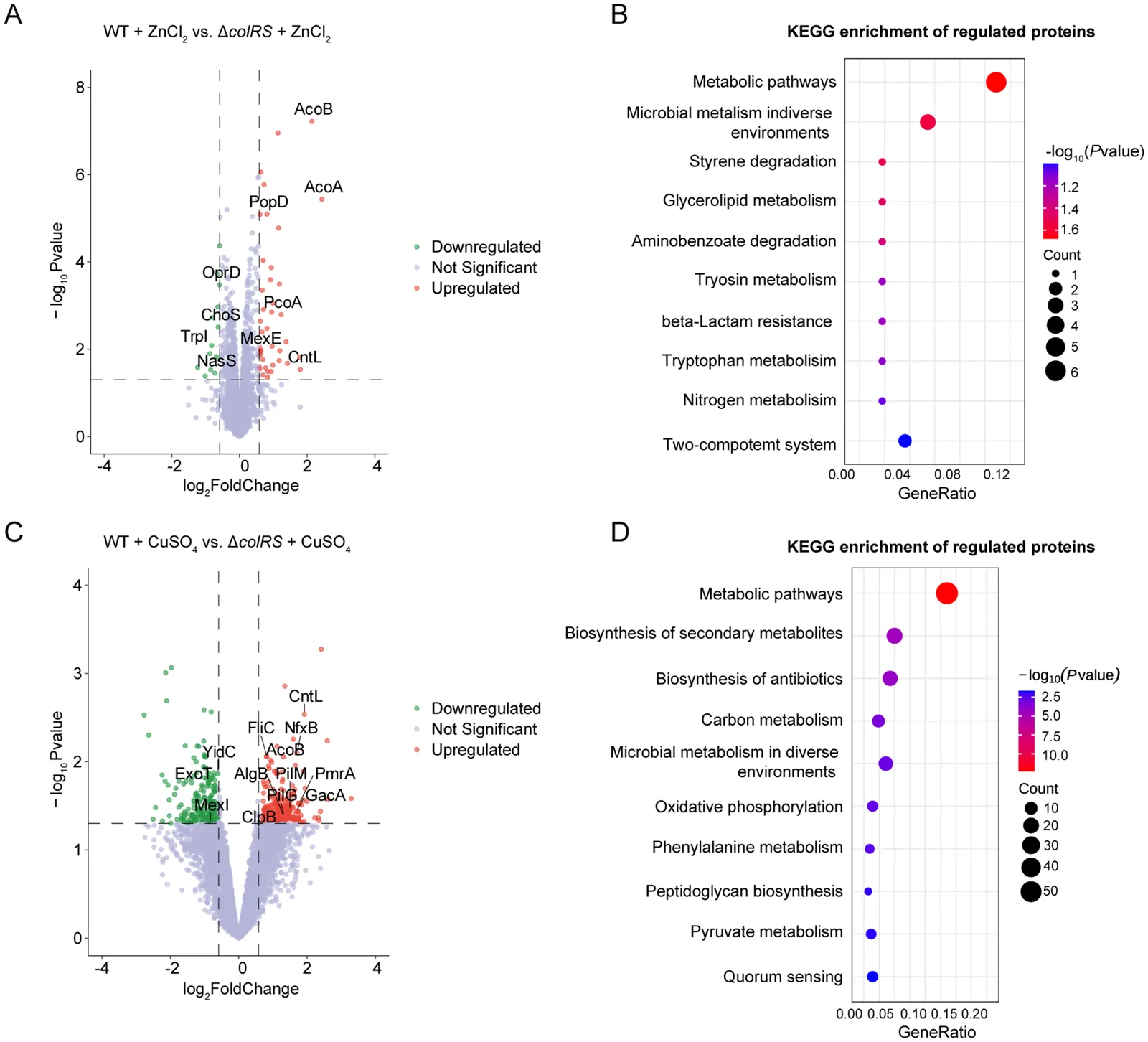
Proteomic profiling reveals a hierarchical ColRS regulatory network under distinct metal stress. (A) Volcano plot displaying the proteomic profiles of WT and Δ*colRS* supplemented with 0.2 mM Zn^2+^ cultured in M9 medium. The significantly up and down-regulated proteins are labeled with red and green, respectively. (B) The related DEPs were categorized by KEGG enrichment functional category. (C) Volcano plot displaying the proteomic profiles of WT and Δ*colRS* supplemented with 0.2 mM Cu^2+^ cultured in M9 medium. The significantly up and down-regulated proteins are labeled with red and green, respectively. (D) The related DEPs were categorized by KEGG enrichment functional category. Student's *t* test was used to determine the significance of differentially expressed proteins (DEPs). Proteins with a significance level of *P* < 0.05 and a fold change of ≥ 1.5 or ≤ 0.67 were defined as DEPs.

To further delineate the regulatory networks governed by the ColRS system under distinct metal stress, we compared the proteomes of WT and Δ*colRS* strains in response to Zn^2+^ and Cu^2+^ exposure. These comparisons revealed a pronounced metal-dependent regulatory landscape: under Zn^2+^ stress, ColRS controlled a relatively compact set of 55 DEPs, whereas under Cu^2+^ stress the ColRS regulon expanded dramatically to encompass 425 DEPs, with both breadth and functional diversity far exceeding the Zn^2+^ condition (Figs 6, S7C and S4 Table). Under Zn^2+^ exposure, although the number of ColRS-dependent proteins was limited, these proteins exhibited clear functional enrichment (Fig 6A, 6B). In the Δ*colRS* strain, the outer membrane porin OprD was downregulated, consistent with altered outer membrane properties under zinc exposure. At the same time, components of the multidrug efflux system (such as MexE) [48] and oxidative stress-related enzymes (such as MdaB) [49] were significantly upregulated, suggesting that in the absence of ColRS, efflux and redox defense pathways become dysregulated and are induced in a compensatory manner during zinc stress. Concurrently, expression of virulence-linked factors including the type III secretion translocator PopD [50] and regulatory protein Pra [51] was increased. Notably, rather than reflecting enhanced pathogenic potential, this induction likely represents a stress-induced compensatory response triggered by disrupted virulence regulation. Despite increased abundance of these virulence-associated proteins, Δ*colRS* exhibited significantly reduced virulence phenotypes, indicating that ColRS is indispensable for integrating virulence factor expression into a functional pathogenic output under Zn^2+^ conditions (Fig 3E, 3G). In contrast, under Cu^2+^ challenge the absence of ColRS led to significant downregulation across multiple functional categories (Figs 6C, 6D and S7C). Resistance networks were broadly affected, with reduced expression of multidrug efflux components (e.g., NorM, MexI) [48], iron uptake outer membrane receptor (e.g., PhuR) [52], heavy-metal tolerance regulators (e.g., FecA, MgtA) [53], and lipopolysaccharide (LPS) transporter (e.g., MsbA) [54], indicating that ColRS coordinates both antibiotic and metal resistance under copper stress. Proteins involved in motility and chemotaxis such as the flagellar structural protein FliC [55], pilus assembly factor PilM, PilG [56], and chemotaxis regulator CheB [57] were also upregulated in Δ*colRS*. However, this increase did not translate into enhanced motility at the phenotypic level, indicating that ColRS is required for the coordinated and productive deployment of motility and chemotaxis machinery under Cu^2+^ stress. Additionally, several virulence-associated regulatory nodes, including the type III secretory system (T3SS) effector proteins ExoT [58] and the pyocyanin synthesis protein PhzM [59], were downregulated, further highlighting the integrated control exerted by ColRS over virulence programs in the copper environment. These proteomic patterns are consistent with our phenotypic findings of ColRS-dependent enhanced resistance and virulence under Cu^2+^ (Fig 3F, 3H).

**Fig 7:**
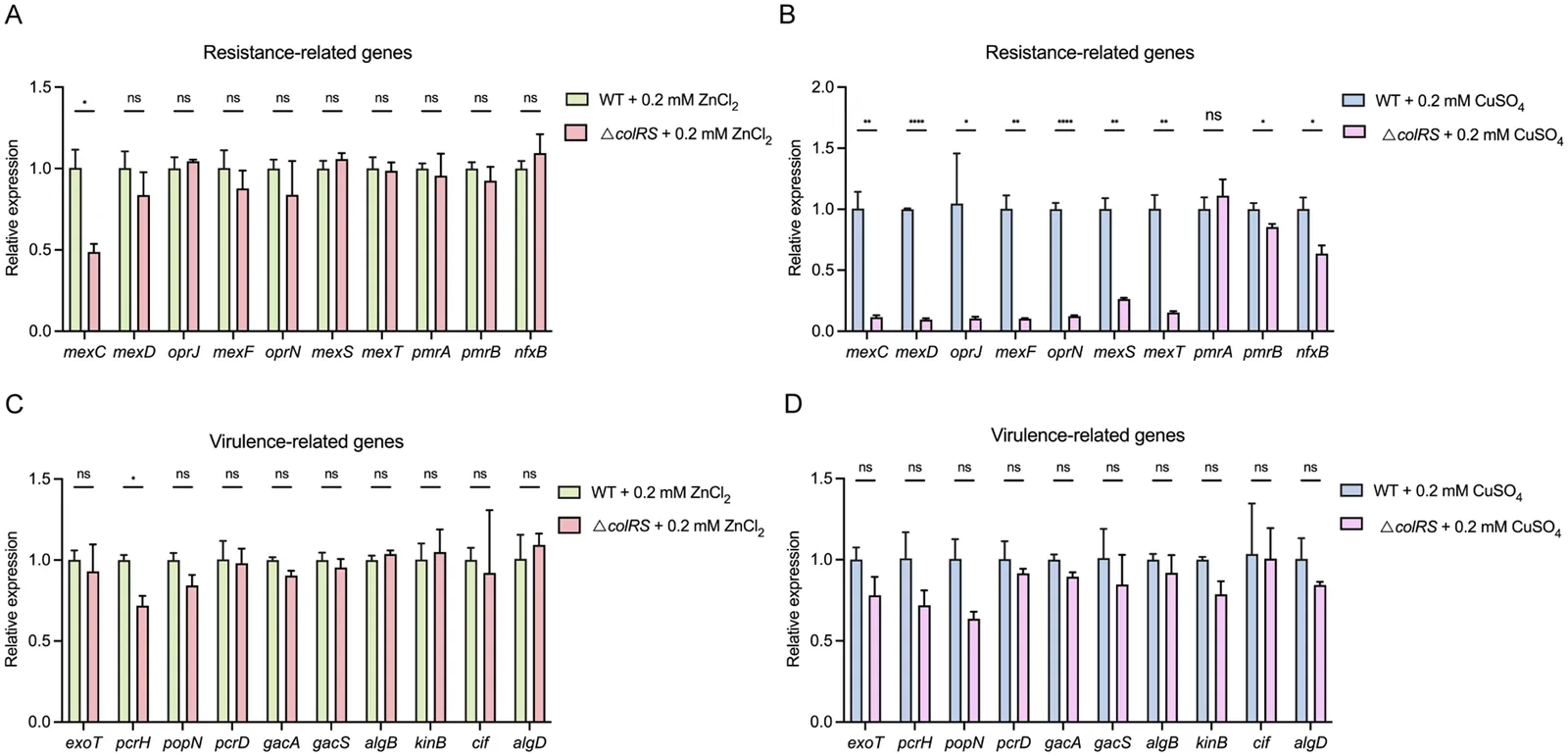
RT-qPCR analysis of resistance- and virulence- related genes expression in WT and Δ*colRS* strains under different metal stimulation conditions. (A) Expression of resistance-related genes in WT and Δ*colRS* supplemented with 0.2 mM Zn^2+^ cultured in M9 medium. (B) Expression of resistance-related genes in WT and Δ*colRS* supplemented with 0.2 mM Cu^2+^ cultured in M9 medium. (C) Expression of virulence-related genes in WT and Δ*colRS* supplemented with 0.2 mM Zn^2+^ cultured in M9 medium. (D) Expression of virulence-related genes in WT and Δ*colRS* supplemented with 0.2 mM Cu^2+^ cultured in M9 medium. The experiment was performed at least three times, and representative images from one experiment are shown. The statistical comparisons were performed using Student's *t* test. *P < 0.05, ***P < 0.001, ****P < 0.0001, ns, not significant.

To further validate the proteomic findings, we first performed RT-qPCR analyses of genes encoding representative proteins under metal stress conditions that were matched as closely as possible to those used in the proteomic experiments (Figs 7 and S8). Under 0.2 mM Zn^2+^ exposure, most tested genes exhibited only limited or nonsignificant transcriptional changes, suggesting that ColRS-mediated Zn^2+^ responses were relatively weak at the transcriptional level under this condition (Fig 7A and 7C). In contrast, under 0.2 mM Cu^2+^ stress, the Δ*colRS* mutant consistently showed transcriptional repression of multiple resistance-associated genes, including the *pmrAB* system and efflux pump-related genes *mexCD-oprJ* and *mexEF-oprN* (Fig 7B and 7D). These changes were consistent with the reduced polymyxin tolerance phenotype and generally aligned with the trends observed in the proteomic analysis. Notably, broader transcriptional repression was observed only under the higher 0.5 mM Zn^2+^ condition, suggesting that some of the Zn^2+^ -associated transcriptional changes may be related to secondary stress effects induced by elevated zinc concentrations (S8A and S8C Fig), rather than representing direct ColRS-dependent regulation. In addition, under the lower 0.1 mM Cu^2+^ condition, most tested genes displayed only limited or nonsignificant transcriptional changes, indicating that ColRS-mediated Cu^2+^ responses were also relatively weak at this concentration (S8B and S8D Fig). Overall, these results demonstrate that the transcriptional outputs regulated by ColRS are both metal-specific and concentration-dependent. Moreover, the observed transcriptional changes only partially corresponded to the steady-state proteomic alterations, likely reflecting differences in post-transcriptional regulation, protein stability, and temporal response dynamics.

## Discussion

At the host-pathogen interface, *P. aeruginosa* must continuously interpret two mechanistically distinct arms of nutritional immunity metal sequestration and metal intoxication while flexibly balancing persistence- and invasion-oriented lifestyles [1, 5]. In this study, we reposition the TCS ColRS as a molecular decision hub that discriminates zinc and copper associated microenvironments and translates these chemically similar yet biologically divergent cues into distinct adaptive outputs. Rather than functioning as a single purpose “metal detoxification” module, ColRS enables pronounced phenotypic plasticity, differentially shaping motility, virulence, and envelope defense under Zn^2+^ versus Cu^2+^ conditions. A central observation supporting this framework is the opposite transcriptional tuning of *colS, colR* in response to Zn^2+^ (induction) and Cu^2+^ (repression), which correlates tightly with metal-conditioned infection outcomes and behavioral phenotypes. This finding fills an important gap in our understanding of how *P. aeruginosa* integrates host-derived metal signals into higher-order environmental decisions [5]. Mechanistically, such divergence is best explained not by signal magnitude alone, but by the combination of ion-selective sensing by ColS and the pleiotropic, hierarchical architecture of the ColR regulon, together forming a scalable regulatory layer rather than a binary stress switch [17,18].

By integrating motif-guided target prediction with EMSA and transcriptional reporter validation, we identify direct ColR engagement at promoters controlling lipid A remodeling (e.g., *arnB*, *eptA*), metal-response regulation (*czcR*), and the alginate and motility regulator Z (*amrZ*). This wiring provides a direct mechanistic route through which metal sensing is coupled to envelop architecture and clinically relevant antibiotic tolerance [19]. Consistent with this model, ColRS deficiency selectively compromises polymyxin B and colistin resistance under metal stress, in agreement with the established role of lipid A modification and envelope remodeling in resistance to last-line, membrane-targeting antibiotics. Importantly, the downstream consequences of ColRS activation are strongly metal-context dependent. Under Zn^2+^ conditions, ColRS preferentially sustains host-interaction and offensive modules, including type III secretion system associated and virulence-related genes (*pcrD, popN, popD, cif*). Loss of ColRS under zinc stress results in coordinated suppression of these outputs, providing a quantitative link between the Zn^2+^ -conditioned proteomic module and reduced intracellular survival and systemic virulence. In contrast, under Cu^2+^ conditions, ColRS control is redirected toward envelope defense and the reprogramming of motility and chemotaxis networks. The altered abundance of motility-associated proteins (e.g., FliC, PilM, CheB) in the Δ*colRS* background aligns closely with copper-conditioned motility phenotypes, underscoring that ColRS does not simply amplify metal signals but reprioritizes downstream regulatory investment according to environmental context.

At the systems level, our quantitative proteomics further resolve how distinct metal inputs are hierarchically routed through ColRS. In WT strain, Zn^2+^ induces a comparatively focused and functionally coherent proteomic remodeling, whereas Cu^2+^ elicits a broad, proteome-wide reset reflecting the fundamentally different challenges posed by these metals during host immunity. Crucially, ColRS dependency scales with this global architecture: a compact ColRS-dependent layer under Zn^2+^ versus an expanded ColRS-dependent regulon under Cu^2+^. This layered response argues that ColRS participates not only in metal detection but also in shaping the structure and weighting of downstream adaptive programs. Our structural and biophysical analyses further suggest a molecular basis for this discrimination. We identify a non-canonical ion-sensing region within the ColS periplasmic domain (E96/H105) that is essential for Cu^2+^ binding and contributes to Zn^2+^ responsiveness. While current modeling and affinity measurements do not support assignment of these residues as a single deterministic metal-binding site, they nonetheless provide a plausible structural complement to the previously annotated FEARE motif and establish a foothold for future high-resolution, in situ structural studies [17,18]. In this context, we view ColS metal sensing as an emergent property of a distributed coordination environment rather than a rigid, single-site lock-and-key mechanism an interpretation that reconciles differential affinities, partial phenotypes, and metal-specific outputs observed across assays.

**Fig 8:**
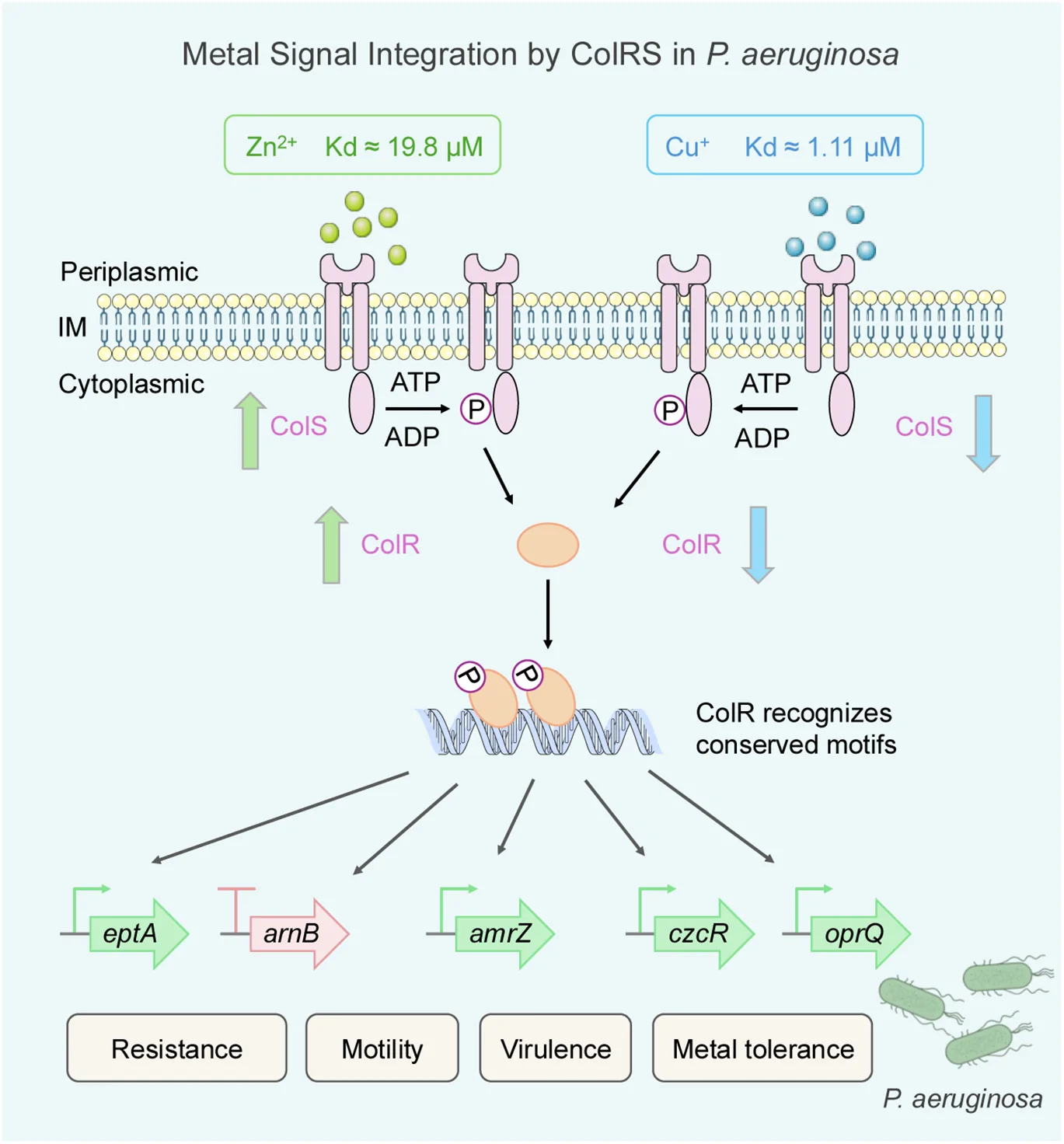
Schematic representation of ColRS metal-sensing system in regulating virulence and resistance in *P. aeruginosa.* Upon sensing zinc and copper ions, the histidine kinase ColS undergoes autophosphorylation and subsequently transfers the phosphoryl group to its cognate response regulator, ColR, yielding the activated form ColR ~ P. Activated ColR ~ P selectively associates with the promoter regions of *amrZ*, *eptA*, *arnB*, *oprQ* and *czcR*, thereby regulating their transcription. Through this regulatory cascade, the ColRS two-component system modulates key physiological traits in *P. aeruginosa*, including virulence, motility, resistance, and metal tolerance. Notably, ColS exhibits differential affinities for distinct metal ions, and ion-specific binding elicits divergent regulatory outcomes that shape the bacterial phenotype. Green arrows indicate transcriptional upregulation of *colS* and *colR* in response to Zn^2+^, whereas blue arrows indicate transcriptional downregulation in response to Cu^2+^. Dark green colored genes (*amrZ*, *eptA*, *oprQ*, and *czcR*) denote direct targets positively regulated by ColR, whereas the orange colored *arnB* gene denotes a direct target negatively regulated by ColR. The elements were provided by Servier Medical Art (https://smart.servier.com/), licensed under CC BY 4.0 (https://creativecommons.org/licenses/by/4.0/).

Beyond metal-triggered switching, our basal phenotyping and proteomics indicate that ColRS also exerts tonic regulatory activity in the absence of exogenous metal stress [14–16]. The Δ*colRS* mutant displays constitutive defects in biofilm formation and increased baseline abundance of envelope- and metal-associated factors, consistent with a model in which ColRS maintains a pre-adaptive “ready state” that metals subsequently tune allosterically rather than simply activating or repressing. This framework carries important translational implications: in inflamed or metal-perturbed host niches, metal-driven cross-resistance may bias *P. aeruginosa* toward envelope-defense states that diminish polymyxin efficacy, emphasizing the need to consider metal context when interpreting antimicrobial susceptibility and treatment outcomes. Taken together, our findings support a working model (Fig 8) in which ColRS functions as a decision-making layer that integrates metal identity with regulatory hierarchy to coordinate virulence, resistance, and physiological adaptation. By establishing ColRS as a paradigm for decoding complex host metal cues into context-appropriate survival strategies, this work lays the groundwork for future efforts to resolve ion discrimination mechanisms in vivo and to explore whether targeted disruption of ColRS signaling can sensitize multidrug-resistant *P. aeruginosa* under host-relevant metal conditions.

## Materials and methods

### Bacterial strains and growth conditions

All *P. aeruginosa* strains used in this study were derived from the PA14 WT background. Specific strains and plasmids are listed in S4 Table, and primers are provided in S5 Table. The *P. aeruginosa* PA14 WT strain was routinely cultured aerobically in Luria-Bertani (LB) medium at 37°C (220 rpm). For experiments requiring defined conditions, bacteria were grown in M9 minimal medium (50 mM Na_2_HPO_4_, 25 mM KH_2_PO_4_, 25 mM NaCl, 20 mM NH_4_Cl, 2 mM MgSO_4_, 0.1 mM CaCl_2_) supplemented with 0.4% glucose.

To evaluate metal stress responses, WT and mutant strains were grown in M9 medium supplemented with ZnCl_2_, CuSO_4_ (0–500 μM, as indicated) at 37°C for 12–24 h. For mass spectrometry (MS)-based proteomic analysis, overnight cultures were diluted to an initial OD_600_ of 0.1 in fresh M9 medium supplemented with 0.2 mM ZnCl_2_ or 0.2 mM CuSO_4_ and incubated at 37°C until mid-log phase (OD_600_ ≈ 0.6). These concentrations were selected to maintain a uniform treatment condition while minimizing severe secondary effects caused by growth inhibition, as 0.2 mM ZnCl_2_ caused only mild growth inhibition and 0.2 mM CuSO_4_ produced a more pronounced but still sublethal effect. Cells were harvested by centrifugation (3,600 rpm, 10 min, 4°C), and the resulting pellets were resuspended in MS analysis buffer (25 mM Tris-HCl, 150 mM NaCl, pH 7.5) for subsequent proteomic profiling.

### Bioinformatic analysis

Homologs of ColS and ColR were identified using DIAMOND BLAST against the Pseudomonas Genome Database and BLASTP searches against UniProt, using PA14 protein sequences as queries (coverage and sequence identity > 50%). The top hits from UniProt were selected for subsequent analysis. Multiple sequence alignment was performed using BioEdit, and phylogenetic trees were constructed using the Maximum Likelihood method in MEGA 12 [23]. Tree visualization and annotation were performed using Chiplot tvBOT [60]. For motif discovery, upstream promoter regions of ColR-regulated genes were extracted from the PA14 genome and analyzed using MEME to identify enriched conserved motifs (motif width set to 14 bp; both strands allowed).

### Construction of *P. aeruginosa* Δ*colS*, Δ*colR*, and Δ*colRS* mutants

The *colS*, *colR*, and *colRS* deletion mutants in *P. aeruginosa* PA14 were constructed using a two-step allelic exchange protocol [61]. Briefly, upstream and downstream homologous arms (~500 bp each) flanking the target genes were amplified from PA14 genomic DNA and cloned into the suicide vector pEX18Gm to generate in-frame deletion constructs. The resulting plasmids were introduced into *E. coli* S17-1 and transferred into *P. aeruginosa* PA14 via biparental mating on LB agar at 30°C for 12–16 h. Transconjugants were selected on LB containing gentamicin (50 μg/mL), followed by sucrose counter-selection on NaCl-free LB supplemented with 15% (w/v) sucrose to eliminate merodiploid. Candidate mutants were screened by colony PCR using flanking primers (S5 Table), and deletions were confirmed by sequencing of the mutated genomic regions.

### Biofilm formation assays

Biofilm formation was quantified using an adapted crystal violet (CV) staining assay [62]. Overnight cultures were diluted 1:100 in fresh LB medium, and 200 μL aliquots were transferred into each well of a sterile 96-well polyvinyl chloride (PVC) microtiter plate (BIOFIL, China). Plates were incubated statically at 37°C for 24 h. After incubation, planktonic cells were removed and wells were washed with phosphate-buffered saline (PBS, pH 7.4). Adherent biofilms were fixed with 99% methanol for 15 min, stained with 0.1% (w/v) CV for 20 min at room temperature, and destained with 33% glacial acetic acid. Absorbance was measured at 570 nm using a SuPerMax 3500 (Shanghai Flash Bio-Tech Co.). Data are presented as mean ± SD from three biologically independent experiments, each with triplicate technical replicates.

### *Galleria mellonella* infection assay

WT and mutant *P. aeruginosa* strains were cultured in M9 medium supplemented with or without 0.2 mM ZnCl_2_ or 0.2 mM CuSO_4_ to OD_600_ ≈ 0.6, harvested by centrifugation, and washed with sterile PBS. Bacterial suspensions were adjusted to 4 × 10^3^ CFU/mL, with concentrations verified by serial dilution and plating. Groups of *G. mellonella* larvae (n = 16 per strain) were injected with 10 μL of bacterial suspension using a Hamilton 50 μL microsyringe. Control larvae received an equal volume of sterile PBS. All injected larvae were maintained at 37°C and monitored at 2-h intervals over a 24-h period post-infection to assess survival rates.

### Bacterial survival in human alveolar type II epithelial cells (A549 cells)

WT and mutant *P. aeruginosa* strains were grown in M9 medium supplemented with or without 0.2 mM ZnCl_2_ or 0.2 mM CuSO_4_ to OD_600_ ≈ 0.6 and counted by serial dilution and plate counts. The A549 cells were seeded at 1 × 10^5^ cells per well in a 24-well tissue culture plate and then infected with 1 × 10^6^ cells of *P. aeruginosa* for 1 h. Sterile PBS was used as negative control. The cells were washed with PBS and incubated for another 1 h in DMEM containing gentamicin (150 μg/mL) to kill extracellular *P. aeruginosa*. Finally, the cells were washed with PBS, lysed in sterile water, and then diluted with PBS for CFU counting on LB agar plates.

### Total protein extraction assays

Total bacterial proteins were extracted using a bacterial protein extraction kit (BestBio). Extracted proteins (60 μg) from each sample were reduced by dithiothreitol in 50 mM ammonium bicarbonate buffer to a final concentration of 10 mM at 56°C for 1h. Then, samples were alkylated to block the free cysteine residues by adding 1 M iodoacetamide to a final concentration of 25 mM and incubated in darkness for additional 30 min. After that samples were precipitated by pre-cooled acetone overnight at -30°C. The protein was digested by adding 1 μg of sequence grade trypsin (Promega) at 37°C and kept for 16 h. All eluted peptide solutions were pooled and added with 10% trifluoroacetic acid to a final 0.4% v/v concentration. 10 μg peptides for each sample was desalted and dried in the speed vacuum (Christ).

### Mass spectrometry analysis

The desalted peptides were resuspended in buffer A (0.1% FA). Equal quantity of iRT peptides (Bignosys) were added to each peptide sample to correct the retention time (RT) of peptides. All peptide samples were separated on a homemade capillary column (75 μm i.d. × 25 cm, ReproSil-Pur C18-AQ, 1.9 μM; Dr. Maisch) and measured via LC-MS/MS using EASY-nLC 1200 system coupled to an Orbitrap Exploris 480 mass spectrometer (Thermo Fisher Scientific). The column temperature was set at 55°C. The mobile phase was composed of buffer A (0.1% formic acid) and buffer B (80% acetonitrile, 0.1% formic acid), and eluted at 300 nL/min with a gradient as follows: 0–2 min, 3%-8% buffer B; 2–54 min, 8–28% buffer B; 54–68 min, 28%-40% buffer B, 68–70 min, 40%-100% buffer B, 70–78 min, 100% buffer B. All the peptide samples were acquired using data independent acquisition (DIA) method. In the DIA-MS, the full scan was performed between 350–1500 m/z with 60,000 resolutions. The AGC target was set to 300% and maximum injection time was 50 ms. Then 60 DIA windows scanned from 350 to 1500 m/z with a resolution of 15,000 where precursor ions were fragmented with NCE set at 30% and analyzed with AGC target of 500% and auto maximum injection time.

The DIA raw data for samples were processed using Spectronaut 19.1.240724.62635 (Biognosys) against the directDIA analysis with UniProt *P. aeruginosa* UCBPP-PA14 proteome database (5888 entries, 2024/06/26) and the default settings. The optimal extraction window was dynamically determined by the Spectronaut based on gradient stability and iRT calibration. The retention time was predicted based on dynamic iRT. The mass tolerance for both MS1 and MS2 was set to dynamic. Identifications for peptides and proteins were filtered with a q-value cutoff of 0.01, and MS2 spectra were used for quantification. Student's *t* test was used to determine the significance of differentially expressed proteins (DEPs). Proteins with a significance level of *P* < 0.05 and a fold change of ≥ 1.5 or ≤ 0.67 were defined as DEPs.

### Protein expression and purification

The *colS* sensor domain (*colS*_SD_) and *colR* genes were amplified from *P. aeruginosa* PA14 genomic DNA and cloned into a linearized pET-22b(+) vector encoding a C-terminal 6 × His tag using the One Step Cloning Kit (Vazyme, China), yielding pET22b-ColS_SD_ and pET22b-ColR constructs. For recombinant expression, *E. coli* BL21(DE3) transformants carrying these plasmids were grown in LB containing ampicillin (50 μg/mL) at 37°C (220 rpm) to OD_600_ ≈ 0.6. Protein expression was induced with 0.4 mM IPTG for 14 h at 16°C (220 rpm). Bacteria expressing the sensory domain of ColS (ColS_SD_) and full‑length ColR were lysed by sonication in purification buffer (25 mM Tris-HCl, pH 7.5; 150 mM NaCl) and purified by Ni² ⁺ -affinity chromatography under native conditions. Purified proteins were further polished on an ÄKTA system, then concentrated, flash-frozen in liquid nitrogen, and stored at -80°C.

### Electrophoretic mobility shift assay (EMSA)

Promoter DNA fragments (100–300 bp) of target genes (*arnB*, *pap2*, *warA*, *kdpE*, *kdpD*, *kdpF*, *dgkA*, *oprQ*, *algD*, *pagL*, *eptA*, *amrZ*, *czcR*, and *czcC*) were amplified from *P. aeruginosa* PA14 genomic DNA and purified using the TIANgel Midi Purification Kit (TIANGEN). DNA-protein binding reactions (10 μL) contained 25 mM Tris-HCl (pH 7.5), 150 mM NaCl, 0.1 μM promoter DNA, and purified ColR (0-0.8 μM; two-fold serial increments). After incubation at 4°C for 30 min in the dark, 2.5 μL EMSA loading buffer (30% glycerol, 0.25% bromophenol blue) was added. Samples were resolved on 12% native polyacrylamide gels in 0.5 × TBE buffer at 4°C with buffer recirculation (130 V, 90 min). Gels were stained with ethidium bromide (0.5 μg/mL, 15 min), destained in water (10 min), and visualized using imaging system (Bio-Rad).

### β-galactosidase activity assay

Promoter regions (100–300 bp) of *eptA*, *oprQ*, *czcR*, *arnB*, and *amrZ* were amplified from *P. aeruginosa* PA14 genomic DNA and cloned upstream of the promoterless *lacZ* gene in the pRG970km vector. Reporter plasmids were co-transformed with pET22b-ColR into *E. coli* DH5α and selected on LB agar containing kanamycin (50 μg/mL) and ampicillin (50 μg/mL). Transformants were grown in LB at 37°C to OD_600_ ≈ 0.6. Cells were were pelleted (3500 rpm, 10 min, 4°C), washed, and resuspended in 1 mL Buffer Z (60 mM Na_2_HPO_4_, 40 mM NaH_2_PO_4_, 10 mM MgCl_2_, 50 mM β-mercaptoethanol, pH 7.0). Cells were permeabilized with 50 μL chloroform and 25 μL 0.1% SDS and mixed by vortexing for 15 s. Supernatant (200 μL) was mixed with 40 μL ONPG (4 mg/mL) and incubated at 25°C. Reactions were stopped with 500 μL 1 M Na_2_CO_3_. Absorbance at 420 and 550 nm was measured, and β-galactosidase activity was calculated as Miller Units: 1000 × (A_420_ -1.75 × A_550_) / (T × V × OD_600_).

### RNA preparation and RT-qPCR analysis

For RT-qPCR analysis, overnight cultures were diluted to an initial OD_600_ of 0.1 in fresh M9 medium supplemented with 0.2 mM or 0.5mM ZnCl_2_ or 0.1mM or 0.2 mM CuSO_4_ and incubated at 37°C until mid-log phase (OD_600_ ≈ 0.6). Then total RNA was extracted from bacterial cultures using TRIzol reagent (Vazyme) according to the manufacturer’s protocol. cDNA was synthesized from 1 μg total RNA using the RT reagent kit (Vazyme) with gDNA Eraser. RT-qPCR was performed in 20 μL reactions containing 2 × ChamQ SYBR qPCR Master Mix (Vazyme). RT-qPCR data were processed using the 2^-ΔΔCt^ method, with Ct values averaged from technical triplicates, normalized to a housekeeping gene (*oprL*), and reported as fold change relative to the indicated control.

### Microscale thermophoresis (MST) assays

ColS_SD_ was diluted in 1 × PBST (PBS containing 0.05% (v/v) Tween 20). His-tagged ColS_SD_ was fluorescently labeled using the Monolith His-Tag Labeling Kit RED-tris-NTA 2nd Generation (NanoTemper) following the manufacturer’s instructions. Labeled protein was adjusted to 200 nM and mixed 1:1 (v/v) with serially diluted divalent metal ions (Zn^2+^, Cu^2+^, Co^2+^, Mn^2+^, Ni^2+^, Ca^2+^, Mg^2+^ or Fe^2+^) prepared as 16 two-fold dilutions starting from 2 mM. After incubation at 4°C for 15 min, samples were loaded into premium coated capillaries and analyzed on a Monolith NT.115 instrument (NanoTemper) at 25°C (20% LED power, medium MST power). Binding curves were fitted using NanoTemper Analysis software (v1.2.20).

### Minimum inhibitory concentration assays

Minimum inhibitory concentrations (MICs) were assessed by broth microdilution in sterile 96-well plates using LB or M9 medium. The following antibiotics were tested: amikacin and tobramycin (aminoglycosides); polymyxin B and colistin (polymyxins); meropenem (carbapenem β-lactam); and ciprofloxacin (fluoroquinolone) (two-fold serial dilutions from 32 to 0.5 μg/mL, unless otherwise indicated). Two-fold serial dilutions of each antibiotic were prepared (100 μL/well) and inoculated with an equal volume of bacterial suspension standardized to 5 × 10^5^ CFU/mL, yielding a final inoculum of 2.5 × 10^5^ CFU/mL per well. After static incubation at 37°C for 24 h, MICs were defined as the lowest antibiotic concentration that completely inhibited visible growth. To evaluate ion-mediated effects on antibiotic susceptibility, assays were conducted in M9 medium (0.4% glucose as carbon source) supplemented with 0.2 mM ZnCl_2_ or 0.2 mM CuSO_4_.

### Bacterial motility assay

The motility assay was carried out as previously described with minor modifications [63]. The swarming medium consisted of 0.8% nutrient broth, 0.5% glucose, and 0.5% agar. The swimming medium consisted of 1% tryptone, 0.5% NaCl, and 0.3% agar. *P. aeruginosa* cultured in M9 medium supplemented with 0.2 mM ZnCl_2_ or 0.1 mM CuSO_4_ were diluted to an OD_600_ of 0.1, and 2 μL of the diluted bacteria were center spotted on the surface of the agar plates. After the bacterial liquid was absorbed, the swimming plates were incubated at 30°C for 16 h, and the swarming plates were incubated at 37°C for 16 h. The maximum diameter of the motility zone was recorded for three replicates per strain.

### Data analytical methods

All quantitative data are presented as mean ± SD from three independent biological replicates unless otherwise stated. Survival curves from *G. mellonella* infection experiments were analyzed using the Mantel-Cox (log-rank) test. Two-group comparisons, including DEPs in proteomics and RT-qPCR mRNA expression, were evaluated using two-tailed Student’s *t*-tests assuming equal variance. In all tests, *P* < 0.05 was considered statistically significant.

### Highlights

ColRS acts as a central hub discriminating zinc starvation and copper intoxication signals.

ColR “hard-wires” metal sensing to lipid A remodeling (*arnB*, *eptA*) and metal responsive regulator (*czcR*) to virulence (*amrZ*) regulons.

A non-canonical E96-H105 interface in ColS functions as a specificity filter to metal ions recognition.

ColRS drives a phenotypic switch: sustaining virulence under Zn^2+^ vs. fortifying envelope defense under Cu^2+^.

## Supporting Information

S1 Fig ColS and ColR are highly conserved across diverse *Pseudomonas* species. (A) Clustal Omega alignment of ColS amino acid sequences from all available ColS orthologs in the Pseudomonas Genome Database. Thirteen representative strains and the consensus sequence are shown. The predicted ion-binding motif FExxE (purple asterisk) is completely conserved. The newly identified ion-binding residues E96-H105 is indicated by a blue triangle. (B) Clustal Omega alignment of ColR amino acid sequences from all available ColR orthologs in the Pseudomonas Genome Database. Thirteen representative strains and the consensus sequence are shown. The conserved phosphorylation site is indicated by a blue triangle.
(DOCX)

S2 Fig Conservation and phenotypic characterization of ColRS mutants. (A) Phylogenetic tree of ColR including 15 response regulators from *P. aeruginosa* and ESKAPE pathogens. Phylogenetic relationships were inferred using the Neighbor-Joining method, with bootstrap values shown at the nodes. Evolutionary distances were calculated using the Poisson correction method. The analysis was performed using MEGA12. (B) Sequence similarity of ColS and ColR across 731 clinical *P. aeruginosa* isolates. (C) Growth curves of WT, Δ*colS*, Δ*colR*, and Δ*colRS* strains in M9 minimal medium. (D) Pyocyanin production. (E) Pyoverdine production. (F) Growth curves in Luria-Bertani (LB) medium. (G) Swarming motility. (H) Swimming motility. Representative images from three independent experiments are shown. Statistical comparisons were performed using one-way ANOVA. *P < 0.05, **P < 0.01, ***P < 0.001, ****P < 0.0001, ns, not significant.
(DOCX)

S3 Fig Electrophoretic mobility shift assays show no direct binding of ColR to additional target promoters. (A-J) Electrophoretic mobility shift assays (EMSA) were performed to examine the binding of purified ColR protein to promoter regions of additional putative ColR-regulated genes. The tested promoters include *pagL* (A), *pap2* (B), *dgkA* (C), *czcC* (D), *kdpE* (F), *kdpD* (G), *kdpF* (H), *algD* (I), and *warA* (J). (E) Functional categorization of putative ColR-regulated genes in *P. aeruginosa*. DNA probes were incubated with increasing concentrations of ColR protein. The final DNA concentration was 0.1 μM, and ColR was titrated from 0 to 0.8 μM. No obvious mobility shift of the DNA probes was observed with increasing ColR concentration, indicating that ColR does not directly bind to these promoter regions under the conditions tested.
(DOCX)

S4 Fig Growth phenotypes of ColRS mutants under different metal ion concentrations. (A-D) Endpoint growth of the wild-type (WT), Δ*colS*, Δ*colR*, and Δ*colRS* strains was assessed in M9 medium supplemented with 0–500 μM zinc (A, C) or copper (B, D). Growth was recorded after 12 h (A, B) and 24 h (C, D) of incubation. Representative images from one of at least three independent experiments are shown.
(DOCX)

S5 Fig Metal-dependent motility alterations in ColRS mutants. (A) Representative images showing the swarming motility of the wild-type (WT) and mutant strains following treatment with 0.2 mM zinc, 0.1 mM copper. (B) Representative images showing the swimming motility of WT and mutant strains following treatment with 0.2 mM zinc, 0.1 mM copper. (C-D) Quantification of swimming motility of WT and mutant strains in the presence of 0.1 mM copper (C) or 0.2 mM zinc (D). Statistical comparisons were performed using one-way ANOVA. *P < 0.05, **P < 0.01, ***P < 0.001, ****P < 0.0001, ns, not significant.
(DOCX)

S6 Fig Binding of ColS to common cations present in host environments. (A-D) Binding affinities of the ColS sensor domain (ColS_SD_) for calcium (A), magnesium (B), iron (C), and manganese (D) were determined by microscale thermophoresis (MST). Experiments were performed at least three times. (E-F) Confidence metrics for AlphaFold3-predicted models of *P. aeruginosa* ColS in complex with Cu²⁺ (E) or Zn²⁺ (F) used in this study. Predicted aligned error (PAE) plots indicate low positional uncertainty for most residue pairs, supporting high-confidence overall structural models. (G) Size-exclusion chromatography profiles of the wild-type and mutant ColS_SD_ proteins showing similar elution peaks. (H) SDS-PAGE analysis of the purified wild-type and mutant ColS_SD_ proteins showing high purity.
(DOCX)

S7 Fig Comparison of differentially expressed proteins (DEPs) in WT and Δ*colRS* strains under zinc and copper treatment. (A) Venn diagram showing the overlap of proteins upregulated or downregulated after zinc or copper treatment in the WT strain. (B) Heatmap showing abundance changes of proteins associated with iron uptake, oxidative phosphorylation, quorum sensing, and antibiotic tolerance in WT under different metal ion treatments. (C) Venn diagram showing the overlap of proteins upregulated or downregulated after zinc or copper treatment in WT and Δ*colRS* strains.
(DOCX)

S8 Fig RT-qPCR analysis of resistance- and virulence- related genes expression in WT and Δ*colRS* strains under different metal stimulation conditions. (A) Expression of resistance-related genes in WT and Δ*colRS* supplemented with 0.5 mM Zn^2+^ cultured in M9 medium. (B) Expression of resistance-related genes in WT and Δ*colRS* supplemented with 0.1 mM Cu^2+^ cultured in M9 medium. (C) Expression of virulence-related genes in WT and Δ*colRS* supplemented with 0.5 mM Zn^2+^ cultured in M9 medium. (D) Expression of virulence-related genes in WT and Δ*colRS* supplemented with 0.1 mM Cu^2+^ cultured in M9 medium. The experiment was performed at least three times, and representative images from one experiment are shown. The statistical comparisons were performed using Student's *t* test. *P < 0.05, ***P < 0.001, ****P < 0.0001, ns, not significant.
(DOCX)

S1 Raw Images The original uncropped and unadjusted gel images. (DOCX)

S1 Table MICs of different antibiotics against WT and mutant strains in LB medium. (DOCX)

S2 Table MICs of different antibiotics against WT and mutant strains in M9 medium. (DOCX)

S3 Table MICs of different metals against WT and mutant strains in M9 medium. (DOCX)

S4 Table Summary of differentially expressed proteins (DEPs) identified by proteomic analysis. (XLSX)

S5 Table Key resources table. (DOCX)

S6 Table Primers used in the work. (DOCX)
