# Supplementary material for: The two-component system ColRS discriminates metal signals to regulate virulence and resistance in Pseudomonas aeruginosa
Source: PLoS Pathog. 2026 Aug 28;22(8):e1014538. doi: 10.1371/journal.ppat.1014538 (PMC13524251; doi:10.1371/journal.ppat.1014538)
Supplement: Supplementary file 1 [file ppat.1014538.s016.pdf]

State Key Laboratory of Biotherapy，Sichuan University
Center of Infectious Diseases, State Key Laboratory of Biotherapy
West China Hospital, Sichuan University
Chengdu 610065, China

# Response letter

Dear reviewers,

Please find enclosed our revised manuscript entitled **"The two-component system ColRS discriminates metal signals to regulate virulence and resistance in *Pseudomonas aeruginosa*"**, manuscript number PPATHOGENS-D-26-00530, by Zhao *et. al*.

We thank the reviewers for their valuable time in reviewing our manuscript and the constructive suggestions that they have provided. We have carefully taken these comments into consideration in preparing a revised version for our manuscript, which has resulted in a more thorough and clear manuscript. Please find below a point-by-point response to the reviewers with our responses in **Blue** and the reviewers' comments in ***Black***.

**Point-by-point response to Reviewer #1**

**Part I - Summary**

*This paper presents data on the P. aeruginosa ColRS two-component system, which senses and differentiates between copper and zinc signals, translating them into distinct regulatory responses. Many of the results are consistent with previous studies of ColRS in other pseudomonads (e.g., ColR-regulated genes, involvement in virulence, etc.). However, the manuscript also provides novel insights into ColS's metal-sensing ability. Namely, the data show that, in addition to the previously identified metal-sensing FREER motif, there is another metal-sensing interface in the periplasmic domain of ColS – a non-canonical E96-H105 motif – which is required for copper and zinc sensing. Therefore, the manuscript adds a new understanding of how the conserved metal-responsible ColRS system senses and discriminates between different metal signals.*

*Unfortunately, the manuscript quality is not satisfactory, as there are numerous careless errors in the data presentation and some important information is missing (see below). In addition, the Results section is overly long and intertwined with unnecessary discussion. A critical rewrite is recommended to improve clarity and conciseness.*

Response: We thank the reviewer for the careful evaluation of our manuscript and for recognizing the novelty of our findings, particularly the identification of the non-canonical E96-H105 metal-coordination motif in ColS and its role in discriminating between copper and zinc signals. We appreciate the reviewer's constructive comments regarding the overall quality and presentation of the manuscript. In response, we have thoroughly revised the manuscript to improve rigor, clarity, and completeness.

We have carefully reviewed and corrected all data presentation issues throughout the manuscript, including figures, labeling, and quantitative descriptions, to ensure accuracy and consistency. We have also added previously missing experimental details and information to improve transparency and reproducibility. In addition, the Results section has been substantially reorganized and streamlined to reduce redundancy and to remove discussion-like content that was previously embedded within it; interpretative statements have been relocated to the Discussion where appropriate. Finally, the manuscript has been thoroughly edited for clarity and conciseness, resulting in significantly improved readability and overall logical flow. We believe that these revisions have markedly improved the overall quality of the manuscript and have addressed the reviewer's concerns.

**Part II – Major Issues: Key Experiments Required for Acceptance**

*1. Lines 255-261: This discussion here is premature and is not relevant in the results section, particularly at this stage, as no experiments testing the metal-responsive effects of ColRS are presented yet. Thus, I would suggest deleting this part.*

Response: We thank the reviewer for this helpful suggestion. We have deleted the premature discussion from Lines 255-261, as recommended.

*2. Lines 285-286, Figure S3: In the metal tolerance tests, the highest concentration of zinc and copper was 200 µM. This is quite a low concentration, particularly for highly metal-tolerant Pseudomonads, and the authors did not record any differences between strains. However, what happens if metal concentrations exceed 200 µM? I would suggest determining the MICs for zinc and copper for both the wild-type and the colRS deletion strains.*

Response: We thank the reviewer for this valuable suggestion. We agree that the concentrations tested previously (up to 200 µM) were relatively low for evaluating metal tolerance in highly tolerant Pseudomonas strains, and this point has now been clarified in the revised manuscript. In response to the reviewer's comment, we repeated the growth assays using higher concentrations of zinc and copper, extending the tested range up to 500 µM. The results showed that even at these elevated concentrations, no significant differences in growth were observed among the wild-type (WT), Δ*colS*, Δ*colR*, and Δ*colRS* strains under the tested conditions (M9 medium supplemented with 0.4% glucose as the carbon source).

We further determined the minimum inhibitory concentrations (MICs) of zinc and copper for the WT, Δ*colS*, Δ*colR*, and Δ*colRS* strains. The results indicated that there were no significant differences in metal tolerance among these strains. Specifically, the MIC values were 4 mM for zinc and 1 mM for copper. These updated data have now been incorporated into the revised manuscript (Response Figure 1 and Response Table 1, see also Fig. S4 and Table S5 in the manuscript).

The corresponding description has been included in the revised manuscript (Line 287-296): "Under the conditions used in our assays, growth of the WT, Δ*colS*, Δ*colR*, and Δ*colRS* strains was markedly inhibited as the concentrations of $Zn^{2+}$ or $Cu^{2+}$ in M9 medium increased (up to 500 µM). However, no differences in growth were observed among the strains (Fig S4). Considering that this concentration range might be insufficient to reveal differences in metal tolerance, we further determined the minimum inhibitory concentrations (MICs) of $Zn^{2+}$ and $Cu^{2+}$ in M9 medium. The MIC values were identical among WT, Δ*colS*, Δ*colR*, or Δ*colRS* strains, with $Zn^{2+}$ and $Cu^{2+}$

MICs of 4 mM and 1 mM, respectively (Table S5). These results indicating that deletion of *colS*, *colR*, or the *colRS* system does not affect resistance to these metals under our experimental conditions."

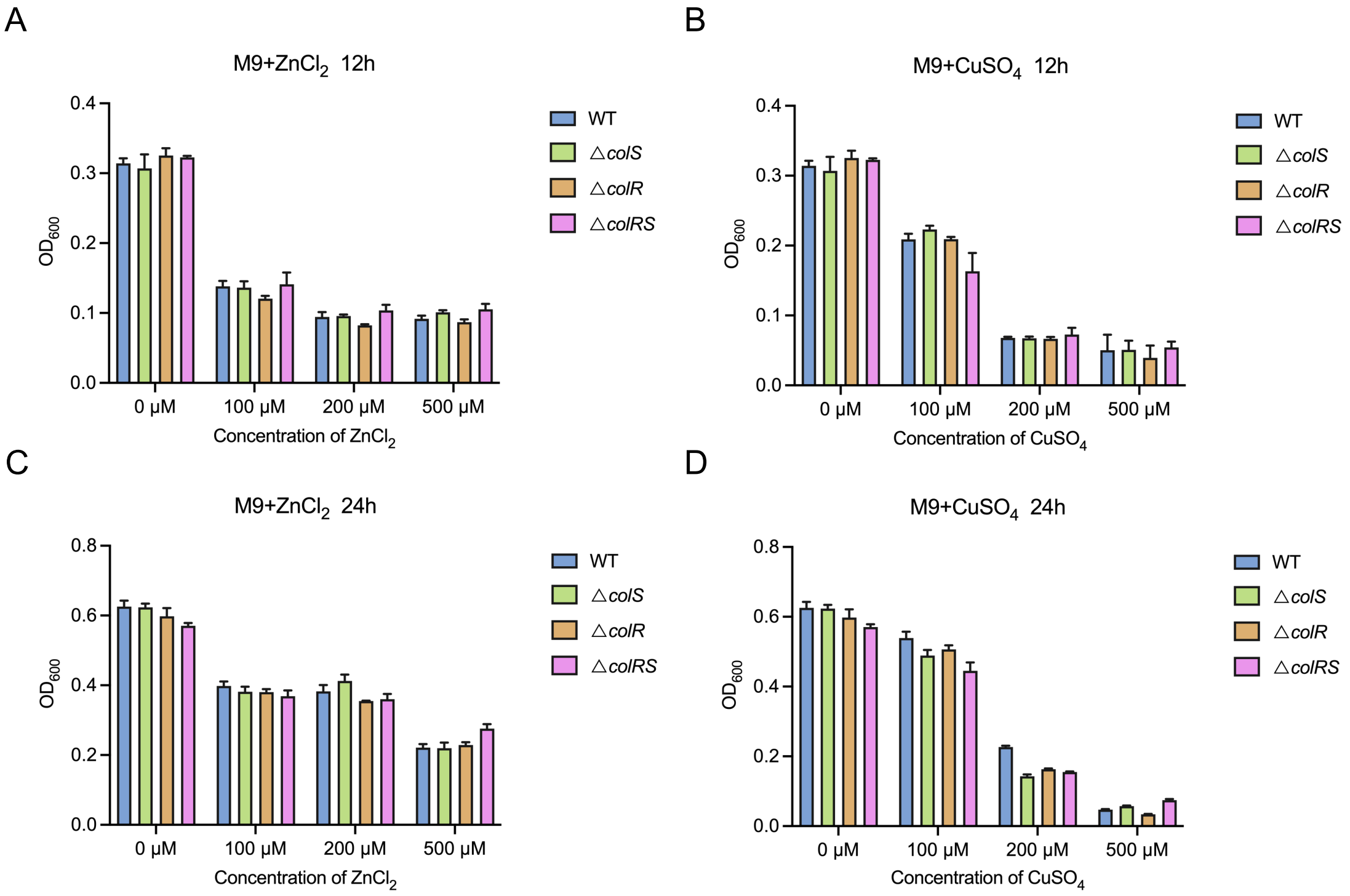


Response Figure 1. **Growth phenotypes of ColRS mutants under different metal ion concentrations.**

(A-D) Endpoint growth of the wild-type (WT), Δ*colS*, Δ*colR*, and Δ*colRS* strains was assessed in M9 medium supplemented with 0-500 μM zinc (A, C) or copper (B, D). Growth was recorded after 12 h (A, B) and 24 h (C, D) of incubation. Representative images from one of at least three independent experiments are shown.

Response Table 1. MICs of different metals against WT and mutant strains in M9 medium.

| | MIC (mM) | | | | | |
|---|---|---|---|---|---|---|
| M9 medium | $ZnCl_2$ | $CuSO_4$ | $NiSO_4$ | $CoCl_2$ | $FeSO_4$ | $MnCl_2$ |
| PA14 | 4 | 1 | 2 | 0.25 | 4 | 8 |
| Δ*colS* | 4 | 1 | 2 | 0.25 | 4 | 8 |
| Δ*colR* | 4 | 1 | 2 | 0.25 | 4 | 8 |
| Δ*colRS* | 4 | 1 | 2 | 0.25 | 4 | 8 |

*3. Lines 288-289, Figure 3A and B: Please specify (either in the text or in the figure legend) the metal concentrations that were used in the qRT-PCR experiments.*

Response: We thank the reviewer for this helpful suggestion. We have now clearly specified the metal concentrations (0.2 mM) used in the qRT-PCR experiments in both the main text and the figure legend for Figure 3A and 3B.

*4. Lines 290-292 and 307-311 and 406-413: In the Results section, I would suggest presenting results as concisely as possible. Just formulate your hypothesis or question that you intend to test or answer, and present the data obtained. Currently, following the data is hard because it is intertwined with discussion and speculation. Therefore, I would suggest moving these discussion parts to the Discussion section.*

Response: We thank the reviewer for this valuable suggestion. We have revised the indicated sections (Lines 290-292, 307-311, and 406-413) to improve clarity and conciseness. Specifically, we have streamlined the Results section to focus on the experimental data presentation; and have deleted discussion and speculative content. These interpretative elements have been relocated to the Discussion section where appropriate.

*5. Lines 311-313, Figure 3C and D: The authors claim in the text that "Under copper conditioning (0.1 mM $CuSO_4$), ColRS suppressed swarming behavior, whereas under $Zn^{2+}$ conditioning (0.2 mM $ZnCl_2$), ColRS promoted swarming (Fig. 3C, D and Fig. S4)." However, the figures show the opposite: not the ColRS system but its deficiency is causing these effects under metal exposure. Please revise the text based on the data. Also, supplement Fig. S4 legend with info about metal concentrations that were used in these motility tests.*

Response: We thank the reviewer for carefully pointing out this inconsistency. We have revised the text to accurately reflect the data shown in Figures 3C and 3D. The corrected statement now reads as follows: "Under copper ion conditions (0.1 mM $CuSO_4$), ColRS

deficiency inhibited swarming motility, whereas under zinc ion conditions (0.2 mM $ZnCl_2$), ColRS deficiency enhanced swarming motility (Figures 3C, 3D, and S5)." In addition, we have updated the legend of Figure S4 (now Figure S5 in the revised manuscript) to include the metal ion concentrations used in the motility assays (0.2 mM zinc and 0.1 mM copper).

*6. Figure 3F, G, and H: The figure legend is incomplete and contains mistakes. On panel F, the data for copper-treated, not zinc-treated, bacteria are presented. Description about data presented on panels G and H are missing. Please specify the metal concentrations used in the infection experiments. Also, for presenting the larval survival data, I would suggest using for different strains differently coloured graphs. Currently, it's hard to follow the results because all graphs are black.*

Response: We thank the reviewer for this valuable suggestion. We have carefully revised the legend for Figures 3F-H to correct the errors and ensure completeness. Specifically, we corrected the description of panel F to accurately indicate that the data correspond to copper-treated bacteria, and we added the missing descriptions for panels G and H. We have also clarified the metal concentrations used in the infection assays in the revised figure legend. In addition, following the reviewer's suggestion, we have updated the larval survival curves by using different colors to distinguish the different strains, thereby improving clarity and facilitating comparison among groups (Response Figure 2, see also Fig. 3 in the manuscript). We believe these revisions have substantially improved the accuracy and readability of the figure.

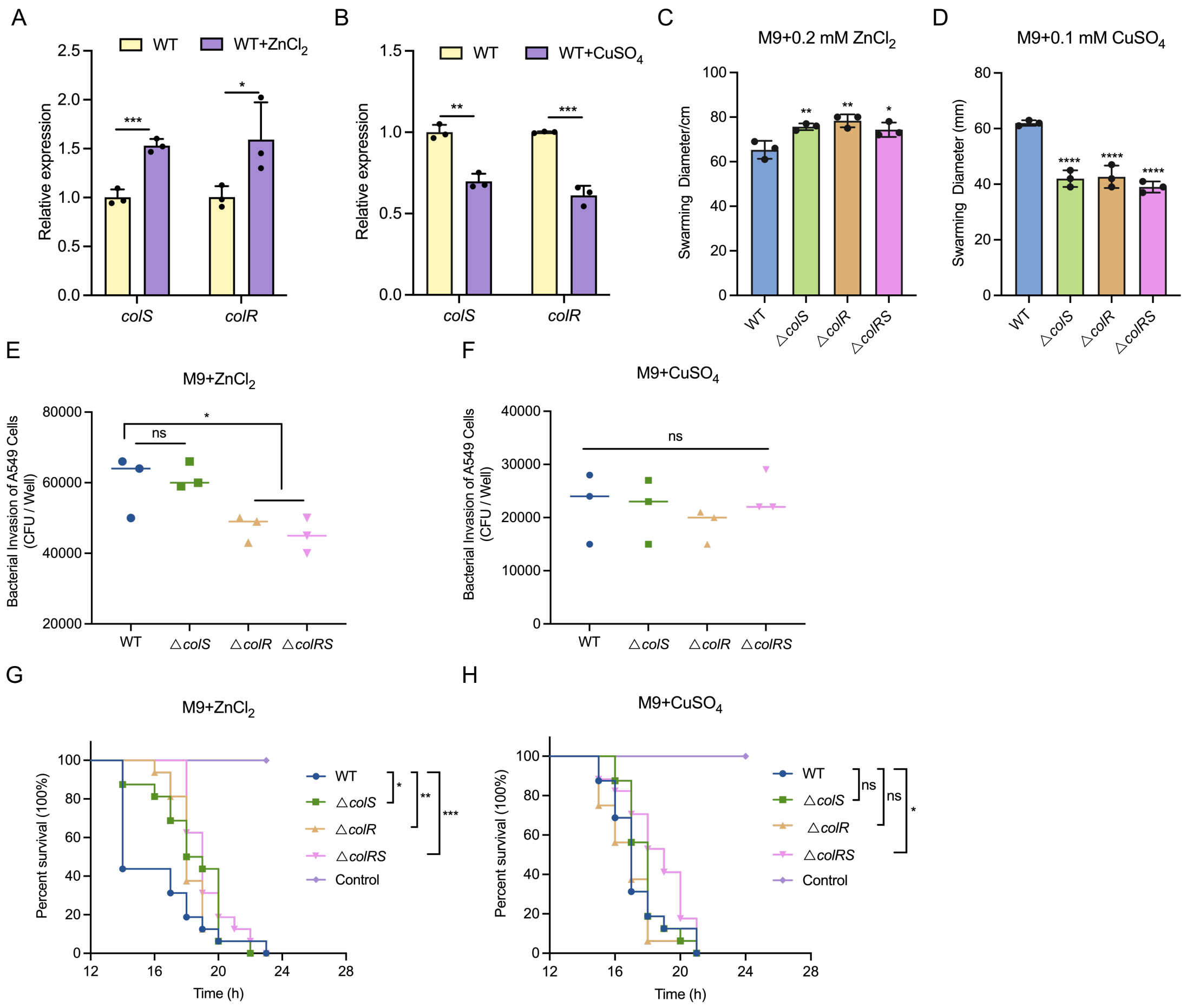


Response Figure 2. **Host-associated metals differentially tune ColRS expression and drive metal-specific phenotypic plasticity.**

(A-B) Relative expression levels of *colS* and *colR* measured by qRT-PCR following treatment with 0.2 mM zinc (A), 0.2 mM copper (B) in M9 medium. (C-D) Swarming motility of WT and Δ*colS*, Δ*colR*, Δ*colRS* mutant strains following treatment with 0.2 mM zinc (C), 0.1 mM copper (D). Data are from three independent experiments and presented as the average ± SD (error bar). (E-F) Bacterial invasion assay of WT and mutant strains following treatment with 0.2 mM zinc (E) or 0.2 mM copper (F) in A549 cells. Cells were infected with *P. aeruginosa* at a multiplicity of infection (MOI) of 10 for 1 h. (G-H) Virulence of WT and mutant strains following treatment with 0.2 mM zinc (G) or 0.2 mM copper (H) in a *G. mellonella* infection model. Larval survival was monitored for 24 h after infection. Experiments were performed at least three times, and representative results from one independent experiment are shown. Statistical comparisons were performed using one-

way ANOVA, Student's *t* test or Mantel-Cox (log-rank) test. **P* < 0.05, ***P* < 0.01, ****P* < 0.001, *****P* < 0.0001, ns, not significant.

*7. Construction of different plasmids (protein expression plasmids, β-galactosidase reporter plasmids, etc.) is not described, and none of these plasmids is presented in Table S1. Table S1 currently lists only two plasmids used for cloning (pET22b and pEX18Gm; BTW, add appropriate references to these plasmids). All the constructed plasmids should be listed and described in Table S1. Also, I suggest adding a column to Table S2 to indicate the plasmid constructed with this primer.*

Response: We thank the reviewer for this important suggestion. We have now added detailed descriptions of all constructed plasmids, including protein expression plasmids and β-galactosidase reporter plasmids, in the revised manuscript. All plasmids have been compiled and are now fully listed in Table S1, along with appropriate references. In addition, as suggested, we have updated Table S2 by adding a column indicating the plasmid associated with each primer. These revisions improve the completeness and clarity of the experimental information.

*8. Lines 366-367 and 382-383 and 398-399: During the work, the periplasmic sensor domain of ColS ($ColS_{SD}$) and its mutated variants $ColS_{SD}$-5A and $ColS_{SD}$-E96A/H105A were purified, but no data on the purified proteins are presented. However, it is important to know where all these truncated and mutated variants of ColS were equally expressed and stable. Therefore, I would suggest presenting the purification result in the Supplementary section.*

Response: We thank the reviewer for this valuable suggestion. In response, we have included the purification results of $ColS_{SD}$ and its variants ($ColS_{SD}$-5A and $ColS_{SD}$-E96A/H105A) in the Supplementary Materials (Response Figure 3, see also Fig. S6G, H in the manuscript), as recommended. These data demonstrate that the truncated and mutant proteins were purified successfully and exhibited no obvious defects in folding

or stability. We believe that the inclusion of these data improves the completeness and reliability of the study.

The corresponding description has been included in the revised manuscript (Line 370-372): "The sensor domain construct and all mutant variants displayed comparable purity and solution behavior, with similar size-exclusion chromatography elution profiles and SDS-PAGE patterns, indicating no gross defect in folding or stability (Fig. S6G, H)."

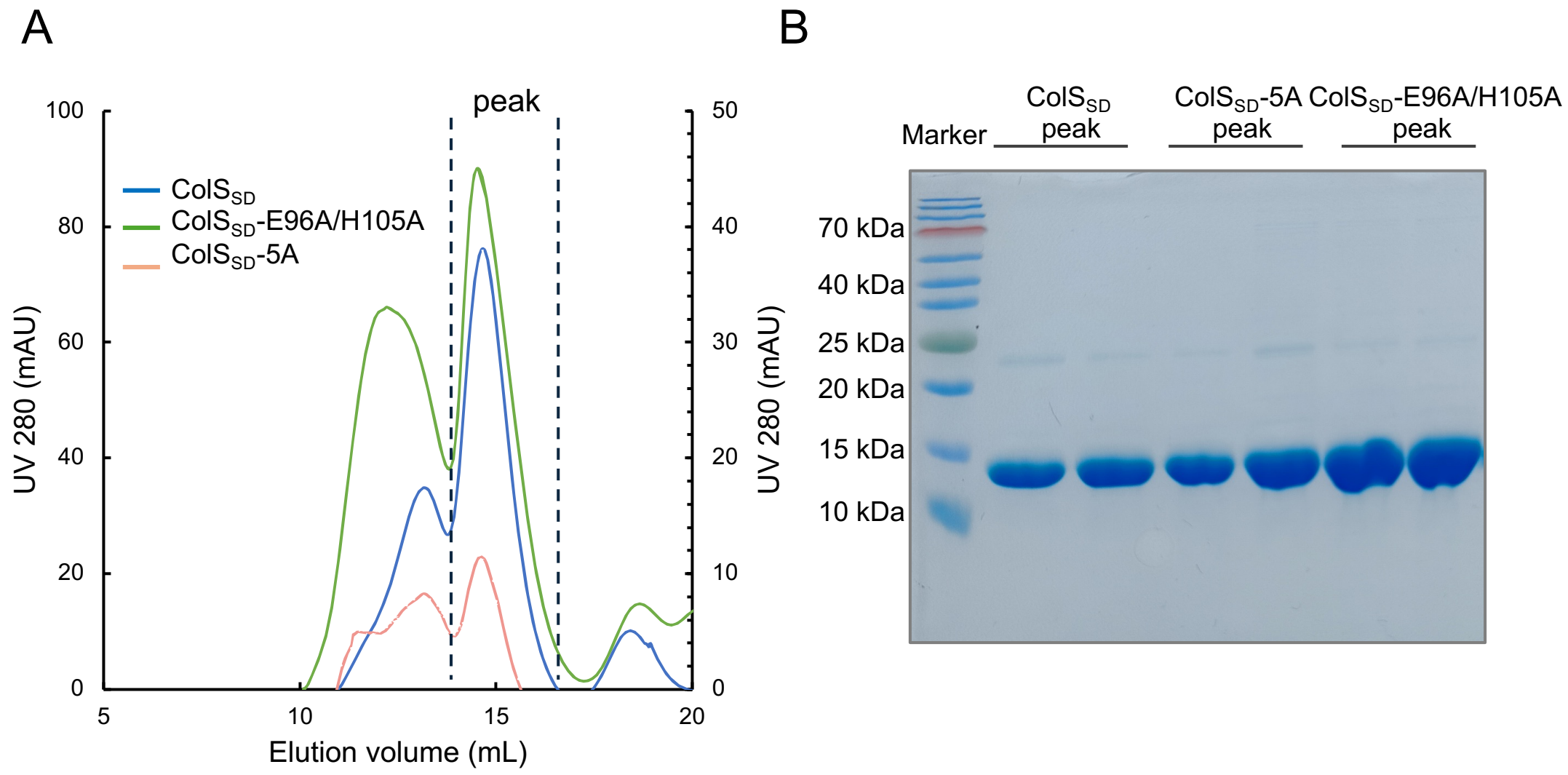


Response Figure 3. (A) Size-exclusion chromatography profiles of the wild-type and mutant $ColS_{SD}$ proteins showing similar elution peaks. (B) SDS-PAGE analysis of the purified wild-type and mutant $ColS_{SD}$ proteins showing high purity.

*9. Lines 430-431, 440-441, 455-456, and Methods: The proteome profiling experiments are not satisfactorily explained. One cannot find a method section describing how bacteria were handled during the experiment or which zinc and copper concentrations were used to trigger the proteome change. Does metal treatment affect bacterial growth rate? Given the strong proteomic response in copper-treated bacteria, it would be important to inform the reader how much adding copper affected bacterial growth. Unfortunately, no information about these issues is presented.*

Response: We thank the reviewer for this important comment. We have revised the manuscript to provide a more complete and transparent description of the proteome profiling experiments. Specifically, we have added a detailed Methods section describing bacterial culture conditions, sample preparation procedures, and the concentrations of zinc and copper used to induce proteomic changes. In addition, we have clarified the effects of metal treatment on bacterial growth to help interpret the observed proteomic responses, particularly under copper exposure. These revisions improve the clarity, reproducibility, and interpretation of the proteomics data.

The corresponding description has been included in the revised manuscript (Line 656-662): "For mass spectrometry (MS)-based proteomic analysis, overnight cultures were diluted to an initial $OD_{600}$ of 0.1 in fresh M9 medium supplemented with 0.2 mM $ZnCl_2$ or 0.2 mM $CuSO_4$ and incubated at 37 °C until mid-log phase ($OD_{600} \approx 0.6$). These concentrations were selected to maintain a uniform treatment condition while minimizing severe secondary effects caused by growth inhibition, as 0.2 mM $ZnCl_2$ caused only mild growth inhibition and 0.2 mM $CuSO_4$ produced a more pronounced but still sublethal effect."

*10. Figures 5, 6, and 7 lack information on the metal concentration that was used for bacteria treatment. Please specify which metal concentrations were used.*

Response: We thank the reviewer for this helpful suggestion. We have now specified the metal concentrations used for bacterial treatment in the legends of Figures 5, 6, and 7. These additions improve the clarity and completeness of the figure information.

*11. Lines 493-494 and Figure 7: Figure 7 presents very confusing data. Authors state they "selected representative proteins impacted by ColRS from the proteomics data, along with functionally associated neighboring genes within the same pathways or operons, for q-RT-PCR validation". It seems the selection of loci was based on copper-triggered proteome changes, as neither of those proteins was affected by zinc. Therefore, it is surprising that mRNA levels are so strongly affected by zinc. How would*

*authors explain this discrepancy between proteome data and qRT-PCR analysis? Were zinc concentrations and metal-treatment conditions (treatment time, growth media, etc.) of bacteria in proteome and qRT-PCR experiments the same or different? Again, metal concentration info is missing. Also, Figure 7 shows that all qRT-PCR-analysed loci are down-regulated in the colRS mutant, either zinc- or copper-treated bacteria were analysed. However, proteome data reveal that some of these proteins are up- and others down-regulated by copper (e.g., MexC, MexD versus PmrB, NfxB). Can authors explain this contradiction?*

Response: We thank the reviewer for the valuable comment regarding the apparent discrepancy between the proteomic and RT-qPCR datasets presented in Fig. 7. We agree that this issue required further clarification. To address this concern, we repeated the qRT-PCR experiments using different metal concentrations while carefully matching the culture conditions used in the proteomic analyses. The revised data showed that under 0.2 mM $Zn^{2+}$ exposure, most tested genes exhibited minimal transcriptional changes in the Δ*colRS* mutant, which is consistent with the relatively limited $Zn^{2+}$-associated changes observed in the proteomic dataset. In contrast, exposure to 0.2 mM $Cu^{2+}$ resulted in transcriptional repression of multiple resistance-associated genes, which was generally consistent with the proteomic and phenotypic observations (Response Figure 4, see also Fig. 7 in the manuscript).

Although the overall functional trends were similar, we acknowledge that the direction and magnitude of changes at the transcript and protein levels were not always identical for individual loci. Such discrepancies are commonly observed in bacterial stress responses and may reflect differences in post-transcriptional regulation, protein turnover, translational efficiency, and temporal dynamics between mRNA and steady-state protein abundance (Buccitelli and Selbach, 2020; Picard et al., 2013).

Notably, broader transcriptional downregulation was observed only under the higher 0.5 mM $Zn^{2+}$ condition, suggesting that these effects may reflect secondary stress responses induced by severe zinc stress rather than direct ColRS-dependent regulation. In addition, under the lower 0.1 mM $Cu^{2+}$ condition, most tested genes exhibited only

limited or nonsignificant transcriptional changes, indicating that ColRS-mediated $Cu^{2+}$ responses were also relatively weak at this concentration (Response Figure 5, see also Fig. S8 in the manuscript).

Accordingly, we replaced the original Fig. 7 with the revised qRT-PCR data obtained under 0.2 mM metal exposure conditions and clarified both the experimental conditions and the interpretation in the revised manuscript. Therefore, the revised qRT-PCR analysis should be interpreted as a targeted examination of transcriptional responses associated with ColRS-regulated pathways, rather than a direct one-to-one validation of individual protein abundance changes.

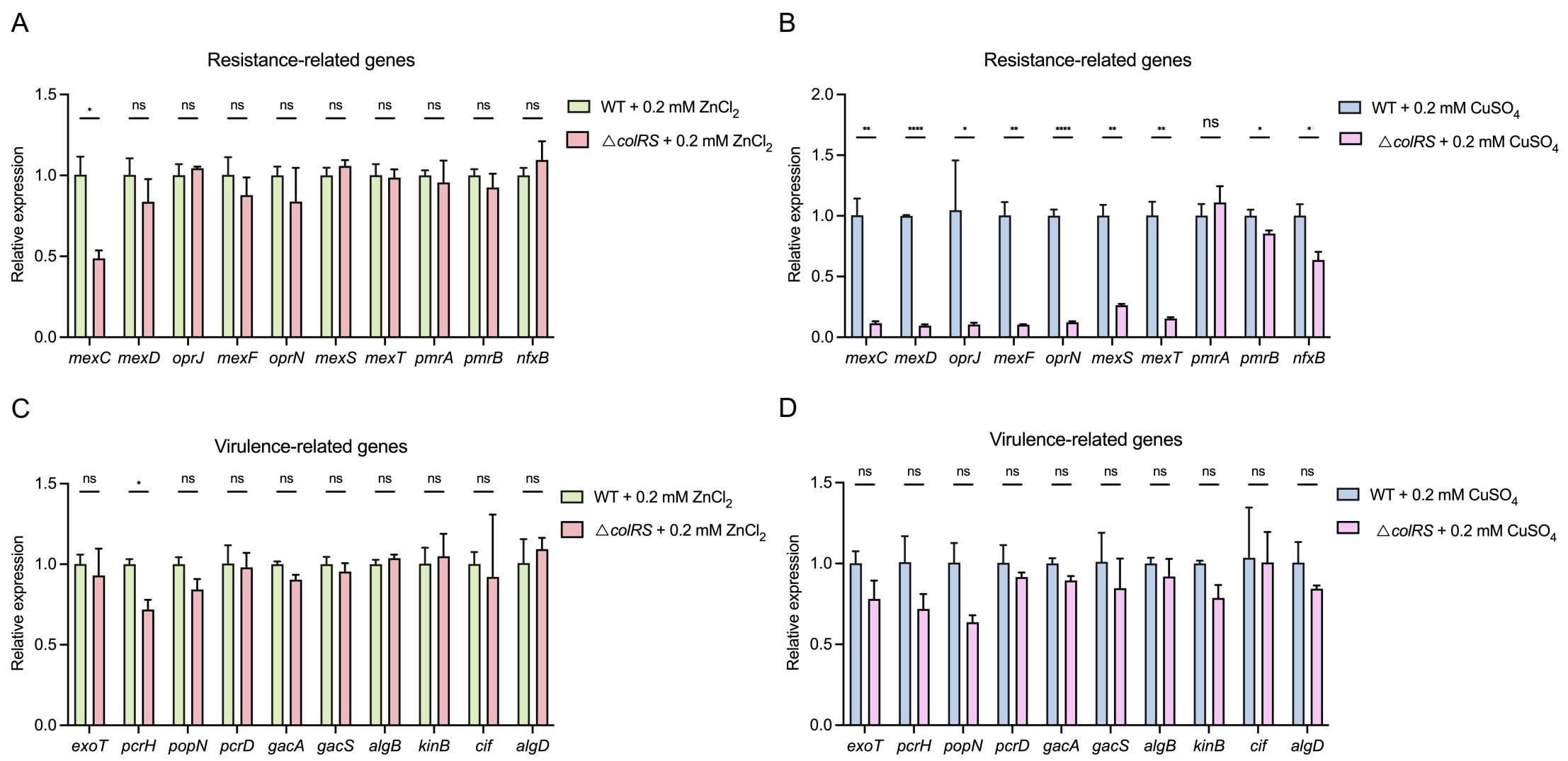


Response Figure 4. **qRT-PCR analysis of resistance- and virulence- related genes expression in WT and Δ*colRS* strains under different metal stimulation conditions.**

(A) Expression of resistance-related genes in WT and Δ*colRS* supplemented with 0.2 mM $Zn^{2+}$ cultured in M9 medium. (B) Expression of resistance-related genes in WT and Δ*colRS* supplemented with 0.2 mM $Cu^{2+}$ cultured in M9 medium. (C) Expression of virulence-related genes in WT and Δ*colRS* supplemented with 0.2 mM $Zn^{2+}$ cultured in M9 medium. (D) Expression of virulence-related genes in WT and Δ*colRS* supplemented with 0.2 mM $Cu^{2+}$ cultured in M9 medium. The experiment was performed at least three times, and representative images from one experiment are shown. The statistical comparisons were performed using Student's *t* test. **P* < 0.05, ****P* < 0.001, *****P* < 0.0001, ns, not significant.

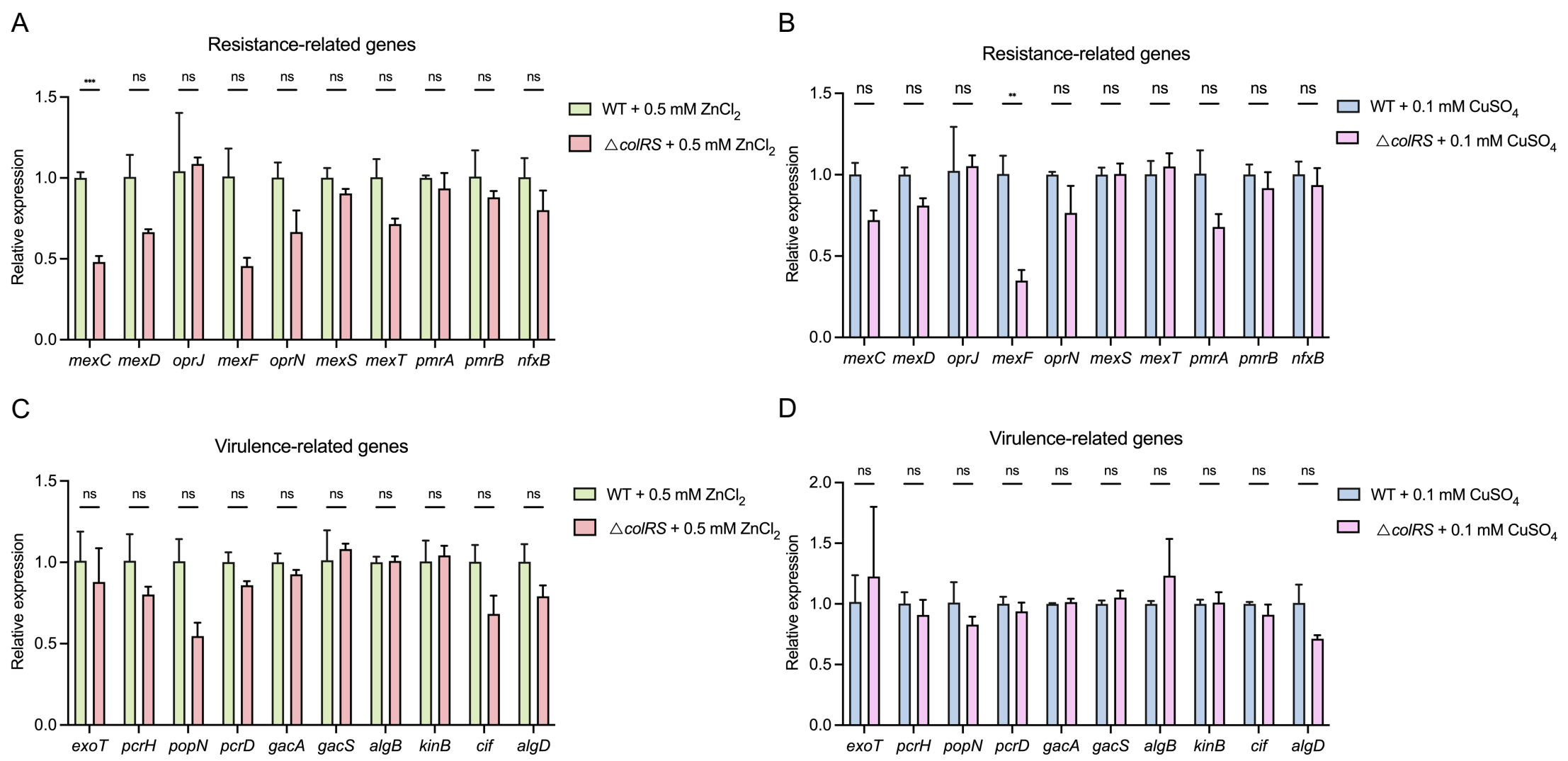


Response Figure 5. **qRT-PCR analysis of resistance- and virulence- related genes expression in WT and Δ*colRS* strains under different metal stimulation conditions.**

(A) Expression of resistance-related genes in WT and Δ*colRS* supplemented with 0.5 mM $Zn^{2+}$ cultured in M9 medium. (B) Expression of resistance-related genes in WT and Δ*colRS* supplemented with 0.1 mM $Cu^{2+}$ cultured in M9 medium. (C) Expression of virulence-related genes in WT and Δ*colRS* supplemented with 0.5 mM $Zn^{2+}$ cultured in M9 medium. (D) Expression of virulence-related genes in WT and Δ*colRS* supplemented with 0.1 mM $Cu^{2+}$ cultured in M9 medium. The experiment was performed at least three times, and representative images from one experiment are shown. The statistical comparisons were performed using Student's *t* test. **P* < 0.05, ****P* < 0.001, *****P* < 0.0001, ns, not significant.

*12. Line 499 and Figure 7: The authors state that the metal-handling genes czcD, cntM, and pcoA are suppressed in the colRS mutant, yet no data are presented in Figure 7. Either present the data or change the text.*

Response: We thank the reviewer for pointing out this issue. We have revised the manuscript accordingly and removed this description from the text to avoid unsupported interpretation and to ensure consistency between the Results section and the presented data.

*13. Lines 504-505 and Figure 7D: Data show that none of the virulence genes analysed by qRT-PCR responded to copper treatment, although their abundance was altered in the proteome. Can authors comment on this controversy between proteomic and transcriptional analysis?*

Response: We thank the reviewer for raising this important point regarding Figure 7D. We agree that the lack of obvious copper-responsive transcriptional changes detected by qRT-PCR, despite the altered abundance of virulence-associated proteins observed in the proteomic dataset, requires further clarification.

In this study, we selected several representative virulence-associated proteins identified from the ColRS-mediated copper- or zinc-responsive proteomic analyses for transcriptional validation. However, these genes are likely regulated not only by the ColRS system but also by additional regulatory pathways. Therefore, when comparing copper-treated wild-type strain with copper-treated Δ*colRS* strain at the transcriptional level, the differences under the tested conditions may not have been sufficiently pronounced to be detected by qRT-PCR.

In addition, our virulence assays consistently showed that the effect of ColRS-mediated copper signaling on virulence regulation was less pronounced than that observed under zinc-responsive conditions. This trend is consistent with the relatively modest transcriptional differences detected for these virulence-associated genes during copper treatment.

We have added clarification in the revised Discussion to explain that the discrepancy between the proteomic and transcriptional analyses likely reflects the complex and multi-layered regulation of virulence-associated factors during copper adaptation.

*14. Lines 634-644: This methodology is incomplete and needs specification of which metal concentrations were used for proteomic analyses. Since you used M9 minimal medium, you should also specify the carbon source used ("appropriate carbon source" tells nothing). If you have used different carbon sources in different experiments, then*

*you should specify this in the figure legends. The current figure legends do not indicate whether bacteria were grown in LB or M9 medium.*

Response: We thank the reviewer for this important suggestion. We have revised the Methods section to clearly specify the metal concentrations used in the proteomic analysis, as well as the carbon source in the M9 minimal medium, which was 0.4% glucose. In addition, we have updated the figure legends to indicate whether the bacteria were cultured in LB medium or M9 medium for each experiment, and, where applicable, to specify the corresponding metal concentrations. These revisions improve the clarity and reproducibility of the experimental conditions.

**Part III – Minor Issues: Editorial and Data Presentation Modifications**

*1. Lines 135-137, Figure S1: Confusing sentence. As Figure S1 presents only Clustal Omega alignment data for ColS and ColR proteins from different Pseudomonads, it remains vague how this analysis shows that "ColRS orthologs cluster into a deeply conserved clade distinct from accessory signaling loci".*

Response: We thank the reviewer for pointing out that this statement was not sufficiently clear. We have revised the sentence in lines 135-137 to more accurately reflect the analysis presented in Figure S1 and to avoid overinterpretation. The description has been further clarified to ensure consistency between the text and the data shown. The revised statement now reads: "Within the *Pseudomonas* genus, ColS and ColR orthologs display strong sequence conservation across species, as shown by Clustal Omega alignment (Fig. S1), suggesting functional conservation of the ColRS system."

*2. Line 150: Needs correction because MICs of different antibiotics are in Tables S3 and S4, not in S1.*

Response: We thank the reviewer for pointing out this error. We have corrected the text to indicate that the MIC data are presented in Tables S3 and S4, rather than Table S1.

*3. Lines 171-172: Correct "we observed up-regulated of ArnE" to "we observed up-regulation of ArnE".*

Response: We thank the reviewer for catching this error. We have corrected the sentence to "we observed up-regulation of ArnE" in the revised manuscript.

*4. Lines 177-178, and line 205: For easier reading, I would suggest adding strain where you see the change. For example, AcoB, AcoC, and AcsL were significantly up-regulated in the ΔcolRS strain.*

Response: We thank the reviewer for this helpful suggestion. We have revised the text to clearly indicate the strain context when describing gene expression changes (e.g., specifying the Δ*colRS* strain where appropriate), which improves clarity and readability.

*5. Lines 211-212: Please combine these two sentences, because there is currently unnecessary duplication.*

Response: We thank the reviewer for this suggestion. We have revised the text by combining the two sentences to eliminate redundancy and improve conciseness.

*6. Line 222: References to Mumm et al 2016 (DOI: 10.1021/acs.jproteome.6b00420) and Ainsaar et al 2014 (doi: 10.1186/1471-2180-14-162) are appropriate to add.*

Response: We thank the reviewer for this helpful suggestion. We have added the references to Mumm et al. (2016) and Ainsaar et al. (2014) in the revised manuscript Line 223.

*7. Lines 251-252: EMSA cannot reveal whether the DNA-binding protein is an activator or an inhibitor. Therefore, change "Although oprQ was clearly activated by ColR in both EMSA and reporter assays" to "Although ColR bound the oprQ promoter DNA and oprQ was activated by ColR in reporter assays".*

Response: We thank the reviewer for this important clarification. We have revised the sentence to: "Although ColR bound the *oprQ* promoter DNA and *oprQ* was activated

by ColR in reporter assays" to accurately reflect the limitations of EMSA and avoid overinterpretation.

*8. Lines 293-295: The article referred here does not claim that ColRS is related to antibiotic resistance. Thus, this reference is incorrect, and the rationale for studying antibiotic tolerance should be rephrased.*

Response: We thank the reviewer for pointing out this issue. We have revised the text to provide a more accurate rationale for investigating antibiotic tolerance and removed the incorrect implication regarding the cited reference. The revised statement now reads: "Given that ColR directly controls genes involved in lipid A modification, including *arnB* and *eptA*, which are known to influence outer membrane charge and polymyxin susceptibility [20] (Reference 20: Extracellular zinc induces phosphoethanolamine addition to *Pseudomonas aeruginosa* lipid A via the ColRS two-component system), we next assessed whether these metal signals modulate ColRS-dependent antibiotic resistance."

*9. Lines 362-363: I suggest rephrasing the title as follows: "ColS discriminates zinc and copper via a non-canonical E96-H105 motif".*

Response: We thank the reviewer for this suggestion. We have revised the title of this section to "ColS discriminates zinc and copper via a non-canonical E96-H105 motif," which more clearly reflects the key finding.

*10. Lines 448-449: Replace "we examined proteins co-regulated by $Zn^{2+}$ and $Cu^{2+}$" with "we examined proteins similarly affected by $Zn^{2+}$ and $Cu^{2+}$".*

Response: We thank the reviewer for this suggestion. We have revised the sentence to "we examined proteins similarly affected by $Zn^{2+}$ and $Cu^{2+}$" to improve clarity and accuracy.

*11. Line 460: Correct the reference to the figure. Instead of "Fig. 6A, 6B" it should be "Fig. 6A, 6C".*

Response: We thank the reviewer for identifying this error. We have corrected the figure reference to "Fig. 6A, 6C" in the revised manuscript.

*12. Lines 736-737: Replace "The sensory domain of ColS ($ColS_{SD}$) and full-length ColR were lysed by" with "Bacteria expressing the sensory domain of ColS ($ColS_{SD}$) and full-length ColR were lysed by" or "The bacteria were lysed by".*

Response: We thank the reviewer for this suggestion. We have revised the sentence to clarify that "Bacteria expressing the sensory domain of ColS ($ColS_{SD}$) and full-length ColR were lysed by", improving the accuracy of the description.

*13. Lines 767-774: Supplement this methodology with a description of how bacteria were cultivated (LB or M9 medium, etc.) and which metal concentrations were applied.*

Response: We thank the reviewer for this important comment. We have supplemented the Methods section with detailed descriptions of bacterial growth conditions, including the culture M9 medium and the metal concentrations used in each experiment. The revised statement now reads: "For qRT-PCR analysis, overnight cultures were diluted to an initial $OD_{600}$ of 0.1 in fresh M9 medium supplemented with 0.2 mM or 0.5mM $ZnCl_2$ or 0.1mM or 0.2 mM $CuSO_4$ and incubated at 37 °C until mid-log phase ($OD_{600}$ ≈ 0.6)."

*14. Line 796: Indicate which carbon source was used.*

Response: We thank the reviewer for this suggestion. We have now specified the carbon source (0.4% glucose) used in the M9 medium in the revised manuscript.

*Concerns about supplementary material*

*1. Figure S1, lines 25-26 in Supplementary, and lines 831-832 and 835-836 in the main text: For ColS and ColR Clustal Omega alignments, the authors use 10 protein*

*sequences: five from different P. aeruginosa strains and five from other Pseudomonads. Surprisingly, the authors claim that only these 10 sequences are available in the Pseudomonas Genome Database. In fact, many more ColS and ColR orthologs are reported in the Pseudomonas Database. For a more representative alignment, I would suggest aligning proteins from other species as well, e.g., from P. putida, P. entomophila, and P. chloraphis. Also, in the current alignment, please correct the names of three pseudomonads: P. avastanoi, P. tutzeri, and P. rotagens should be corrected as P. savastanoi, P. stutzeri, and P. protagens.*

Response: We thank the reviewer for this insightful suggestion. We have revised the sequence alignment to include additional representative ColS and ColR orthologs from other *Pseudomonas* species, including *P. putida*, *P. entomophila*, and *P. chloraphis*, to provide a more comprehensive analysis. We have also corrected the species names (*P. savastanoi*, *P. stutzeri*, and *P. protegens*) throughout the figure S1 (Response Figure 6).

A

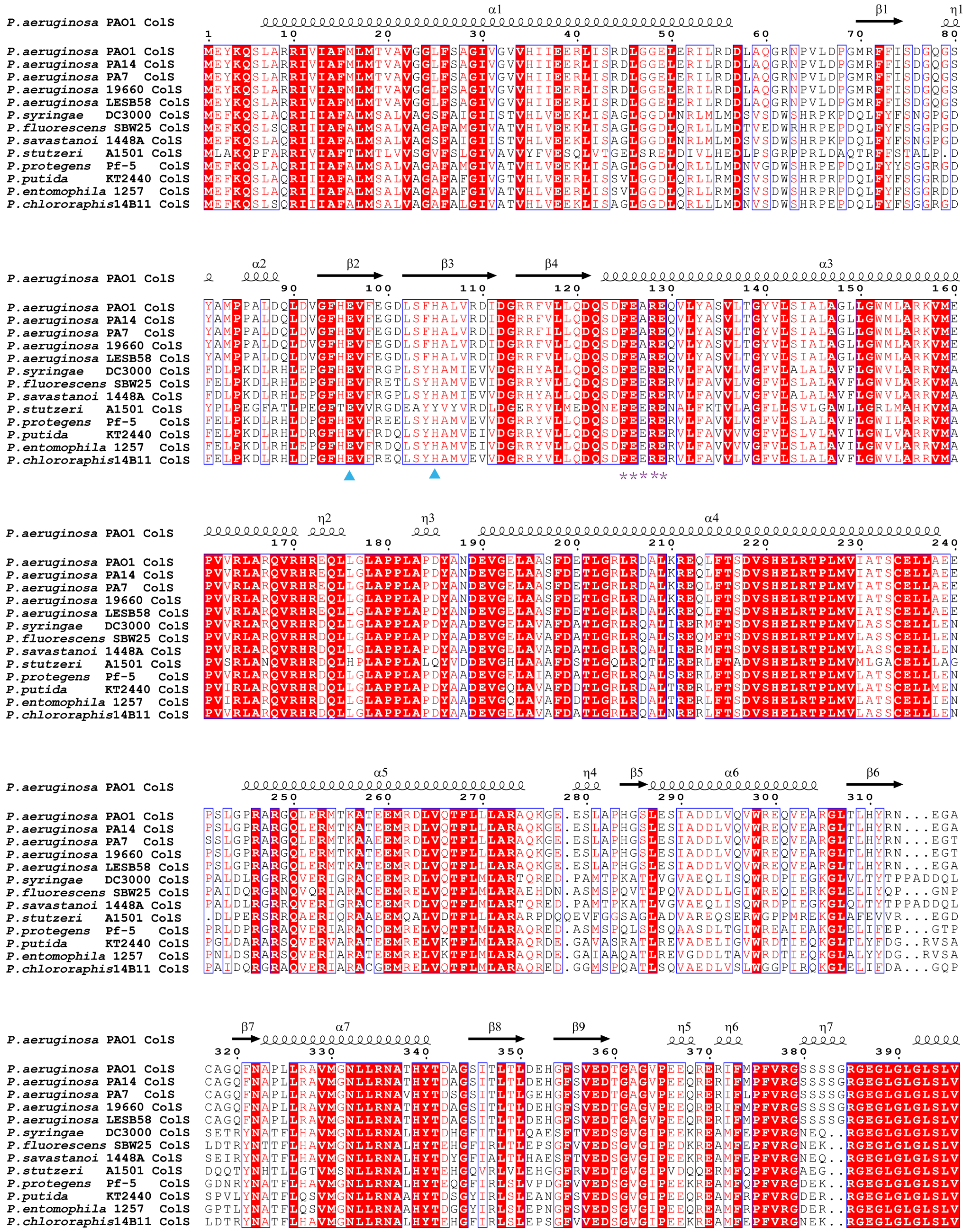
P.aeruginosa PAO1 ColS
P.aeruginosa PA14 ColS
P.aeruginosa PA7 ColS
P.aeruginosa 19660 ColS
P.aeruginosa LESB58 ColS
P.syringae DC3000 ColS
P.fluorescens SBW25 ColS
P.savastanoi 1448A ColS
P.stutzeri A1501 ColS
P.protegens Pf-5 ColS
P.putida KT2440 ColS
P.entomophila 1257 ColS
P.chlororaphis14B11 ColS

B

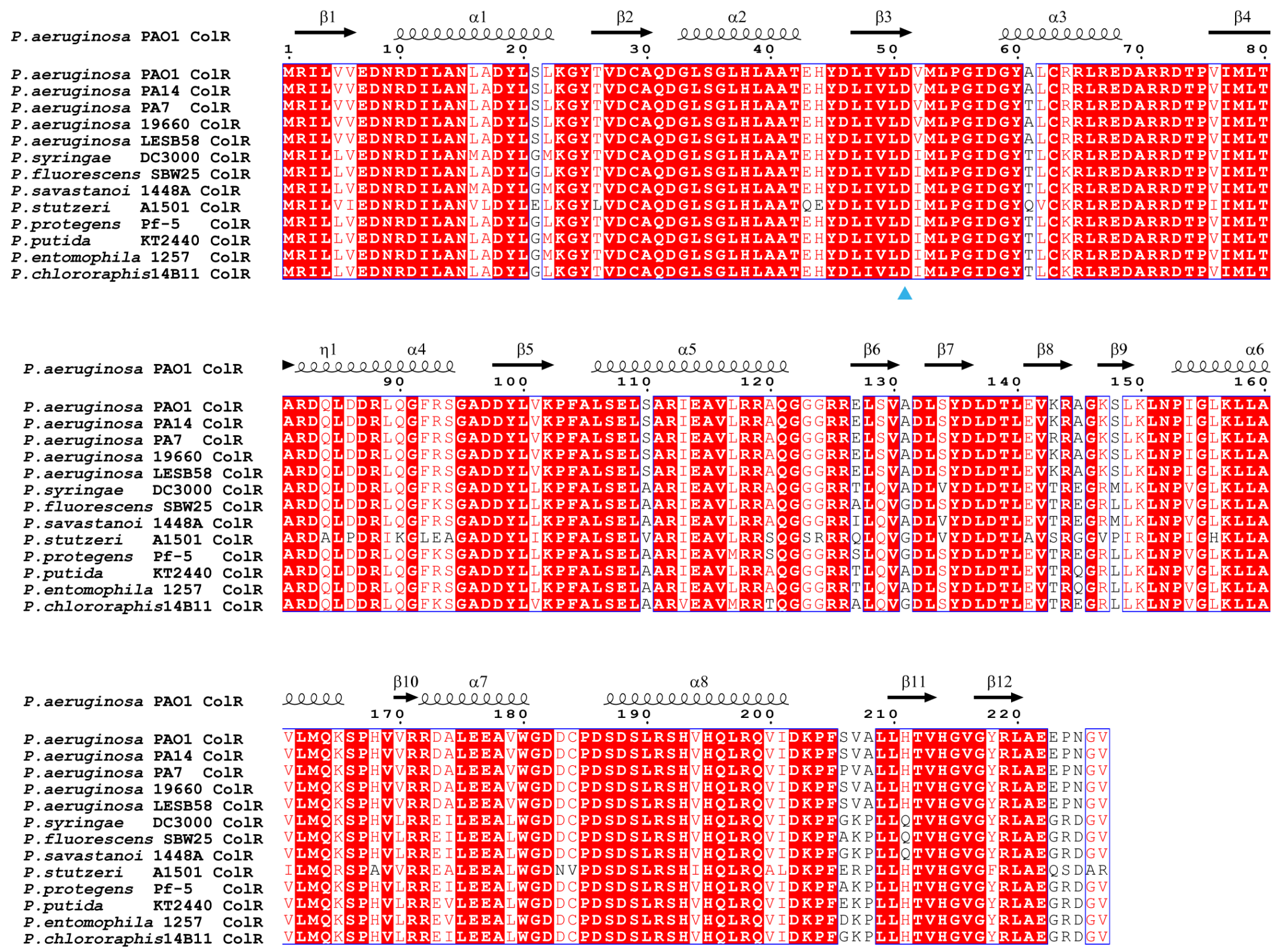

Response Figure 6. **ColS and ColR are highly conserved across diverse *Pseudomonas* species.** (A) Clustal Omega alignment of ColS amino acid sequences from all available ColS orthologs in the *Pseudomonas* Genome Database. Thirteen representative strains and the consensus sequence are shown. The predicted ion-binding motif FExxE (purple asterisk) is completely conserved. The newly identified ion-binding residues E96-H105 is indicated by a blue triangle. (B) Clustal Omega alignment of ColR amino acid sequences from all available ColR orthologs in the *Pseudomonas* Genome Database. Thirteen representative strains and the consensus sequence are shown. The conserved phosphorylation site is indicated by a blue triangle.

*2. Figure S2 panels C and F: Correct mistakes in the legend: panel C presents growth curves in M9, and panel F presents growth curves in LB medium.*

Response: We thank the reviewer for identifying these errors. We have corrected the figure legend for Figure S2 to accurately indicate that panel C shows growth curves in M9 medium and panel F shows growth curves in LB medium.

*3. Figure S3, line 53: The figure legend claims that growth curves are presented. However, no growth curves are shown in the figure. Rather, the presented bars appear to represent the endpoint measurements. Please clarify what is actually presented in the figure and correct the figure legend accordingly.*

Response: We thank the reviewer for pointing out this inconsistency. We have revised the figure legend for Figure S3 to accurately describe the data presented, clarifying that the bars represent endpoint measurements rather than growth curves.

*4. Figure S6 B: Antibiotic resistance and Virulence factor function clusters are indistinguishable, because very similar colours are used. The same concern also applies to the Quorum-sensing and Heavy-metal transport clusters. Consider changing the colours.*

Response: We thank the reviewer for this helpful suggestion. We have revised the color scheme in Figure S6B (Response Figure 7, see also Fig. S7B in the manuscript) to ensure that different functional clusters (including antibiotic resistance, virulence factors, quorum sensing, and heavy-metal transport) are clearly distinguishable. We believe these changes have improved the clarity and readability of the figure.

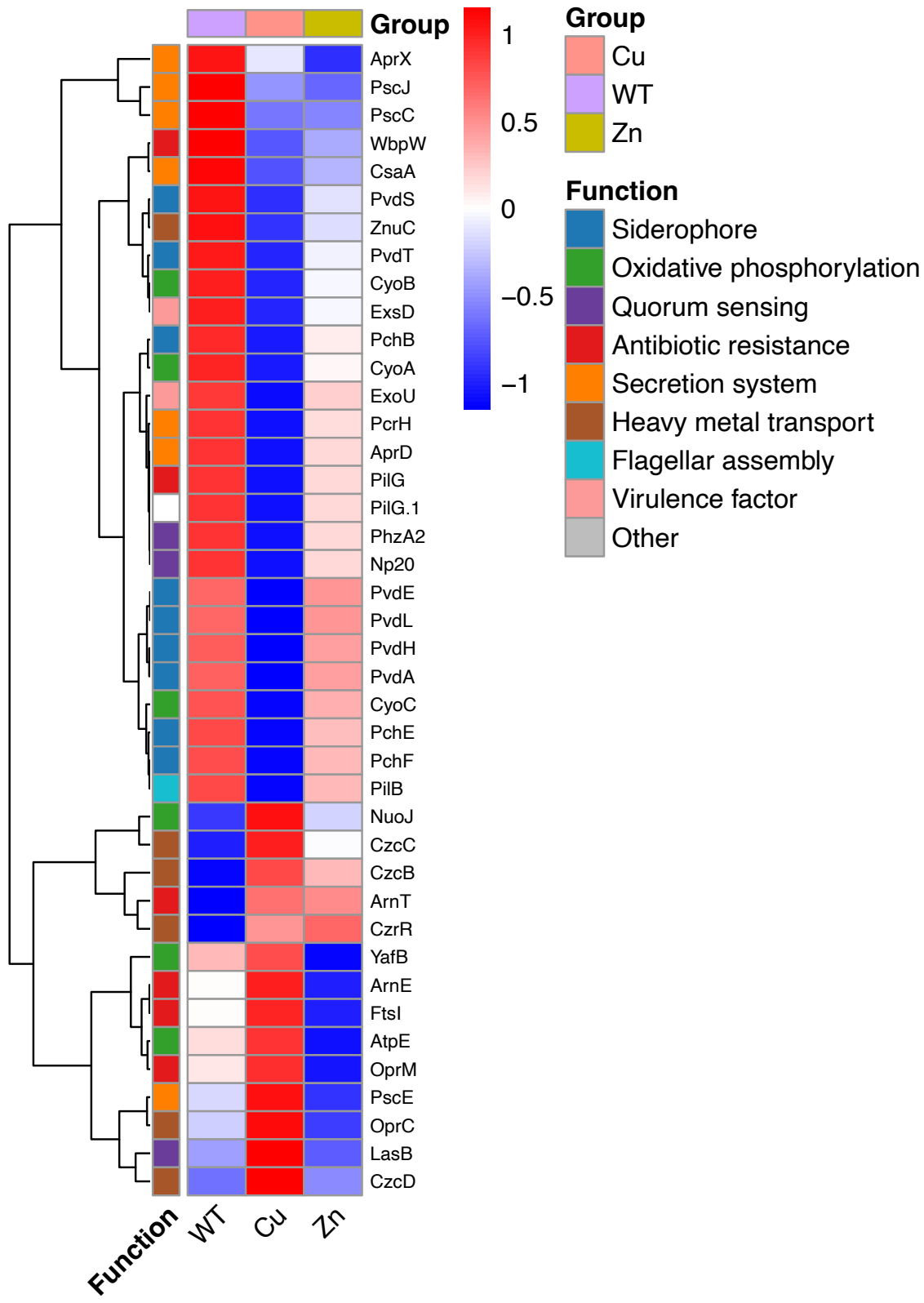


Response Figure 7. Heatmap showing abundance changes of proteins associated with iron uptake, oxidative phosphorylation, quorum sensing, and antibiotic tolerance in WT under different metal ion treatments.

## Point-by-point response to Reviewer #2

### Part I - Summary

*The authors describe a novel role for the P. aeruginosa ColRS two-component system in this manuscript. In general, it is well written and most of the data support the conclusions drawn by the authors. The manuscript uses genetics, proteomics and biophysics to identify the genes controlled by ColRS and the mechanisms used by ColRS to respond to zinc and copper. The study is quite novel as it shows the diversity of ColRS responses and shows that copper and zinc are driving the responses. The biophysics (MST) and genetics are convincing in showing a novel Zn/Cu binding site in ColS. The*

*weaknesses of the manuscript are mostly minor but there is some over interpretation of the amrZ data that needs additional clarification. Another weakness is that the EMSAs need additional controls to demonstrate ColR specificity.*

Response: We thank the reviewer for their positive and constructive assessment of our manuscript. We sincerely appreciate the reviewer's insightful comments, which have helped improve the clarity and rigor of our study. In response to the reviewer's comments, we have carefully revised the manuscript to address the concerns raised. First, we acknowledge that the previous version may have overinterpreted the data. We have now revised the relevant text and figures and moderated our conclusions regarding the role of *amrZ* to more accurately reflect the experimental evidence. In addition, we have included additional controls in the EMSA experiments to more rigorously demonstrate the specificity of ColR binding. These new data, along with their corresponding descriptions, have been incorporated into the revised manuscript. We believe that these revisions have strengthened the manuscript and adequately addressed the reviewer's concerns.

**Part II – Major Issues: Key Experiments Required for Acceptance**

*1. In Figure 8, how does activation/phosphorylation of ColR both activate and repress amrZ expression? Especially since the data are showing amrZ is activated in figure 2G but I can't find where it is repressed in the manuscript. The RT-qPCR data in P aeruginosa for amrZ show a very minor effect after deletion of both colRS.*

Response: We thank the reviewer for this important comment and for pointing out the potential inconsistency regarding *amrZ* regulation. We agree that our original presentation in Figure 8 may have caused confusion. Based on our data (Figures 2G and 2H), activation/phosphorylation of ColR leads to activation, rather than repression, of *amrZ* expression. We have therefore revised Figure 8 (Response Figure 8) accordingly to accurately reflect this regulatory relationship. Consistent with the reviewer's observation, our RT-qPCR results in *P. aeruginosa* show only a minor change in *amrZ* expression upon deletion of *colRS*, suggesting that ColRS is not a major

regulator of *amrZ* under the tested conditions, or that additional regulatory factors may be involved.

Importantly, while the effect of *colRS* deletion on *amrZ* expression is modest, we observed clear phenotypic differences in motility under different metal ion conditions. Specifically, copper treatment promotes motility, whereas zinc treatment represses motility in the *colRS* mutant background. These results suggest that ColRS contributes to motility regulation in a metal-dependent manner, potentially through pathways that are not solely dependent on *amrZ*. We have clarified these points in the revised manuscript to avoid overinterpretation and to better align our conclusions with the experimental data.

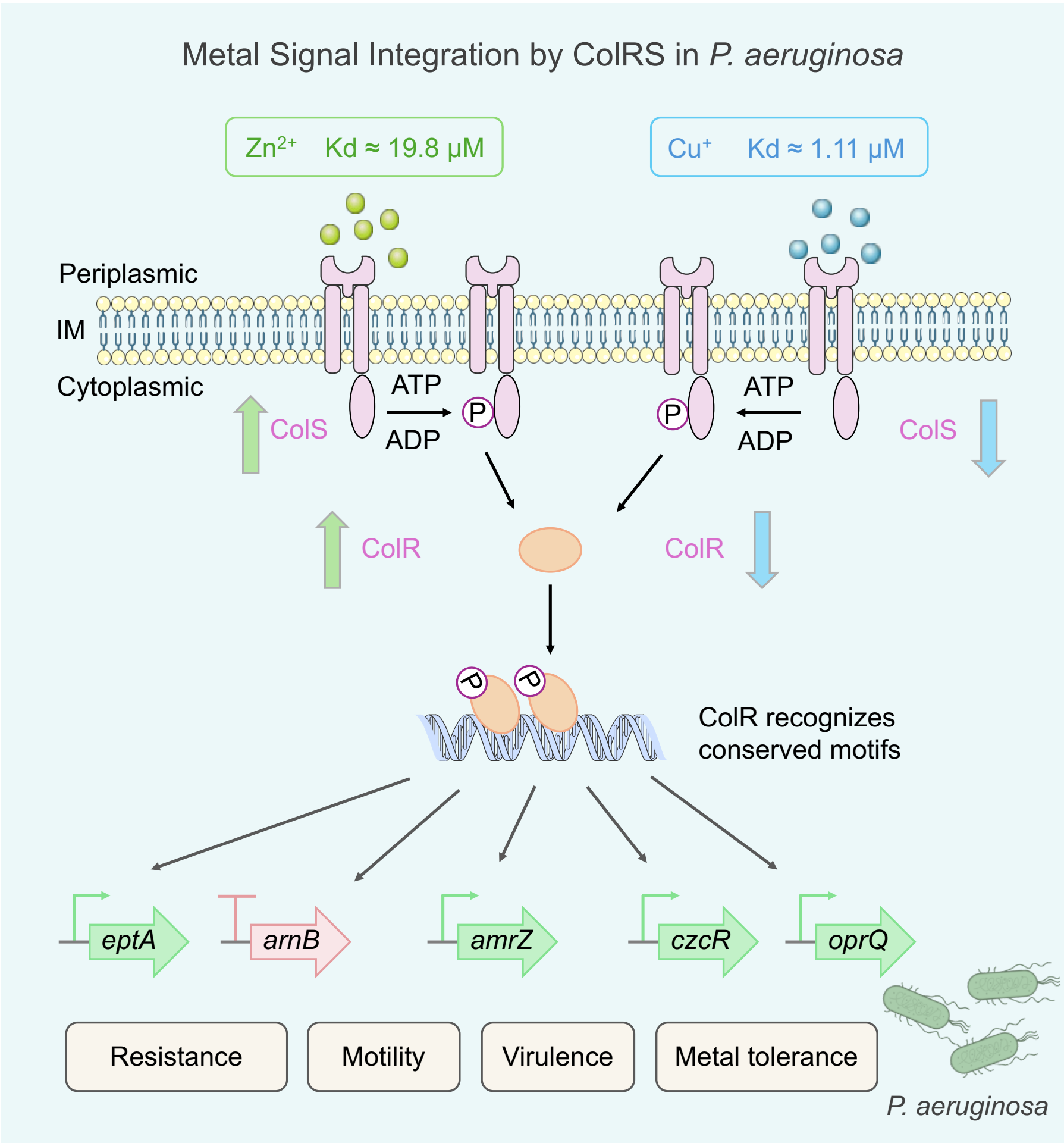


Response Figure 8. **Schematic representation of ColRS metal-sensing system in regulating virulence and resistance in *P. aeruginosa*.**

Upon sensing zinc and copper ions, the histidine kinase ColS undergoes autophosphorylation and subsequently transfers the phosphoryl group to its cognate response regulator, ColR, yielding the activated form ColR~P. Activated ColR~P selectively associates with the promoter regions of *amrZ*, *eptA*, *arnB*, *oprQ* and *czcR*, thereby promoting their transcription. Through this regulatory cascade, the ColRS two-component system modulates key physiological traits in *P. aeruginosa*, including virulence, motility, resistance, and metal tolerance. Notably, ColS exhibits differential affinities for distinct metal ions, and ion-specific binding elicits divergent regulatory outcomes that shape the bacterial phenotype.

*2. Pg. 8. For the virulence assays presented in Figure 1G and 1H. Were the bacteria grown in the presence of Zn or Cu? The material and methods sections states both conditions were used but it isn't clear which condition is being reported in this figure.*

Response: We thank the reviewer for this clarification. For the virulence assays shown in Figures 1G and 1H, the bacteria were grown in M9 medium in the absence of added Zn or Cu. We agree that this was not clearly indicated in the original version. We have now revised the figure legend to explicitly state the growth conditions used in these experiments, to avoid any ambiguity.

*3. Pg. 11. The EMSAs presented in Figure 2B,C,D,E,and F need an additional control of nonspecific DNA in the same reactions with the target promoters to show ColR specificity. Showing the ColR-binding motifs in the promoters tested would be useful information in this figure also. How conserved are the ColR binding sites within the promoters that shifted?*

Response: We thank the reviewer for this valuable suggestion. In response, we have provided additional EMSA results in the supplementary materials using the other nine nonspecific DNA fragments mentioned in the manuscript that do not interact with ColR (Response Figure 9, see also Figure S3 in the manuscript). Furthermore, we have included additional nonspecific promoter DNA fragments that cannot bind ColR as competitor controls in the EMSA reaction systems where binding was observed. The

results showed that the presence of nonspecific DNA did not affect the binding of ColR to the target promoters, thereby providing stronger evidence for the specificity of ColR binding (Response Figure 10). In addition, we have now annotated the putative ColR-binding motifs within the tested promoter regions in the figure. We also provide a description of their sequence conservation, showing that these motifs are well conserved among the promoters that exhibited a shift (Response Figure 11, see also Fig. 2A, 2B in the manuscript). These additions have been incorporated into the revised figure and corresponding text to improve the clarity and completeness of the data presentation.

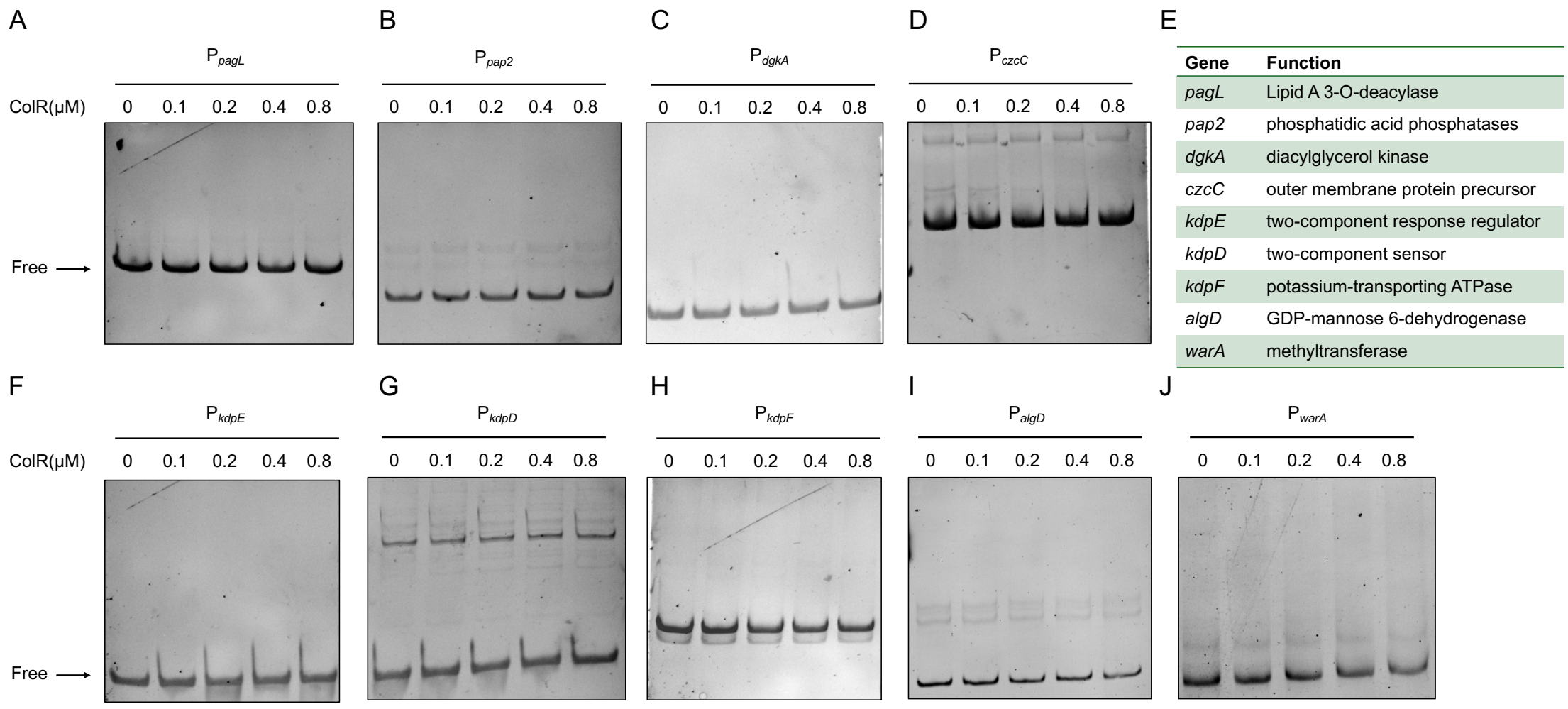


Response Figure 9. **Electrophoretic mobility shift assays show no direct binding of ColR to additional target promoters.**

(A-J) Electrophoretic mobility shift assays (EMSA) were performed to examine the binding of purified ColR protein to promoter regions of additional putative ColR-regulated genes. The tested promoters include *pagL* (A), *pap2* (B), *dgkA* (C), *czcC* (D), *kdpE* (F), *kdpD* (G), *kdpF* (H), *algD* (I), and *warA* (J). (E) Functional categorization of putative ColR-regulated genes in *P. aeruginosa*. DNA probes were incubated with increasing concentrations of ColR protein. The final DNA concentration was 0.1 μM, and ColR was titrated from 0 to 0.8 μM. No obvious mobility shift of the DNA probes was observed with increasing ColR concentration, indicating that ColR does not directly bind to these promoter regions under the conditions tested.

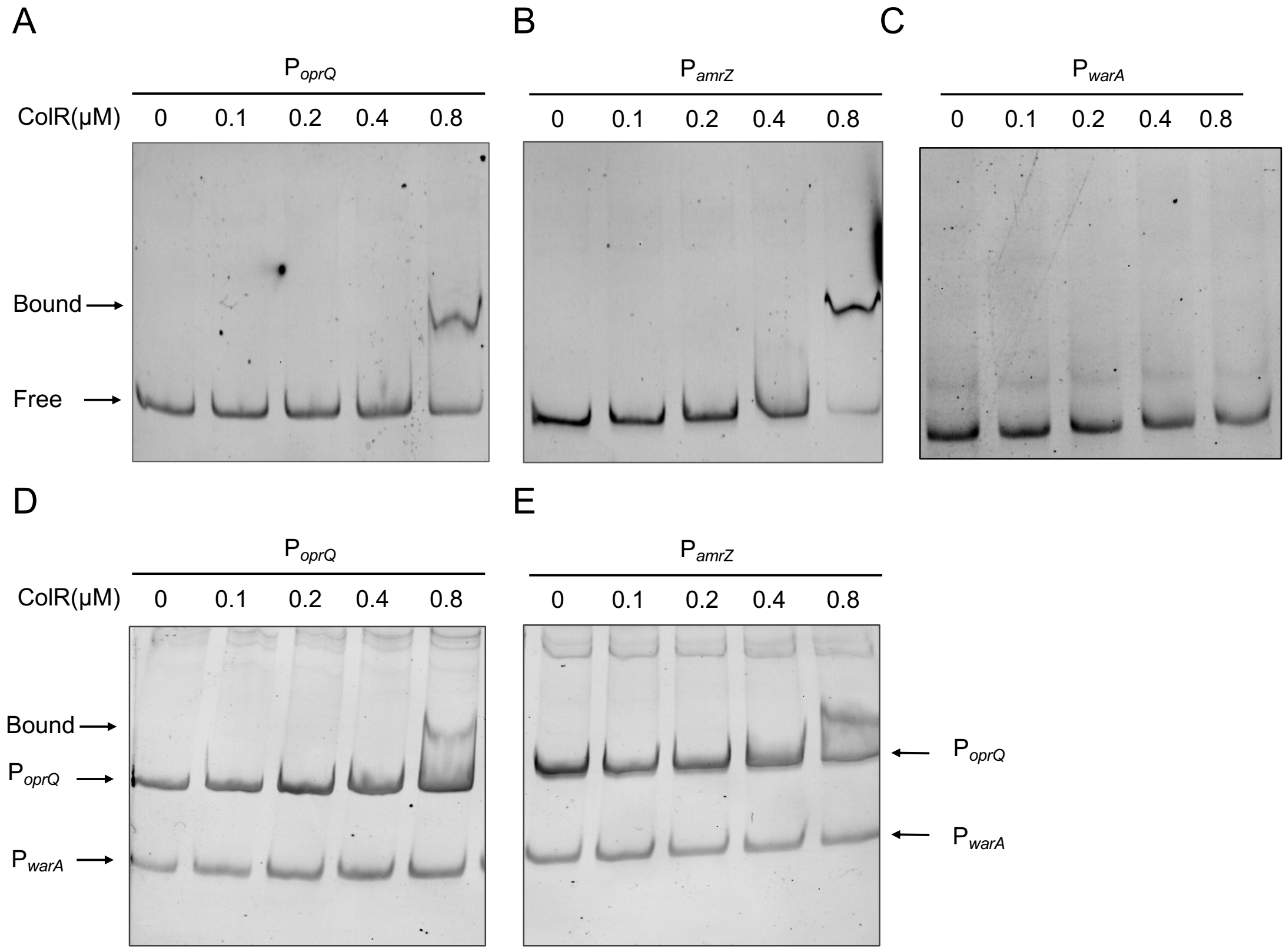


Response Figure 10. **Electrophoretic mobility shift assays reveal the binding of ColR to additional target promoters.**

(A-C) Electrophoretic mobility shift assays (EMSAs) were performed to examine the binding of purified ColR protein to the promoter regions of other putative ColR-regulated genes. The promoters tested included *oprQ* (A), *amrZ* (B), and *warA* (C). (D-E) Nonspecific *warA* promoter DNA fragments that cannot bind ColR were added to the same reaction systems as competitor controls. The presence of nonspecific *warA* DNA did not affect the binding of ColR to the specific target DNA fragments. The final DNA concentration was 0.1 μM, while the concentration of ColR was titrated from 0 to 0.8 μM.

A

| Gene | Function | Strand | Start | End | Promoter sequence |
|---|---|---|---|---|---|
| *oprQ* | outer membrane porin | + | 3119835 | 3119848 | CCGGCAACGCCCGA |
| *amrZ* | alginate and motility regulator Z | + | 3791207 | 3791220 | CACGCAACGCACAA |
| *eptA* | lipid A phosphoethanolamine transferase | - | 2153532 | 2153545 | CTGGCAGCACTCGA |
| *czcR* | two-component response regulator | + | 2843734 | 2843747 | CAGGCAACGCCCGA |
| *arnB* | β-L-Ara4N-lipid A biosynthetic | + | 3979664 | 3979677 | CAGAAAACACCCGA |

B

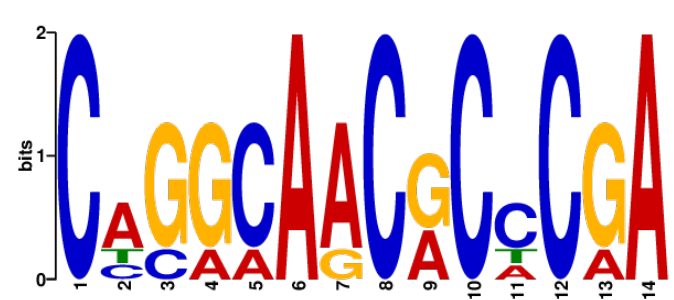

Response Figure 11. **ColR directly regulates a key regulon linking resistance and virulence.**

(A) Functional categorization of genes directly regulated by ColR in *P. aeruginosa*. The ColR-binding motifs identified in the tested promoters are indicated. (B) Conservation analysis of ColR-binding promoter motifs.

*4. There are three genes associated with alginate production reported here, amrZ, kinB and algB. Did the authors measure algD expression or alginate production under different zinc and copper conditions?*

Response: We thank the reviewer for this valuable suggestion. In response, we examined *algD* expression in both WT and Δ*colRS* strains under zinc and copper treatment conditions. However, no significant differences in *algD* transcription were observed between the two strains under either condition (Response Figure 4 and Figure 5, see also Fig. 7 and Fig. S8 in the manuscript). These results suggest that ColRS may not directly or strongly influence *algD* transcription under the tested conditions. These findings have now been incorporated into the revised manuscript.

**Part III – Minor Issues: Editorial and Data Presentation Modifications**

*1. Pg.1, lines 17-19. This sentence reads as if all of the P. aeruginosa Two-component systems control metals. Not all of the P. aeruginosa two-component systems distinguish between metals. This sentence needs additional clarification to correctly reflect the biology of P. aeruginosa Two-component systems.*

Response: We thank the reviewer for this important clarification. We agree that the original sentence could be misleading. We have revised the text to more accurately indicate that not all two-component systems (TCSs) in *Pseudomonas aeruginosa* are involved in metal sensing or discrimination, and that only specific systems respond to particular metal signals. The revised sentence now reads: "Two-component systems (TCSs) in *Pseudomonas aeruginosa* are essential for sensing and responding to diverse environmental cues, including those associated with ion homeostasis and virulence within the host. However, how specific TCSs discriminate between chemically similar metal signals to fine-tune adaptation remains unclear."

State Key Laboratory of Biotherapy，Sichuan University
Center of Infectious Diseases, State Key Laboratory of Biotherapy
West China Hospital, Sichuan University
Chengdu 610065, China

*2. Pgs 5 and 6. Either Figure 1 should be rearranged such that Figures 1D and 1E are presented in the order of the text or the text should be rearranged to reflect the order of the panels in Figure 1. It's a bit difficult to follow as written.*

Response: Thank you for this helpful suggestion. We agree that the previous arrangement may have been confusing. We have now revised Figure 1 (Response Figure 12) so that Figures 1D and 1E follow the order in which they are discussed in the text, improving consistency and readability.

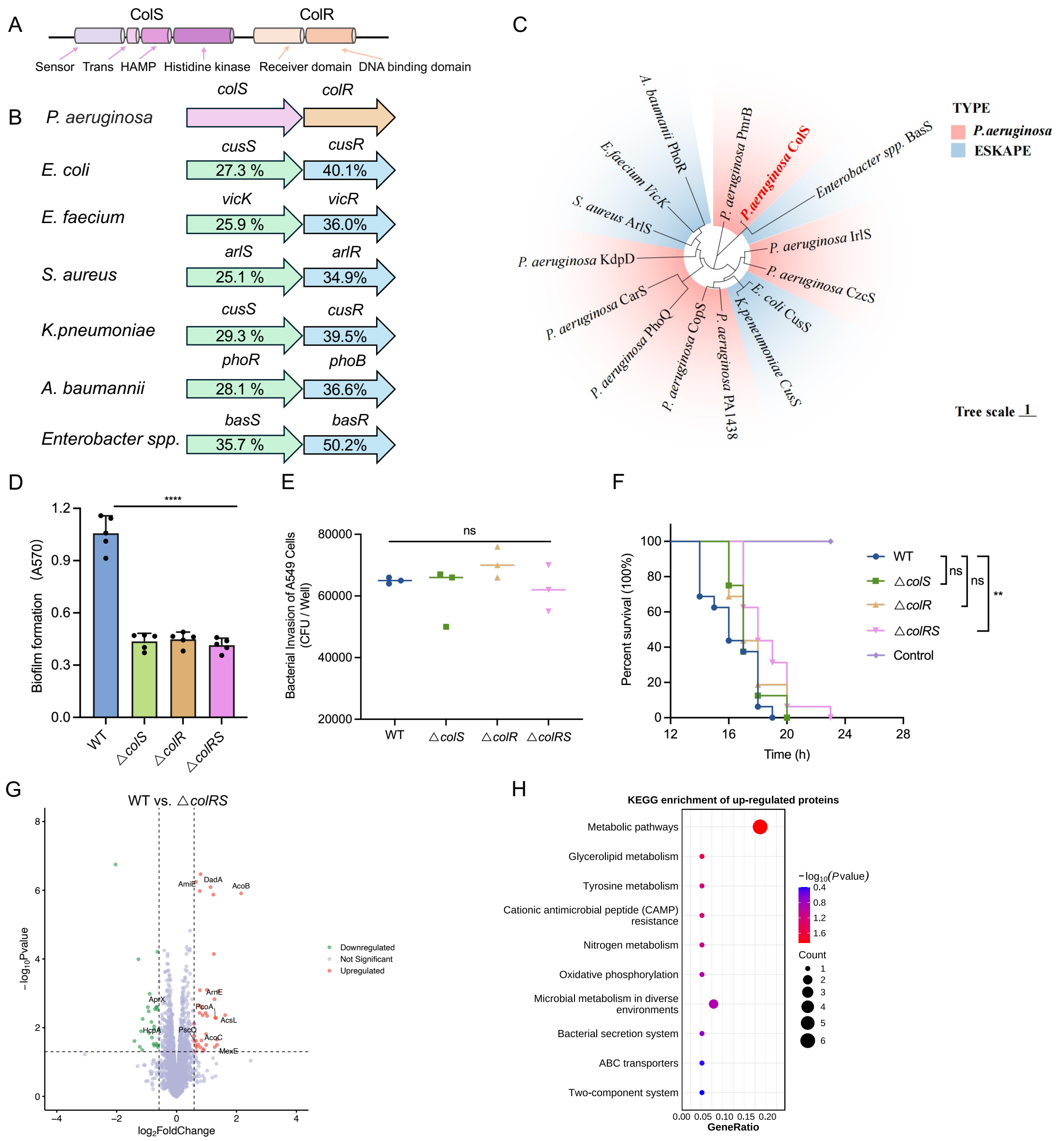

Response Figure 12. **The conserved ColRS two-component system governs basal pathogenicity and biofilm formation in *P. aeruginosa*.**

(A) Schematic of the ColRS two-component system proteins. ColS consists of sensor, transmembrane (Trans), HAMP, and histidine kinase domains. ColR comprises a receiver domain and a DNA-binding domain. (B) Schematic representation of the ColRS two-component system in *P. aeruginosa* and homologs in other ESKAPE pathogens. (C) Phylogenetic tree of ColS including 15 histidine kinases from *P. aeruginosa* and ESKAPE pathogens. Phylogenetic relationships were inferred using the Neighbor-Joining method, with bootstrap values shown at the nodes. Evolutionary distances were calculated using the Poisson correction method. The analysis was performed using MEGA12[23]. (D) Biofilm formation in WT and mutant strains in LB medium. (E) Invasion of A549 cells by WT and mutant strains. The cells were infected with *P. aeruginosa* at a multiplicity of infection (MOI) of 10 for 1 h. Bacteria were cultured in M9 medium without $Zn^{2+}$ or $Cu^{2+}$ supplementation prior to infection. (F) Virulence of WT and mutant strains in the *G. mellonella* infection model. Bacteria were cultured in M9 medium without $Zn^{2+}$ or $Cu^{2+}$ supplementation prior to infection. The survival rates of larvae were monitored for 24 h after the infection. The experiments were performed three times. (G) Volcano plot displaying the proteomic profiles of WT and Δ*colRS* strains. The significantly up and down-regulated proteins are labeled with red and green, respectively. (H) The up-regulated proteins were categorized by KEGG enrichment functional category. The statistical comparisons were performed using one-way ANOVA or Mantel-Cox (log-rank) test. *$P < 0.05$, **$P < 0.01$, ***$P < 0.001$, ****$P < 0.0001$, ns, not significant.

*3. Pg. 6, line 138. ">98.1% amino-acid" should be ">98.1% predicted amino-acid".*

Response: Thank you for pointing out this issue. We have corrected the text accordingly by changing ">98.1% amino-acid" to ">98.1% predicted amino-acid" in the revised manuscript.

*4. Pg.6, line 139. What does "intense purifying selection" mean? This should be reworded.*

Response: We thank the reviewer for this comment. We have revised the text to clarify

the meaning of "intense purifying selection" and replaced the original wording with a clearer and more precise expression. The revised sentence now reads: "The high degree of sequence conservation suggests that …".

*5. In figure 1G and 1H, There is an "M9" above the graphs. What does that mean? The bacteria were cultured in M9?*

Response: Thank you for this question. The label "M9" in Figures 1G and 1H originally indicated that the bacteria were cultured in M9 medium. To improve clarity, we have removed this label from the figure and instead included the culture conditions in the figure legend in the revised manuscript.

*6. Pg. 7, line 181. Should "in the mutant" be "in the double mutant" here?*

Response: Thank you for pointing this out. Yes, the correct wording should be "in the double mutant." We have revised the text accordingly in the manuscript.

*7. Pg 13, lines 311-313. The data in the figures 3Cand 3D are showing the opposite of what is written in the text here.*

Response: We thank the reviewer for carefully identifying this inconsistency between the text and the data shown in Figures 3C and 3D. We have corrected the statement in the revised manuscript to accurately reflect the experimental results. The revised sentence now reads: "Under copper conditioning (0.1 mM $CuSO_4$), ColRS deficiency suppressed swarming behavior, whereas under $Zn^{2+}$ conditioning (0.2 mM $ZnCl_2$), ColRS deficiency promoted swarming (Fig. 3C, D and Fig. S5)."

*8. Pg. 21, line 494. "representative proteins" should be "genes encoding representative proteins" as one doesn't perform RT-qPCR on proteins.*

Response: We thank the reviewer for pointing out this error. We agree that RT-qPCR measures transcript levels rather than proteins. Accordingly, we have revised the text

to replace “representative proteins” with “genes encoding representative proteins” in the revised manuscript.

We are genuinely grateful for your meticulous review and the opportunity to enhance the manuscript's quality. Your feedback has significantly contributed to refining our work, and we are confident that the revised manuscript effectively addresses your suggestions.

After careful checking and editing, we hope this manuscript will finally meet the standard of ***PLOS Pathogens.*** We thank again for editor and reviews’ very careful review of our manuscript. Thank you very much for all your time and efforts.

Sincerely Yours,

Rui Bao, Ph.D
Center of Infectious Diseases, Division of Infectious Diseases in State Key Laboratory of Biotherapy, West China Hospital, Sichuan University, Chengdu, China
Tel: +86-13540013347
Fax: +86-28-85164060
Email: baorui@scu.edu.cn
