# Supplementary material for: The two-component system ColRS discriminates metal signals to regulate virulence and resistance in Pseudomonas aeruginosa
Source: PLoS Pathog. 2026 Aug 28;22(8):e1014538. doi: 10.1371/journal.ppat.1014538 (PMC13524251; doi:10.1371/journal.ppat.1014538)
Supplement: Supplementary file 2 [file ppat.1014538.s001.docx]

**S1 Fig. ColS and ColR are highly conserved across diverse *Pseudomonas* species.**

(A) Clustal Omega alignment of ColS amino acid sequences from all available ColS orthologs in the *Pseudomonas* Genome Database. Thirteen representative strains and the consensus sequence are shown. The predicted ion-binding motif FExxE (purple asterisk) is completely conserved. The newly identified ion-binding residues E96-H105 is indicated by a blue triangle. (B) Clustal Omega alignment of ColR amino acid sequences from all available ColR orthologs in the *Pseudomonas* Genome Database. Thirteen representative strains and the consensus sequence are shown. The conserved phosphorylation site is indicated by a blue triangle.
