# Supplementary material for: The two-component system ColRS discriminates metal signals to regulate virulence and resistance in Pseudomonas aeruginosa
Source: PLoS Pathog. 2026 Aug 28;22(8):e1014538. doi: 10.1371/journal.ppat.1014538 (PMC13524251; doi:10.1371/journal.ppat.1014538)
Supplement: Supplementary file 3 [file ppat.1014538.s002.docx]

**S2 Fig. Conservation and phenotypic characterization of ColRS mutants.**

(A) Phylogenetic tree of ColR including 15 response regulators from *P. aeruginosa* and ESKAPE pathogens. Phylogenetic relationships were inferred using the Neighbor-Joining method, with bootstrap values shown at the nodes. Evolutionary distances were calculated using the Poisson correction method. The analysis was performed using MEGA12. (B) Sequence similarity of ColS and ColR across 731 clinical *P. aeruginosa* isolates. (C) Growth curves of WT, Δ*colS*, Δ*colR*, and Δ*colRS* strains in M9 minimal medium. (D) Pyocyanin production. (E) Pyoverdine production. (F) Growth curves in Luria-Bertani (LB) medium. (G) Swarming motility. (H) Swimming motility. Representative images from three independent experiments are shown. Statistical comparisons were performed using one-way ANOVA. **P* < 0.05, ***P* < 0.01, ****P* < 0.001, *****P* < 0.0001, ns, not significant.
