# Supplementary material for: The two-component system ColRS discriminates metal signals to regulate virulence and resistance in Pseudomonas aeruginosa
Source: PLoS Pathog. 2026 Aug 28;22(8):e1014538. doi: 10.1371/journal.ppat.1014538 (PMC13524251; doi:10.1371/journal.ppat.1014538)
Supplement: Supplementary file 4 [file ppat.1014538.s003.docx]

**S3 Fig. Electrophoretic mobility shift assays show no direct binding of ColR to additional target promoters.**

(A-J) Electrophoretic mobility shift assays (EMSA) were performed to examine the binding of purified ColR protein to promoter regions of additional putative ColR-regulated genes. The tested promoters include *pagL* (A), *pap2* (B), *dgkA* (C), *czcC* (D), *kdpE* (F), *kdpD* (G), *kdpF* (H), *algD* (I), and *warA* (J). (E) Functional categorization of putative ColR-regulated genes in *P. aeruginosa*. DNA probes were incubated with increasing concentrations of ColR protein. The final DNA concentration was 0.1 μM, and ColR was titrated from 0 to 0.8 μM. No obvious mobility shift of the DNA probes was observed with increasing ColR concentration, indicating that ColR does not directly bind to these promoter regions under the conditions tested.
