# Supplementary material for: The two-component system ColRS discriminates metal signals to regulate virulence and resistance in Pseudomonas aeruginosa
Source: PLoS Pathog. 2026 Aug 28;22(8):e1014538. doi: 10.1371/journal.ppat.1014538 (PMC13524251; doi:10.1371/journal.ppat.1014538)
Supplement: Supplementary file 5 [file ppat.1014538.s004.docx]

**S4 Fig. Growth phenotypes of ColRS mutants under different metal ion concentrations.**

(A-D) Endpoint growth of the wild-type (WT), Δ*colS*, Δ*colR*, and Δ*colRS* strains was assessed in M9 medium supplemented with 0-500 μM zinc (A, C) or copper (B, D). Growth was recorded after 12 h (A, B) and 24 h (C, D) of incubation. Representative images from one of at least three independent experiments are shown.
