# Supplementary material for: The two-component system ColRS discriminates metal signals to regulate virulence and resistance in Pseudomonas aeruginosa
Source: PLoS Pathog. 2026 Aug 28;22(8):e1014538. doi: 10.1371/journal.ppat.1014538 (PMC13524251; doi:10.1371/journal.ppat.1014538)
Supplement: Supplementary file 6 [file ppat.1014538.s005.docx]

**S5 Fig. Metal-dependent motility alterations in ColRS mutants.**

(A) Representative images showing the swarming motility of the wild-type (WT) and mutant strains following treatment with 0.2 mM zinc, 0.1 mM copper. (B) Representative images showing the swimming motility of WT and mutant strains following treatment with 0.2 mM zinc, 0.1 mM copper. (C-D) Quantification of swimming motility of WT and mutant strains in the presence of 0.1 mM copper (C) or 0.2 mM zinc (D). Statistical comparisons were performed using one-way ANOVA. **P* < 0.05, ***P* < 0.01, ****P* < 0.001, *****P* < 0.0001, ns, not significant.
