# Supplementary material for: The two-component system ColRS discriminates metal signals to regulate virulence and resistance in Pseudomonas aeruginosa
Source: PLoS Pathog. 2026 Aug 28;22(8):e1014538. doi: 10.1371/journal.ppat.1014538 (PMC13524251; doi:10.1371/journal.ppat.1014538)
Supplement: Supplementary file 7 [file ppat.1014538.s006.docx]

**S6 Fig. Binding of ColS to common cations present in host environments.**

(A-D) Binding affinities of the ColS sensor domain (ColS_SD_) for calcium (A), magnesium (B), iron (C), and manganese (D) were determined by microscale thermophoresis (MST). Experiments were performed at least three times. (E-F) Confidence metrics for AlphaFold3-predicted models of *P. aeruginosa* ColS in complex with Cu²⁺ (E) or Zn²⁺ (F) used in this study. Predicted aligned error (PAE) plots indicate low positional uncertainty for most residue pairs, supporting high-confidence overall structural models. (G) Size-exclusion chromatography profiles of the wild-type and mutant ColS_SD_ proteins showing similar elution peaks. (H) SDS-PAGE analysis of the purified wild-type and mutant ColS_SD_ proteins showing high purity.
