# Supplementary material for: The two-component system ColRS discriminates metal signals to regulate virulence and resistance in Pseudomonas aeruginosa
Source: PLoS Pathog. 2026 Aug 28;22(8):e1014538. doi: 10.1371/journal.ppat.1014538 (PMC13524251; doi:10.1371/journal.ppat.1014538)
Supplement: Supplementary file 8 [file ppat.1014538.s007.docx]

**S7 Fig. Comparison of differentially expressed proteins (DEPs) in WT and Δ*colRS* strains under zinc and copper treatment.**

(A) Venn diagram showing the overlap of proteins upregulated or downregulated after zinc or copper treatment in the WT strain. (B) Heatmap showing abundance changes of proteins associated with iron uptake, oxidative phosphorylation, quorum sensing, and antibiotic tolerance in WT under different metal ion treatments. (C) Venn diagram showing the overlap of proteins upregulated or downregulated after zinc or copper treatment in WT and Δ*colRS* strains.
