# Supplementary material for: The two-component system ColRS discriminates metal signals to regulate virulence and resistance in Pseudomonas aeruginosa
Source: PLoS Pathog. 2026 Aug 28;22(8):e1014538. doi: 10.1371/journal.ppat.1014538 (PMC13524251; doi:10.1371/journal.ppat.1014538)
Supplement: Supplementary file 9 [file ppat.1014538.s008.docx]

**S8 Fig. RT-qPCR analysis of resistance- and virulence- related genes expression in WT and Δ*colRS* strains under different metal stimulation conditions.**

(A) Expression of resistance-related genes in WT and Δ*colRS* supplemented with 0.5 mM Zn²⁺ cultured in M9 medium. (B) Expression of resistance-related genes in WT and Δ*colRS* supplemented with 0.1 mM Cu²⁺ cultured in M9 medium. (C) Expression of virulence-related genes in WT and Δ*colRS* supplemented with 0.5 mM Zn²⁺ cultured in M9 medium. (D) Expression of virulence-related genes in WT and Δ*colRS* supplemented with 0.1 mM Cu²⁺ cultured in M9 medium. The experiment was performed at least three times, and representative images from one experiment are shown. The statistical comparisons were performed using Student's *t* test. **P* < 0.05, ****P* < 0.001, *****P* < 0.0001, ns, not significant.
