# Supplementary material for: The two-component system ColRS discriminates metal signals to regulate virulence and resistance in Pseudomonas aeruginosa
Source: PLoS Pathog. 2026 Aug 28;22(8):e1014538. doi: 10.1371/journal.ppat.1014538 (PMC13524251; doi:10.1371/journal.ppat.1014538)
Supplement: Supplementary file 11 [file ppat.1014538.s010.docx]

# S1 Table. MICs of different antibiotics against WT and mutant strains in LB medium.

| **MIC (μg/mL)** | | | | | | |
| --- | --- | --- | --- | --- | --- | --- |
| LB medium | Amikacin | Tobramycin | Polymyxin B | Colistin | Meropenem | Ciprofloxacin |
| PA14 | 8 | 2 | 4 | 4 | 2 | 32 |
| △*colS* | 16 | 2 | 4 | 4 | 2 | 16 |
| △*colR* | 8 | 2 | 4 | 4 | 2 | 32 |
| △*colRS* | 16 | 2 | 4 | 4 | 2 | 16 |
