# Supplementary material for: The two-component system ColRS discriminates metal signals to regulate virulence and resistance in Pseudomonas aeruginosa
Source: PLoS Pathog. 2026 Aug 28;22(8):e1014538. doi: 10.1371/journal.ppat.1014538 (PMC13524251; doi:10.1371/journal.ppat.1014538)
Supplement: Supplementary file 13 [file ppat.1014538.s012.docx]

# S3 Table. MICs of different metals against WT and mutant strains in M9 medium.

| **MIC (mM)** | | | | | | |
| --- | --- | --- | --- | --- | --- | --- |
| M9 medium | ZnCl_2_ | CuSO_4_ | NiSO_4_ | CoCl_2_ | FeSO_4_ | MnCl_2_ |
| PA14 | 4 | 1 | 2 | 0.25 | 4 | 8 |
| △*colS* | 4 | 1 | 2 | 0.25 | 4 | 8 |
| △*colR* | 4 | 1 | 2 | 0.25 | 4 | 8 |
| △*colRS* | 4 | 1 | 2 | 0.25 | 4 | 8 |
