# Supplementary material for: The two-component system ColRS discriminates metal signals to regulate virulence and resistance in Pseudomonas aeruginosa
Source: PLoS Pathog. 2026 Aug 28;22(8):e1014538. doi: 10.1371/journal.ppat.1014538 (PMC13524251; doi:10.1371/journal.ppat.1014538)
Supplement: Supplementary file 14 [file ppat.1014538.s014.docx]

# S5 Table. Key resources table.

| **Reagent or Resource** | **Source** | **Identifier** |
| --- | --- | --- |
| **Bacterial cells and plasmids** |  |  |
| *E. coli* BL21(DE3) | Beijing Genesand Biotech Co.,Ltd | Cat# SEC19 |
| *E. coli* DH5α | Beijing Genesand Biotech Co.,Ltd | Cat# SCC01 |
| *E. coli* S17-1 | This study | N/A |
| *P. aeruginosa* PA14 | This study | N/A |
| *P. aeruginosa* PA14 △*colS* | This study |  |
| *P. aeruginosa* PA14 △*colR* | This study |  |
| *P. aeruginosa* PA14 △*colRS* | This study |  |
| pET22b(+) | This study | [1] |
| pET22b-ColR | This study |  |
| pET22b-ColS_SD_ | This study |  |
| pET22b-ColS_SD_-5A | This study |  |
| pET22b-ColS_SD_-E96A/H105A | This study |  |
| pRG970Km | This study | [2] |
| pRG970Km-P*_oprQ_* | This study |  |
| pRG970Km-P*_armZ_* | This study |  |
| pRG970Km-P*_eptA_* | This study |  |
| pRG970Km-P*_czcR_* | This study |  |
| pRG970Km-P*_arnB_* | This study |  |
| pEX18Gm | This study | [3] |
| pEX18Gm-△*colS* | This study |  |
| pEX18Gm-△*colR* | This study |  |
| pEX18Gm-△*colRS* | This study |  |
| **Chemicals** |  | |
| Ni-NTA agarose | ThermoFisher | Cat# R90110 |
| His-Tag Labeling Kit RED-tris-NTA 2nd generation | Beijing NanoTemper Technologies Co.,Ltd | MO-L018 |

# References

1.	Studier FW, Rosenberg AH, Dunn JJ, Dubendorff JW. Use of T7 RNA polymerase to direct expression of cloned genes. Methods Enzymol. 1990;185:60-89. Epub 1990/01/01. doi: 10.1016/0076-6879(90)85008-c. PubMed PMID: 2199796.

2.	Yan X, Yang R, Zhao RX, Han JT, Jia WJ, Li DY, et al. Transcriptional Regulator PhlH Modulates 2,4-Diacetylphloroglucinol Biosynthesis in Response to the Biosynthetic Intermediate and End Product. Appl Environ Microbiol. 2017;83(21). Epub 2017/08/20. doi: 10.1128/aem.01419-17. PubMed PMID: 28821548.

3.	Hoang TT, Karkhoff-Schweizer RR, Kutchma AJ, Schweizer HP. A broad-host-range Flp-FRT recombination system for site-specific excision of chromosomally-located DNA sequences: application for isolation of unmarked Pseudomonas aeruginosa mutants. Gene. 1998;212(1):77-86. Epub 1998/07/14. doi: 10.1016/s0378-1119(98)00130-9. PubMed PMID: 9661666.
