# Supplementary material for: The two-component system ColRS discriminates metal signals to regulate virulence and resistance in Pseudomonas aeruginosa
Source: PLoS Pathog. 2026 Aug 28;22(8):e1014538. doi: 10.1371/journal.ppat.1014538 (PMC13524251; doi:10.1371/journal.ppat.1014538)
Supplement: Supplementary file 15 [file ppat.1014538.s015.docx]

# S6 Table. Primers used in the work.

| **Purpose/Name** | **Sequence (5'-3')** | **Plasmid** |
| --- | --- | --- |
| **Protein Expression** |  |  |
| pET22b-ColS_FL_-F | TTAAGAAGGAGATATACATATGATGGAGTATAAGCAGAGCCTCGC | pET22b-ColS |
| pET22b-ColS_FL_-R | GGTGGTGGTGGTGGTGCTCGAGAGCAACATCGAGTAAAACTTCGAACC | pET22b-ColS |
| pET22b-ColS_SD_-F | TTAAGAAGGAGATATACATATGGAGGAGCGCCTGATTTCCCGCG | pET22b-ColS_SD_ |
| pET22b-ColS_SD_-R | GGTGGTGGTGGTGGTGCTCGAGCACCTGCTCGCGCGCTTCGAAA | pET22b-ColS_SD_ |
| pET22b-ColS_SD_-5A-F | GCCGCCGCCCAGGTGCTCGAGCACCACCAC | pET22b-ColS_SD_-5A |
| pET22b-ColS_SD_-5A-R | GGCGGCATCGCTCTGGTCCTGCAGCAG | pET22b-ColS_SD_-5A |
| pET22b-ColS_SD_- E96A/H105A-F | GACTTGTCGTTCGCCGCGCTGGTGCGCGACATC | pET22b-ColS_SD_-E96A/H105A |
| pET22b-ColS_SD_-E96A/H105A-R | GCCCTCGAACACGGCGTGGAAACCGACATC | pET22b-ColS_SD_-E96A/H105A |
| pET22b-ColR-F | TTAAGAAGGAGATATACATATGATGCGAATACTGGTGGTCGAAG | pET22b-ColR |
| pET22b-ColR-R | GGTGGTGGTGGTGGTGCTCGAGTACTCCATTCGGCTCCTCCGC | pET22b-ColR |
| **EMSA assay** |  |  |
| P-*armZ*-F | CGTTCTCCCTCCAATCGGTTGC |  |
| P-*armZ*-R | ACATTGAACCTGTAGAGTCAGG |  |
| P-*oprQ*-F | GTCACGTCCCTCTTTGTCGATC |  |
| P-*oprQ-*R | CTTATTGTTCCTGGTTGTTGGC |  |
| P-*czcR*-F | TGCGCCGCGGACATTTCCAAAG |  |
| P-*czcR*-R | GTTCGCCCCTATATAAAGTATG |  |
| P-*eptA*-F | GGAAAGCTTCCCGCGAACTT |  |
| P-*eptA*-R | GGGCACGGATCTTCACTGAC |  |
| P-*arnB*-F | CTCCCCATGTTTCCATGAAGTT |  |
| P-*arnB*-R | TAATGAAGCCTCGGTACTGGG |  |
| P-*pagL*-F | TGAAACGTCCTTTTCGAAAG |  |
| P-*pagL*-R | TCGTTCCTGGCAGACGTGTC |  |
| P-*pap2*-F | ACTGCATGGCGTCTTCCATGGC |  |
| P-*pap2*-R | GCGATCTCCAACGCTGGCGTTG |  |
| P-*dgkA*-F | GTCGGGTTCTCATCGATTCG |  |
| P-*dgkA-*R | GAGACGGCTTAACGCTTTTTTG |  |
| P-*czcC*-F | ATTGTCCGTGGAGAATATTC |  |
| P-*czcC*-R | GTTCCGCTCCTCGTCTGCTG |  |
| P-*kdpE*-F | GCCCGGCGCACTTGCCTGGC |  |
| P-*kdpE-*R | GGCACTCGGCACGGCAGGTG |  |
| P-*kdpD*-F | GTTTCCCCAGAGACCCACGC |  |
| P-*kdpD-*R | GGATCGGTCGTACTCCGTTG |  |
| P-*kdpF*-F | CGTCGATCCTTCGCCTTGTC |  |
| P-*kdpF-*R | GTGTGCGATCTCTCCTTCGAGC |  |
| P-*algD*-F | TGAAACCCACCTGAAACATTAA |  |
| P-*algD-*R | CGCATTCACCTCGATTGTTTGT |  |
| P-*warA*-F | CGAAAAATTCACCACCCCTTGA |  |
| P-*warA-*R | GACGGGAACTCCAGGATTCATG |  |
| **β-galactosidase activity assay** |  |  |
| pRG970 P-*armZ*-F | GGACTGACCTACCCGGGGATCCCGTTCTCCCTCCAATCGGTT | pRG970Km-P*_armZ_* |
| pRG970 P-*armZ*-R | CTCTAGAAGAAGCTTGGGATCCACATTGAACCTGTAGAGTCAGGGAAAAG | pRG970Km-P*_armZ_* |
| pRG970 P-*oprQ*-F | GGACTGACCTACCCGGGGATCCGTCACGTCCCTCTTTGTCGATCC | pRG970Km-P*_oprQ_* |
| pRG970 P-*oprQ-*R | CTCTAGAAGAAGCTTGGGATCCCTTATTGTTCCTGGTTGTTGGCTTGAT | pRG970Km-P*_oprQ_* |
| pRG970 P-*czcR*-F | GGACTGACCTACCCGGGGATCCTGCGCCGCGGACATTT | pRG970Km-P*_czcR_* |
| pRG970 P-*czcR*-R | CTCTAGAAGAAGCTTGGGATCCGTTCGCCCCTATATAAAGTATGGATTCATTTG | pRG970Km-P*_czcR_* |
| pRG970 P-*eptA*-F | GGACTGACCTACCCGGGGATCCGGAAAGCTTCCCGCGAACT | pRG970Km-P*_eptA_* |
| pRG970 P-*eptA*-R | CTCTAGAAGAAGCTTGGGATCCGGGCACGGATCTTCACTGAC | pRG970Km-P*_eptA_* |
| pRG970 P-*arnB*-F | GACTGACCTACCCGGGGATCCCTCCCCATGTTTCCATGAAGTT | pRG970Km-P*_arnB_* |
| pRG970 P-*arnB*-R | CTCTAGAAGAAGCTTGGGATCCGTAATGAAGCCTCGGTACTGGG | pRG970Km-P*_arnB_* |
| **Gene knockout** |  |  |
| PEX18-*colS*-upstream-F | AACGACGGCCAGTGCCAAGCTTCGGCTATGCCCTGTGCCGGCGC | pEX18Gm-△*colS* |
| PEX18-*colS*-upstream-R | CTCCTTCTTAAAGTTAAACTCGGCTCCTCCGCCAGGCGGT | pEX18Gm-△*colS* |
| PEX18-*colS*-downstream-F | GTTTAACTTTAAGAAGGAGCGAAAAATTCACCACCCCTTG | pEX18Gm-△*colS* |
| PEX18-*colS*-downstream-R | TTCGAGCTCGGTACCCGGGGATGGACGCGTCGATCGTCCGCCTC | pEX18Gm-△*colS* |
| PEX18-*colR*-upstream-F | AACGACGGCCAGTGCCAAGCTTAGTGCGCGCCATGGCCGGTGGA | pEX18Gm-△*colR* |
| PEX18-*colR*-upstream-R | CTCCTTCTTAAAGTTAAACGTCCCACTCCTTCGCAGGAAG | pEX18Gm-△*colR* |
| PEX18-*colR*-downstream-F | GTTTAACTTTAAGAAGGAGGCAGAGCCTCGCCCGCCGGAT | pEX18Gm-△*colR* |
| PEX18-*colR*-downstream-R | TTCGAGCTCGGTACCCGGGGATGCCGCACCTGGCGCGCCAGGCG | pEX18Gm-△*colR* |
| PEX18-*colRS*-upstream-F | AACGACGGCCAGTGCCAAGCTTAGTGCGCGCCATGGCCGGTGGA | pEX18Gm-△*colRS* |
| PEX18-*colRS*-upstream-R | CTCCTTCTTAAAGTTAAACGTCCCACTCCTTCGCAGGAAG | pEX18Gm-△*colRS* |
| PEX18-*colRS*-downstream-F | GTTTAACTTTAAGAAGGAGCGAAAAATTCACCACCCCTTG | pEX18Gm-△*colRS* |
| PEX18-*colRS*-downstream-R | TTCGAGCTCGGTACCCGGGGATGGACGCGTCGATCGTCCGCCTC | pEX18Gm-△*colRS* |
| PEX18-line-F | AGCGCGACGGCCTTCCTCCGCA | pEX18-Gm |
| PEX18-line-R | TTGCGCTCCAGCTGGAAGCCGA | pEX18-Gm |
| **RT-qPCR** |  |  |
| qp-*oprL*-F | CCCGCGAGTACAATATGGCT |  |
| qp-*oprL*-R | GACCGGACGCTCTTTACCAT |  |
| qp-*colS*-F | GATGGTCATCGCCACCTCAT |  |
| qp-*colS*-R | CCAGCAACAGGAAGGTTTGC |  |
| qp-*colR*-F | ATCCGATCGGCCTAAAGCTG |  |
| qp-*colR*-R | TGGCTTGTCGATCACCTGAC |  |
| qp-*amrZ*-F | ACTCCTACCTACTCCAGCCG |  |
| qp-*amrZ*-R | GGCGAACACCGAGATTGTCT |  |
| qp-*eptA*-F | TGCATGTTCTCCAACCTGGG |  |
| qp-*eptA*-R | GGATCCTTGCTCTCGCTCAG |  |
| qp-*czcR*-F | AAGTCAAGACTGCCGACTACC |  |
| qp-*czcR*-R | CCAGTTCGTAAGGGTGTTGC |  |
| qp-*oprQ*-F | TGAACTACACCATCCCGCTG |  |
| qp-*oprQ-*R | GCCAGGCTCCAGATCTTGTT |  |
| qp-*arnB*-F | CGCGATCAAGAACCTGACCT |  |
| qp-*arnB*-R | GGTCGGCCAGGTTGTATTTG |  |
| qp-*mexC*-F | ATGCGGATTTCACCCAGACC |  |
| qp-*mexC*-R | GCCAAGGTGCCTTTCTTCAG |  |
| qp-*mexD*-F | TACACCCTGATCCCGTCCAT |  |
| qp-*mexD*-R | ATGATCCGCTCGACGTTCTC |  |
| qp-*oprJ*-F | ACGAGATCGCCTTCATCGAC |  |
| qp-*oprJ*-R | CTCGATGTACCACCAGGCTC |  |
| qp-*mexF*-F | GGACTTCCTCGACAACCAGG |  |
| qp-*mexF*-R | CAGGACCAGCACGAACTTCT |  |
| qp-*oprN*-F | GGGTCTGTTCAGTCTGCTGG |  |
| qp-*oprN*-R | TTCGACCAACTGGTTCAGGG |  |
| qp-*mexS*-F | CTCAAATGCGAAGAGCTGCC |  |
| qp-*mexS*-R | AGCACATCCTTCCAGCTCAC |  |
| qp-*mexT*-F | TGTTCGAGACCCTGATGCAC |  |
| qp-*mexT*-R | AGGTGGGCGAAGATTTCCTG |  |
| qp-*pmrA*-F | GAGAATACTGCTGGCCGAGG |  |
| qp-*pmrA*-R | ATTCCACGGTATCGCCTTCC |  |
| qp-*pmrB*-F | CAGCACCTTGATGGAGCAGA |  |
| qp-*pmrB*-R | GAGAGTACGTCTTCGGCCAG |  |
| qp-*nfxB*-F | CGCCTGATCAAGGAACACCT |  |
| qp-*nfxB*-R | CCGTGATGTCGATGCGAAAC |  |
| qp-*exoT*-F | GGCTGAACAGGTCGTGAAGA |  |
| qp-*exoT*-R | CTGCCGAACAGGGTGGTTAT |  |
| qp-*pcrH*-F | CGGTCTCTATGAACAGGCCC |  |
| qp-*pcrH*-R | GTAGAAGCCACTCTCGGCTC |  |
| qp-*popN*-F | GAAGAGCTGACCTTCGCCTT |  |
| qp-*popN*-R | TTCTGCTGGCTTTCCAGGTC |  |
| qp-*pcrV*-F | GGTCGACCCCACGCTATATG |  |
| qp-*pcrV*-R | CTCGAAGGGGTACTCATCGC |  |
| qp-*pcrD*-F | AAGTACTCCAGCGGCAACAA |  |
| qp-*pcrD*-R | GAGGAAGGCCTGGGTTATCG |  |
| qp-*gacA*-F | GATTCCCCCTTCGATTCGCT |  |
| qp-*gacA*-R | GGAGATGCTCTGCACCTTGT |  |
| qp-*gacS*-F | TACGGCAGAACCTGGAAACC |  |
| qp-*gacS*-R | CTGCAGCAGGTTGGTGAAAC |  |
| qp-*algB*-F | ATGTCGACCACCTCGGTTTC |  |
| qp-*algB*-R | GGGCTTTCTCCAGGTCTTCC |  |
| qp-*kinB*-F | GAAGCTCGAACTGGCTTCCT |  |
| qp-*kinB*-R | CGTTTTCCAGCAGGTTGTCG |  |
| qp-*cif*-F | CCTCGTTCGAGTACTACCGC |  |
| qp-*cif*-R | AGGGTCATGGTTGGCATCTG |  |
| qp-*algD*-F | AGGAAATCTACCGCGAGCTG |  |
| qp-*algD*-R | TGGCAGATCACGTCCATCAC |  |
