# Supplementary material for: The two-component system ColRS discriminates metal signals to regulate virulence and resistance in Pseudomonas aeruginosa
Source: PLoS Pathog. 2026 Aug 28;22(8):e1014538. doi: 10.1371/journal.ppat.1014538 (PMC13524251; doi:10.1371/journal.ppat.1014538)
Supplement: Supplementary file 16 [file ppat.1014538.s017.docx]

**Response letter**

Dear reviewers,

Please find enclosed our revised manuscript entitled **“The two-component system ColRS discriminates metal signals to regulate virulence and resistance in *Pseudomonas aeruginosa*”**, manuscript number PPATHOGENS-D-26-01235, by Zhao *et. al*.

We thank the reviewers for their valuable time in reviewing our manuscript and the constructive suggestions that they have provided. We have carefully taken these comments into consideration in preparing a revised version for our manuscript, which has resulted in a more thorough and clear manuscript. Please find below a point-by-point response to the reviewers with our responses in **Blue** and the reviewers’ comments in ***Black***.

**Point-by-point response to Reviewer #1**

**Part I - Summary**

*The reviewer has read the responses and the revised version of the manuscript and found that the concerns raised have been satisfactorily addressed. Therefore, the reviewer has no further requests.*

Response: We sincerely thank the reviewer for the careful evaluation of our revised manuscript and for the positive comments. We are delighted that the reviewer considers all previous concerns to have been satisfactorily addressed. We greatly appreciate the reviewer’s insightful comments and valuable suggestions, which have substantially improved our manuscript.

**Point-by-point response to Reviewer #2**

**Part I - Summary**

*The authors describe a novel role for the P. aeruginosa ColRS two-component system in this manuscript. In general, it is well written and most of the data support the conclusions drawn by the authors. The manuscript uses genetics, proteomics and biophysics to identify the genes controlled by ColRS and the mechanisms used by ColRS to respond to zinc and copper. The study is quite novel as it shows the diversity of ColRS responses and shows that copper and zinc are driving the responses. The biophysics (MST) and genetics are convincing in showing a novel Zn/Cu binding site in ColS. The weaknesses of the manuscript are minor The authors have done an exceptional job in responding to the previous reviewer’s concerns.*

Response: We sincerely thank the reviewer for the careful re-evaluation of our manuscript and for the highly positive and encouraging comments. We are delighted that the reviewer appreciates the novelty and significance of our study and considers that the concerns raised previously have been satisfactorily addressed. We greatly appreciate the reviewer’s constructive comments and valuable suggestions throughout the review process, which have significantly improved the quality and clarity of our manuscript.

**Part III – Minor Issues: Editorial and Data Presentation Modifications**

*1. Pg. 5, line 135. Fig. S2A is mentioned in the text before Fig. S1. The order of the figures should be changed.*

Response: We thank the reviewer for pointing this out. To ensure that supplementary figures are cited in sequential order according to their first appearance in the manuscript, we have revised the relevant paragraph and reordered the text so that Fig. S1 is cited before Fig. S2A. The manuscript has been corrected accordingly.

The corresponding description has been included in the revised manuscript (Line 131-140): “Within the *Pseudomonas* genus, ColS and ColR orthologs display strong sequence conservation across species, as shown by Clustal Omega alignment (Fig. S1), suggesting functional conservation of the ColRS system. Phylogenetic analyses demonstrate that ColRS is not unique to *Pseudomonas* but shares significant homology with two-component systems (TCSs) in other critical ESKAPE pathogens (*Enterococcus faecium*, *Staphylococcus aureus*, *Klebsiella pneumoniae*, *Acinetobacter baumannii*, *Pseudomonas aeruginosa* *Enterobacter spp*.) [22, 23] (ColS, ~25-35% identity; ColR, ~35-50% identity; Fig. 1B-C and Fig. S2A), suggesting an ancient, evolutionarily constrained role in bacterial adaptation.”

*2. Pg. 10, line 252; pg. 12, line 297; pg. 20, line 494; pg. 23, line 545; Figure S8, line 95; “qRT-PCR” should be “RT-qPCR”. Reverse transcriptase isn’t quantitative.*

Response: We thank the reviewer for this important correction. We agree that “RT-qPCR” is the more accurate terminology, as reverse transcription itself is not quantitative. Accordingly, we have replaced all occurrences of “qRT-PCR” with “RT-qPCR” throughout the manuscript, including the text on Pg. 10, line 252; pg. 12, line 297; pg. 20, line 494; pg. 23, line 545; Figure S8, line 95. The manuscript has been revised accordingly.

*3. Pg. 16, line 372. Figs. S6G,H are mentioned before S6 panels A-F.*

Response: We thank the reviewer for pointing this out. To ensure that Supplementary Figure S6 panels are cited in sequential order according to their first appearance in the text, we have revised the relevant paragraph and reordered the descriptions so that Fig. S6A-F are cited before Fig. S6G, H. The manuscript has been corrected accordingly.

The corresponding description has been included in the revised manuscript (Line 405-408): “Importantly, the sensor domain construct and all mutant variants displayed comparable purity and solution behavior, with similar size-exclusion chromatography elution profiles and SDS-PAGE patterns, indicating no gross defect in folding or stability (Fig. S6G, H).”

*4. Pg. 27, figure 8; What is the significance for the green and blue ColR and ColS associated arrows? Is the orange “arnB” gene supposed to be blue?*

Response: We thank the reviewer for pointing out that the color scheme and regulatory relationships in Figure 8 were not sufficiently clear. We have revised the figure legend and added additional explanations to improve clarity. Specifically, the green arrows associated with ColS and ColR indicate that expression of the *colS* and *colR* genes is upregulated in response to Zn²⁺ treatment, whereas the blue arrows indicate that expression of these genes is downregulated in response to Cu²⁺ treatment. In addition, the orange color of *arnB* was intentionally used to indicate that the *arnB* promoter is directly regulated by ColR and that this regulation is repressive rather than activating. To avoid confusion, we have clarified this regulatory relationship in the legend of Figure 8.

The corresponding description has been included in the revised manuscript (Line 644-647): “Green arrows indicate transcriptional upregulation of *colS* and *colR* in response to Zn²⁺, whereas blue arrows indicate transcriptional downregulation in response to Cu²⁺. Dark green colored genes (*amrZ*, *eptA*, *oprQ*, and *czcR*) denote direct targets positively regulated by ColR, whereas the orange colored *arnB* gene denotes a direct target negatively regulated by ColR.”

*5. The fig 8 legend says “ColR~P selectively associates with the promoter regions of amrZ,eptA, arnB, oprQ and czcR, thereby promoting their transcription.” Doesn’t the data in Fig. 2I show that arnB was repressed?*

Response: We thank the reviewer for pointing out this inconsistency. The reviewer is correct that our data indicate that *arnB* is negatively regulated by ColR. The statement in the original Figure 8 legend was inaccurate. To accurately reflect both positive and negative regulatory effects of ColR, we have revised the legend to state that “ColR~P selectively associates with the promoter regions of *amrZ*, *eptA*, *arnB*, *oprQ*, and *czcR*, thereby regulating their transcription.” The Figure 8 legend has been corrected accordingly.

We are genuinely grateful for your meticulous review and the opportunity to enhance the manuscript's quality. Your feedback has significantly contributed to refining our work, and we are confident that the revised manuscript effectively addresses your suggestions.

After careful checking and editing, we hope this manuscript will finally meet the standard of ***PLOS Pathogens.*** We thank again for editor and reviews’ very careful review of our manuscript. Thank you very much for all your time and efforts.

Sincerely Yours,

Rui Bao, Ph.D

Center of Infectious Diseases, Division of Infectious Diseases in State Key Laboratory of Biotherapy, West China Hospital, Sichuan University, Chengdu, China

Tel: +86-13540013347

Fax: +86-28-85164060

Email: baorui@scu.edu.cn
